# Identification of Novel CB2 Ligands through Virtual Screening and In Vitro Evaluation

**DOI:** 10.1021/acs.jcim.2c01503

**Published:** 2023-01-24

**Authors:** Adam Stasiulewicz, Anna Lesniak, Magdalena Bujalska-Zadrożny, Tomasz Pawiński, Joanna I. Sulkowska

**Affiliations:** † Department of Drug Chemistry, Faculty of Pharmacy, Medical University of Warsaw, Banacha 1, 02-097 Warsaw, Poland; ‡ Centre of New Technologies, University of Warsaw, Banacha 2c, 02-097 Warsaw, Poland; ¶ Department of Pharmacodynamics, Faculty of Pharmacy, Medical University of Warsaw, Banacha 1, 02-097 Warsaw, Poland

## Abstract

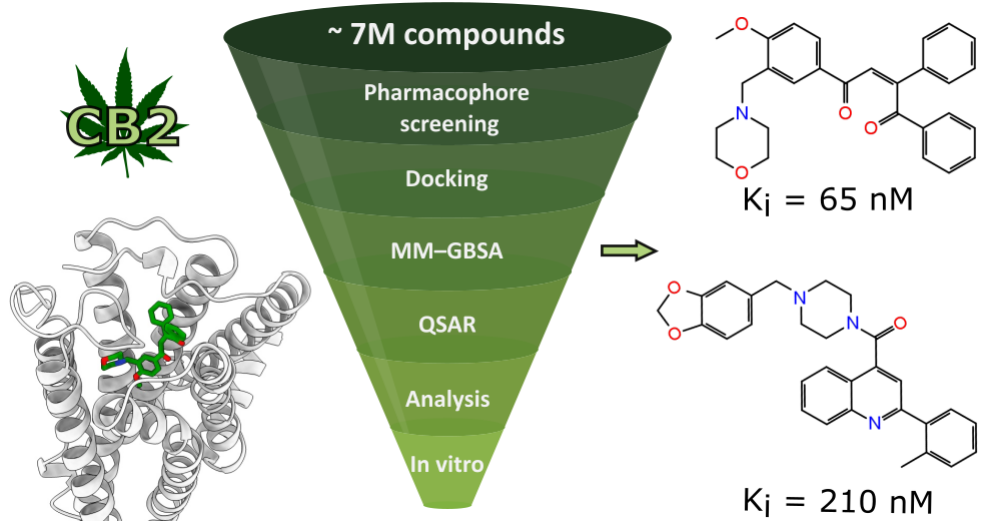

Cannabinoid receptor type 2 (CB2) is a very promising therapeutic target for a variety of potential indications. However, despite the existence of multiple high affinity CB2 ligands, none have yet been approved as a drug. Therefore, it would be beneficial to explore new chemotypes of CB2 ligands. The recent elucidation of CB2 tertiary structure allows for rational hit identification with structure-based (SB) methods. In this study, we established a virtual screening workflow based on SB techniques augmented with ligand-based ones, including molecular docking, MM–GBSA binding energy calculations, pharmacophore screening, and QSAR. We screened nearly 7 million drug-like, commercially available compounds. We selected 16 molecules for in vitro evaluation and identified two novel, selective CB2 antagonists with *K*_i_ values of 65 and 210 nM. Both compounds are structurally diverse from CB2 ligands known to date. The established virtual screening protocol may prove useful for hit identification for CB2 and similar molecular targets. The two novel CB2 ligands provide a desired starting point for future optimization and development of potential drugs.

## Introduction

The chemical constituents of *Cannabis sativa* exhibit their pharmacological activity via the endocannabinoid system (ECS),^1^ which is significant for regulation of various physiological and pathophysiological processes in the human organism.^2^ The regulatory functions of ECS are conveyed by ligands binding to cannabinoid receptors (CBRs) of which type 1 (CB1) and type 2 (CB2) are the most prevalently studied. Both are very promising therapeutic targets for multiple possible indications, and thus they have been the subject of a great deal of scientific interest, also regarding harnessing their potential in medicine.^3^ In recent years, CB2 is becoming more eagerly explored,^4,5^ in large part due to the potentially more favorable pharmacological profile of compounds modulating CB2 activity.^6^ Despite multiple selective CB2 ligands having been designed, none of the drugs containing such compounds have yet reached approval. Therefore, there is still a huge potential in discovering novel CB2 ligands. The recent determination of CB2 tertiary structure by Li et al. in 2019^7^ opened a great possibility to conduct a rational, computer-aided design of new compounds modulating CB2 activity.

CB1 and CB2 are relatively similar G-protein-coupled receptors (GPCRs).^7,8^ The main difference between the two receptor types lies in their localization in the human organism. CB1 is distributed primarily in the central nervous system (CNS)^9^ but also in many other regions, including various organs.^10^ CB2 is located mainly in the immune system,^11^ but it is also present in bones, skin,^2^ and brain.^10^ Thus, the activation of CB1 and CB2 leads to a different range of pharmacodynamical effects, which have been tried to be utilized in medicine. This is possible due to the slightly diverse structure of the binding site in these two proteins.^8^ This in turn, allows for the design of ligands with varying levels of selectivity.

There have been numerous attempts to utilize substances acting via CBRs, especially CB1, in pharmacotherapy, including both phytocannabinoids^12^ and specifically designed, synthetic compounds.^3^ They proved to be effective or are studied for their potential use for multiple indications, e.g., pain,^13^ nausea,^14^ obesity,^15^ and many others. However, as ECS is a multipurpose system, the utilization of CB1 ligands comes with a different rate or severity of adverse effects. This entails addiction^16^ or cognitive impairment^17^ for CB1 agonists or anxiety and depression for antagonists/inverse agonists that disrupt CB1 signaling.^15^

Multiple solutions have been proposed to target ECS in a safer manner. Most of the serious adverse effects are related to modulation of CB1 activity within the CNS, especially by compounds that alter CB1-dependent transmission in the most severe manner, such as inverse agonists.^18^ Thus, the most prominent strategies include the design of peripherally restricted CB1 ligands,^19^ neutral antagonists,^20^ or allosteric modulators.^21^ Moreover, striving to target other ECS components, such as monoacylglycerol lipase (MAGL)^22^ or transient receptor potential vanilloid 1 (TRPV1) channel,^23^ has been growing steadily in recent years. Nevertheless, the most intense focus is directed toward designing new CB2 ligands.

Some of the therapeutic effects of CB1 activity modulation may be achieved by targeting CB2. The potential shared indications for CB1 and CB2 agonists include pain,^24^ anxiety,^25^ neurodegenerative disorders,^26^ cancer,^27^ emesis, and nausea.^28^ Of note, there are some conditions such as addiction,^29^ systemic sclerosis,^30^ atherosclerosis,^31^ obesity,^32^ or diabetes,^33^ where CB1 antagonists can be successfully replaced with CB2 agonists without seriously compromising the therapeutic outcome. What is more, in some diseases that are characterized by a prominent inflammatory component (e.g., rheumatoid arthritis or osteoarthritis), CB2 activation may seem the preferred mechanism of action.^34−36^ Finally, CB2 antagonists could aid in tackling some conditions like renal fibrosis^37^ and immunoparalysis.^38^ Additionally, apart from the broad spectrum of potential indications, CB2 ligands lack the typical CB1-related psychotropic adverse effects,^6^ which makes them desired potential drug candidates.

Several drugs containing active ingredients nonselectively targeting CB1 and CB2 have already been approved.^3^ CBRs’ ligands present in those drugs include cannabinoids such as dronabinol (present in Marinol)^39^ or nabilone (Cesamet).^40^ Moreover, nabiximols, the *Cannabis*-based extract containing tetrahydrocannabinol and cannabidiol, is approved as Sativex.^41^ Nevertheless, because of the great potential in selective CB2 targeting, there have already been multiple attempts in the design of its specific ligands, including quite a few successful ones. Some compounds have even reached clinical trials.^42,43^ However, structurally diverse CB2 ligands may exhibit considerably different characteristics. They possess various physicochemical properties, resulting in disparities in absorption, distribution, metabolism, and excretion (ADME) parameters. Moreover, the diversity of functional groups may lead to differences in toxicity. Also, CB2 ligands exhibit varying selectivity and a full range of intrinsic activities, including protean agonism.^44^ Furthermore, CB2 agonists also show varied degrees of signaling bias.^45,46^ What is more, CB2 is able to form functionally relevant heterodimers, e.g. with CB1,^47^ G-protein-coupled receptor 55 (GPR55)^48^ or C-X-C chemokine receptor type 4 (CXCR4).^49^ Multiple nuances surrounding the issue of CB2 modulation indicate that multiple attempts might be needed to find CB2 ligands suitable for effective and safe pharmacotherapy. In order to achieve this goal, rational and time-efficient tools for the discovery of novel CB2 ligands need to be established.

**Figure 1:**
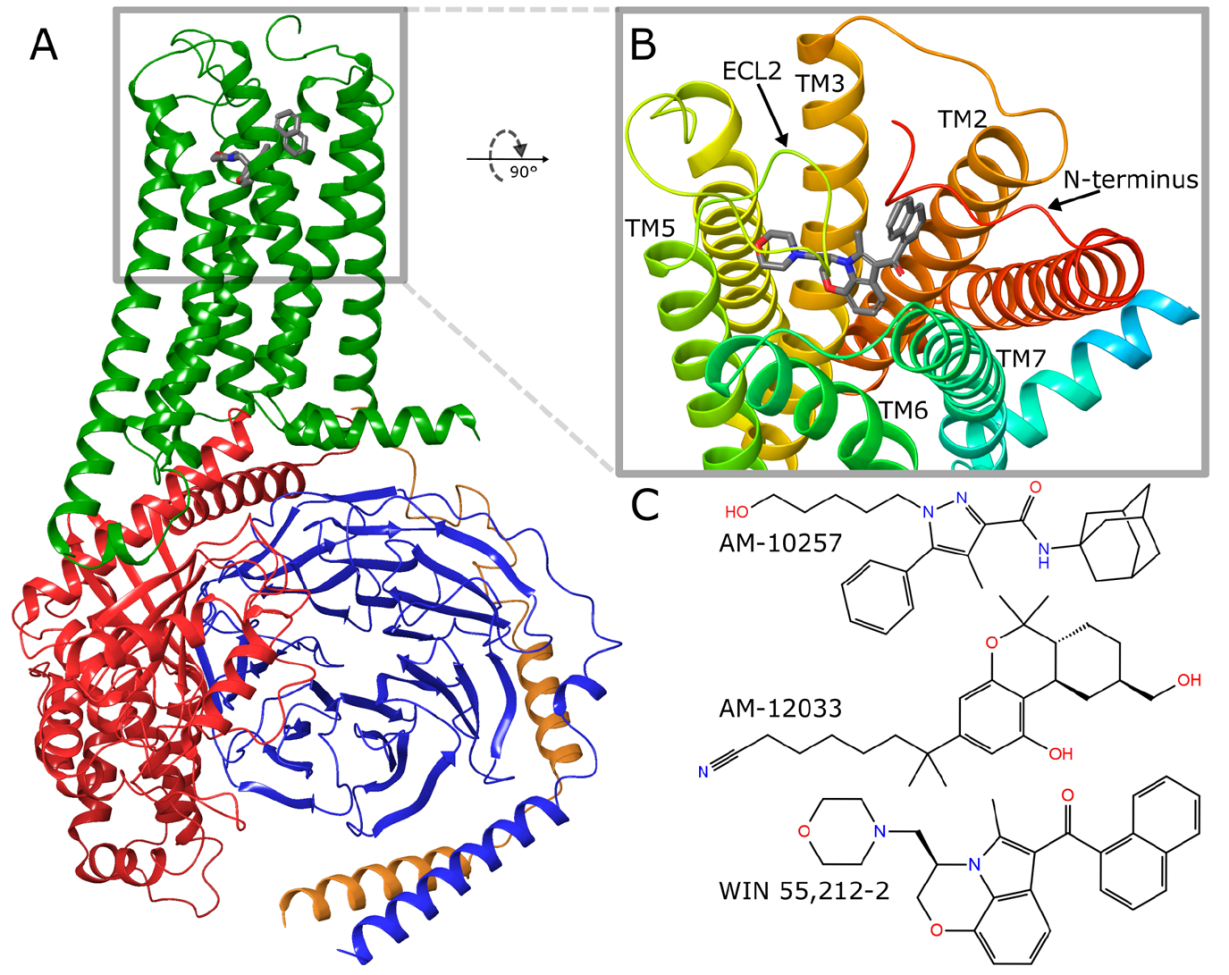
Overview of CB2 based on PDB ID 6PT0. (A) CB2–ligand–G-protein complex. CB2, green. G-protein subunits: α, red; β, blue; γ, orange; ligand, gray (stick representation). (B) CB2 orthosteric binding site—view from the extracellular side with marked structural elements responsible for ligand binding. TM, transmembrane helix; ECL2,extracellular loop 2. (C) Ligands present in PDB-deposited CB2 structures: AM-10257 (PDB ID 5ZTY), AM-12033 (6KPC, 6KPF), and WIN 55,212-2 (6PT0).

Development of novel CB2 ligands has recently become more approachable because of the determination of this receptor’s tertiary structure in 2019.^7^ Although CB2 is member of well-studied class A GPCRs (Figure 1A), its orthosteric binding site (Figure 1B) is highly hydrophobic, which makes CB2 a nontrivial molecular target. To date, there are four CB2 structures deposited in the Protein Data Bank (PDB)^7,50,51^ with three different ligands of various chemical structures (Figure 1C) and intrinsic activities (Table 1), which in turn imposes diversified conformations of the CB2 orthosteric binding site. This allows for rational utilization of structure-based (SB) computational methods to screen for or design novel CB2 ligands. However, the high hydrophobicity of the CB2 binding site is a crucial obstacle that could compromise the effective employment of SB techniques. So far, there have been multiple in silico studies based on ligand-based (LB) methods^52,53^ or using CB2 homology models.^54−56^ However, because of the very recent elucidation of the CB2 structure, there is still a lack of studies with optimal SB or mixed structure/ligand-based approach to the prediction of new CB2 ligands. Thus, the development of novel, modern, computational procedures will be very beneficial for future projects focusing on compounds targeting CB2.

**Table 1:** CB2 Structures Deposited in PDB^a^

PDB ID	ligand	intrinsic activity	G-protein	method	resolution (Å)	ref
5ZTY	AM-10257	antagonist/inverse agonist	–	XRD	2.8	(7)
6KPC	AM-12033	agonist	–	XRD	3.2	(50)
6KPF	AM-12033	agonist	+	cryo-EM	2.9	(50)
6PT0	WIN 55,212-2	agonist	+	cryo-EM	3.2	(51)

a XRD, X-ray diffraction; cryo-EM, cryoelectron microscopy.

Herein, we present a successful, computational approach to finding novel CB2 ligands. We have developed and validated a multistep, in silico screening workflow. Due to the elucidated CB2 structure and known ligands, we combined SB and LB techniques. Implementation of SB methods allowed for moving beyond known chemotypes, while combining LB methods—to reduce the chance of achieving false positive outcomes. Using the computational procedure established here, we screened a nearly 7 M drug-like compound library and verified the results with cell-based displacement binding, selectivity, and functional assays. We identified two novel, selective CB2 anatgonists, structurally diverse from compounds known to-date. Moreover, our study provides an effective procedure of in silico screening for new CB2 ligands, along with insights that may be useful for other similar molecular targets.

## Methods

### Pharmacophore Screening

The pharmacophore screening was conducted in LigandScout 4.4.4.^57^ Initially, we generated multiple pharmacophores based on PDB IDs 5ZTY,^7^6KPC, 6KPF,^50^ and 6PT0.^51^ We tested various options, including complex pharmacophores based on more than one PDB-deposited structure. We created them using different settings, including merged or shared features (descriptors) and superposition on features or reference points (amino acids).

The created pharmacophores were validated using a test set of 20 compounds with *K*_i_ values <100 nM toward human CB2 according to previously reported in vitro studies (Table S1) and a Schrödinger decoy set of 1000 drug-like compounds (average molecular weight (MW) = 400 g/mol).^58^ Active compounds were not discriminated for their intrinsic activities, based on the insights of Markt et al.^52^ and Brogi et al.^59^ and because of the inability to adequately represent the Trp258 toggle switch^60,61^ in pharmacophores. Structures of the 20 active test compounds were downloaded from PubChem.^62^ The compound sets were prepared in LigandScout using idbgen with iCon high-throughput conformer generation with maximum number of conformations set to 100. The validation consisted of screening both sets using generated pharmacophores with Get Best Matching Conformations retrieval mode. We tested different values of Maximum Number of Omitted Features (Table S2).

**Figure 2:**
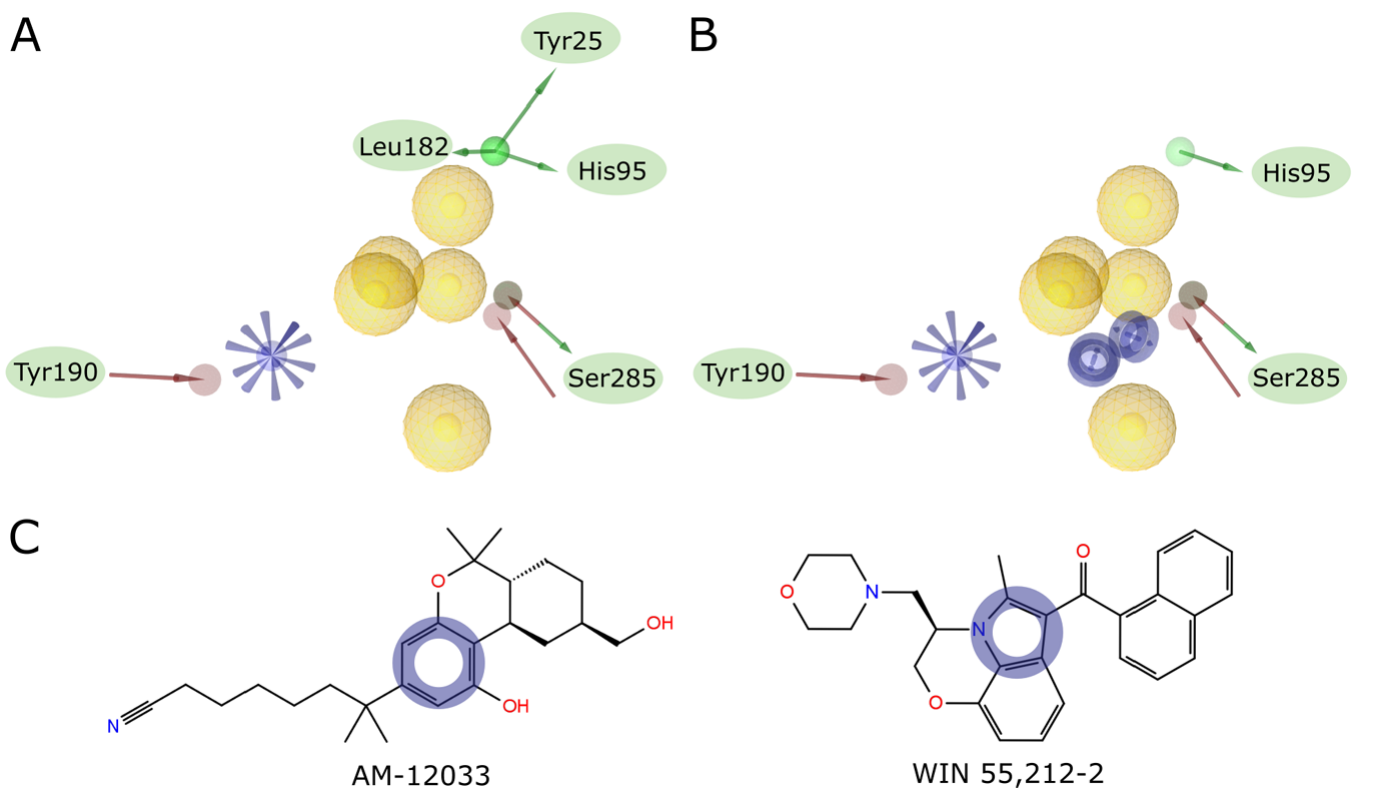
(A) Complex pharmacophore created by merging pharmacophores based on PDB IDs 6KPC and 6PT0 after superposition on reference points. (B) Pharmacophore from panel A with manual modifications introduced to account for frequent occurrence of aromatic rings in the center of the ligand and rare H-bonds. Green arrow, H-bond donor; red arrow, H-bond acceptor; yellow sphere, hydrophobic; blue star, positive ionizable; blue ring, aromatic ring. (C) Structural formulas of the two ligands used to create the pharmacophore. Placement of manually added aromatic ring descriptors is shown as blue circles.

We retrieved three well-functioning, combined pharmacophores based on PDB IDs 6KPC and 6PT0; 5ZTY, 6KPC, and 6PT0; and 5ZTY, 6KPC, 6KPF, and 6PT0. All were generated using the same options: merging features and superimposition on reference points. Then, according to the known structures of CB2–ligand complexes,^7,50,51^ we implemented manual, rational changes to these complex pharmacophores or their constituent pharmacophores in order to account for the divergence between models automatically generated by LigandScout and the experimental data. We obtained the best results for the pharmacophore based on PDB IDs 6KPC and 6PT0 (Figure 2). This model was additionally tested with modified set of active compounds (Table S3) to confirm its suitability for screening (Figure S1).

We downloaded 6 876 667 drug-like compounds (log *P* < 5; MW < 500 g/mol) from the ZINC database.^63^ The library was prepared in LigandScout using idbgen, with the same settings as test sets used for validation. The library was then screened using the best aforementioned pharmacophore with Maximum Number of Omitted Features set to nine.

### Molecular Dynamics

CB2 structures based on PDB IDs 5ZTY, 6KPC, and 6PT0 were initially prepared in BIOVIA Discovery Studio v20.1.0.19295.^64^ Water molecules and other redundant, postcrystallization small molecules were deleted. The substitutions from the crystal structures were reverted to the wild-type version (Supporting Information). In the case of PDB IDs 5ZTY and 6KPC, we deleted the T4-lysozyme fusion protein in the place of the intracellular loop 3 (ICL3). In the PDB ID 6PT0, the G-protein was deleted. We prepared the structures using Prepare proteins protocol with pH = 7.4 and CHARMm force field.^65^ Additionally, for PDB IDs 5ZTY and 6KPC, we rebuilt the ICL3 in Prepare Proteins protocol, using a human CB2 sequence from UniProt.^66^

In the next phase, CB2–membrane complexes for the molecular dynamics (MD) were prepared using CHARMM-GUI Membrane Builder.^67^ The systems were expanded with 1-palmitoyl-2-oleoylphosphatidylcholine (POPC) bilayer, TIP3P water molecules, and 0.15 M NaCl. We selected CHARMM36 force field.^68^ The ligands were parametrized using ParamChem server in CHARMM General Force Field (CgenFF).^69^

The MD simulations were conducted in GROMACS 2018.8.^70^ We performed steepest-descent energy minimization and six phases of equilibration. Then, a single run of 1 μs production for each of the CB–ligand complexes or apo-CB2 was conducted. For obtained trajectories, we calculated root-mean-square deviation (RMSD) of CB2 C_α_ atoms after superposition on the same atoms of the first frame of the trajectory. Additionally, we calculated RMSD of heavy atoms of the ligands in the case of three CB2–ligand simulations, after the same superposition on CB2 C_α_ atoms (Figure S2). The results showed that simulations were stable and suitable for further utilization. Obtained trajectories were clustered using gmx cluster with gromos algorithm.^71^ Clustering was based on heavy atoms of amino acids within 5 Å of ligands in PDB IDs 5ZTY, 6KPC, and 6PT0. Additionally, we conducted replica simulations to confirm the suitability of the aforementioned production runs (Table S4, Figures S3–S5, discussed in the Supporting Information). Details regarding MD equilibration, simulations, clustering, and replicas are described in the Supporting Information.

### Docking and MM–GBSA

Docking and molecular mechanics–generalized Born surface area (MM–GBSA) binding energy calculations were conducted in Schrödinger Maestro 2017-1.^72^ CB2 structures were prepared using Protein Preparation Wizard. We removed water molecules and other redundant, postcrystallization small molecules. The structures were minimized in the OPLS3 force field.^73^ Ligands were prepared in LigPrep. Docking was conducted in Glide with standard precision (SP).^58,74^ MM–GBSA binding energy calculations were performed using Prime, with VSGB solvation model^75^ and OPLS3 force field.

Initially, we validated the docking procedure. In order to test its ability to predict correct binding pose, we conducted redocking and cross-docking for all available CB2 PDB-deposited structures, PDB IDs 5ZTY, 6KPC, 6KPF, and 6PT0. Then, we calculated RMSD for ligands’ heavy atoms (Table S5). In the case of cross-docking, we had prior superimposed the complexes on the C_α_ atoms of the receptor, with the exclusion of the ICL3 or hybrid protein in its place.

Next, we tested the procedure’s ability to correctly predict ligands’ binding affinities. For this purpose, we created a test set of 40 ligands with different *K*_i_ values toward human CB2 known from in vitro studies (Table S6). The compounds’ structures were downloaded from PubChem. We docked this library to CB2 models based on the four PDB structures, as well as on the central structures of 25 clusters from MD. Additionally, we conducted MM–GBSA binding energy calculations. We calculated Pearson correlation coefficients (R) between obtained docking scores or MM–GBSA CB2–ligand binding energies (Δ*G*_bind_) and p*K*_i_ values (Figure S6). Additionally, we performed confirmatory validation of binding affinity prediction for the best models. For this purpose, we utilized a modified test set with a greater structural variety of compounds (Table S7).

### Physicochemical Properties Filtration

We calculated the physicochemical properties of the selected compounds using Schrödinger QikProp. Then, we filtered compounds with desired physicochemical properties, based on Lipinski’s^76^ and Veber’s^77^ rules, with the log *P* value modified to accommodate high hydrophobicity of CBRs’ ligands. The specific values: MW ≤ 500 g/mol, log *P* ≥ 3, number of hydrogen bond acceptors ≤ 10, number of hydrogen bond donors ≤ 5, number of rotatable bonds ≤ 10, and polar surface area (PSA) ≤ 140 Å^2^.

### QSAR

We conducted the quantitative structure–activity relationship (QSAR) part of the study using Schrödinger AutoQSAR.^78^ All compounds with known *K*_i_ values toward human CB2 deposited in ChEMBL^79,80^ (3695 nonrepeating molecules) were retrieved and prepared using Schrödinger LigPrep. For model generation, random training set was set to 75% compounds and prediction property—to p*K*_i_. During screening, we retained the compounds with domain alert = 0.

### CB2 Radioligand Displacement Assay

The compounds selected for the in vitro binding assay were purchased via MolPort, SIA, Riga, Latvia (Supporting Information S2). Membrane preparations from CHO-K1 cells expressing the human CB2 (ChemiSCREEN Membrane Preparation Recombinant Human CB2 Cannabinoid Receptor. Merck, USA) were incubated in duplicate with 0.8 nM [^3^H]CP-55,940 (specific activity: 101 Ci/mmole, PerkinElmer, USA) in a 50 mM Tris–HCl, pH = 7.4 buffer supplemented with 2.5 mM EDTA, 5 mM MgCl_2_, 0.5 mg/mL BSA and increasing concentrations of the compounds tested. Compounds were dissolved in 50% DMSO and added to the reaction mixture at 10 concentrations equally spaced on a log scale (10^–10^–10^–4.5^ M). The final DMSO concentration was 5%. Nonspecific binding was determined with 10 μM WIN 55,212-2. The reaction mixture (500 μL) was incubated for 1.5 h at 30 °C. Before harvesting, Brandel Whatman GF/B Filter Paper was presoaked with 0.5% polyethylenimine buffer for 30 min and then washed with 2 mL of 50 mM Tris–HCl buffer (pH = 7.4) containing 0.5% BSA to minimize nonspecific binding. The reaction was terminated by depositing the samples onto the filter paper with the Brandel M–24 Cell Harvester. Samples were then rapidly washed three times with 2 mL of wash buffer (50 mM Tris–HCl pH 7.4, 2.5 mM EDTA, 5 mM MgCl_2_, 0.5 mg/mL BSA) to separate the bound radioligand from free. Filters were then air-dried for 1.5 h at 60 °C. After drying, filter discs were placed on a flexible 24-well plate and 500 μL of EcoScint-20 scintillant (PerkinElmer, USA) was added to each well. Plates were counted (2 min per well) in a Trilux MicroBeta counter (PerkinElmer, USA). Data were analyzed with GraphPad Prism 5.0 software. Curves were fitted with a one-site nonlinear regression model, and inhibitory constants (p*K*_i_ ± SEM and *K*_i_, 95% CI) were calculated from the Cheng–Prusoff equation.

### CB1 Radioligand Displacement Assay

Membrane preparations from Chem-1 cells expressing the human CB1 receptors (ChemiSCREEN CB1 Cannabinoid Receptor Membrane Preparation. Merck, USA) were incubated in duplicate with 1 nM [^3^H]CP-55,940 (specific activity: 108.5 Ci/mmole, PerkinElmer, USA) in a 50 mM Tris–HCl, pH = 7.4 buffer supplemented with 1 mM CaCl_2_, 5 mM MgCl_2_, 0.2% BSA and increasing concentrations of the compounds tested. Compounds were dissolved in 50% DMSO and added to the reaction mixture at 10 concentrations equally spaced on a log scale (10^–10^–10^–4.5^ M). The final DMSO concentration was 5%. Nonspecific binding was determined with 10 μM WIN 55,212-2. The reaction mixture (500 μL) was incubated for 1.5 h at 30 °C. Before harvesting, Brandel Whatman GF/B Filter Paper was presoaked with 0.5% polyethylenimine buffer for 30 min and then washed with 2 mL of 50 mM Tris–HCl buffer (pH = 7.4) containing 0.5% BSA to minimize nonspecific binding. The reaction was terminated by depositing the samples onto the filter paper with the Brandel M-24 Cell Harvester. Samples were then rapidly washed three times with 2 mL of wash buffer (50 mM Tris–HCl pH 7.4, 500 mM NaCl, and 5 mM MgCl_2_) to separate the bound radioligand from the free one. The rest of the procedure was the same as for CB2.

### CB2 [^35^S]GTPγS Assay

Ten micromolar concentrations of each compound were incubated in triplicate with membrane preparations from CHO-K1 cells expressing the human CB2 receptor (0.5 μg per well) (PerkinElmer, Cat. No. ES-111-M400UA) in an assay buffer containing 50 mM Tris–HCl, pH = 7.4, 0.2 mM EGTA, 3 mM MgCl_2_, 100 mM NaCl, 30 μM GDP and 1 mg/mL BSA) in the presence of 0.08 nM [^35^S] guanosine 5′-[γ-thio]triphosphate ([^35^S]GTPγS) (specific activity: 1250 Ci/mmole, PerkinElmer). Nonspecific binding was determined with 100 μM of unlabeled GTPγS. CP-55,940 (100 nM) was used as stimulating ligand. The final DMSO concentration in the assay was 5%. The reaction mixture was incubated for 60 min at 30 °C. Next, the samples were deposited under vacuum with the FilterMate Harvester (PerkinElmer, USA) onto Unifilter GF/B Plates (PerkinElmer, USA) presoaked with wash buffer (50 mM Tris–HCl, pH = 7.4). The samples were then rapidly washed with 2 mL of wash buffer. Filter plates were dried for 30 min at 50 °C and 40 μL of MicroScint PS (PerkinElmer, USA) scintillation fluid was added to each well. Radioactivity was counted in a Trilux MicroBeta^2^ counter (PerkinElmer, USA). Data were analyzed with GraphPad Prism 5.0 software. Results were expressed as percent of basal [^35^S]GTPγS binding in the presence of CP-55,940 from three separate experiments. Basal binding was set to 100%.

## Results and Discussion

### Virtual Screening for Novel CB2 Ligands

Cannabinoid receptors are nontrivial targets for computational SB drug design (SBDD) because of the high hydrophobicity of the orthosteric binding sites. In silico methods that are often used for the screening campaigns, such as molecular docking, usually struggle with cases in which protein–ligand binding modes are determined mainly by multiple nonspecific, hydrophobic interactions. As this is the case with CBRs, effective utilization of docking is a challenging task, as there may be issues with both proper pose and binding affinity prediction.^81^

On the other hand, LB drug design (LBDD) methods might overcome the aforementioned problems with hydrophobicity. However, the nature of LB techniques makes finding novel chemotypes a difficult task. Thus, in the case of in silico studies regarding structurally diverse compounds and highly hydrophobic molecular targets, combining SB and LB methods seems to be an optimal solution, as it derives the accuracy from the LBDD and the ability to identify new chemotypes from SBDD. Moreover, merging methods from both approaches reduces the risk of false positive outcomes, which is a big problem in the case of inaccurate and semiaccurate virtual screening (VS) techniques.^82,83^

**Figure 3:**
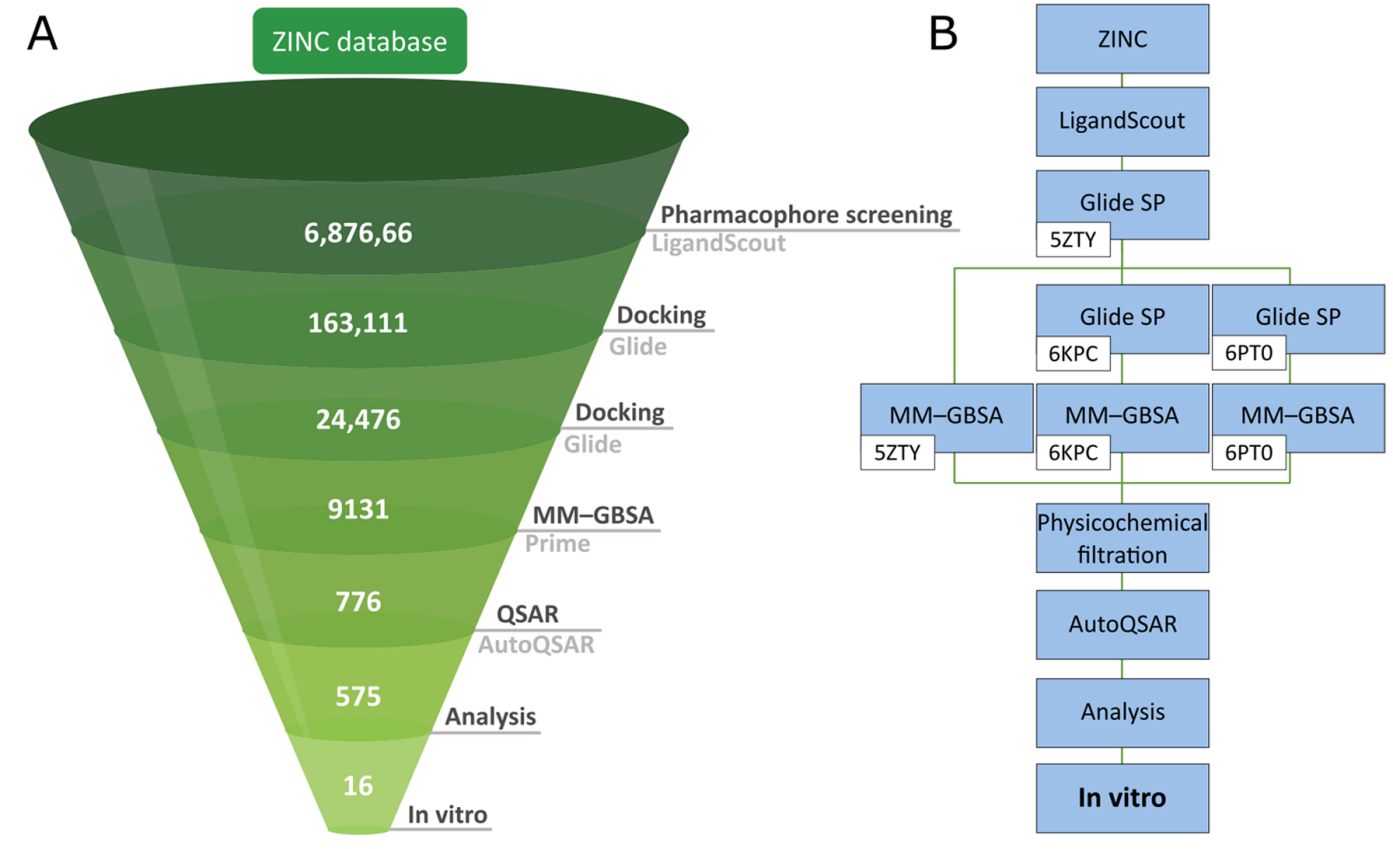
Schemes of the workflow used in this study. (A) Main steps employed in the screening along with the number of compounds left after each step. (B) A scheme showing the detailed order of utilized techniques, especially docking to CB2 structures from PDB IDs 5ZTY and 6KPC and to the CB2 model based on MD of PDB ID 6PT0.

In this paper, we show an effective combination of SB and LB methods employed for VS aimed to identify novel CB2 ligands (Figure 3, thresholds used for specific methods are summarized in Table S8). As a screening set, we selected a nearly 7M drug-like compound library from the ZINC database. Using a multistep procedure specifically created and validated for this molecular target, we narrowed down the set to 16 candidates that we further tested in the in vitro binding assay and found novel CB2 ligands.

#### Pharmacophore Screening

**Figure 4:**
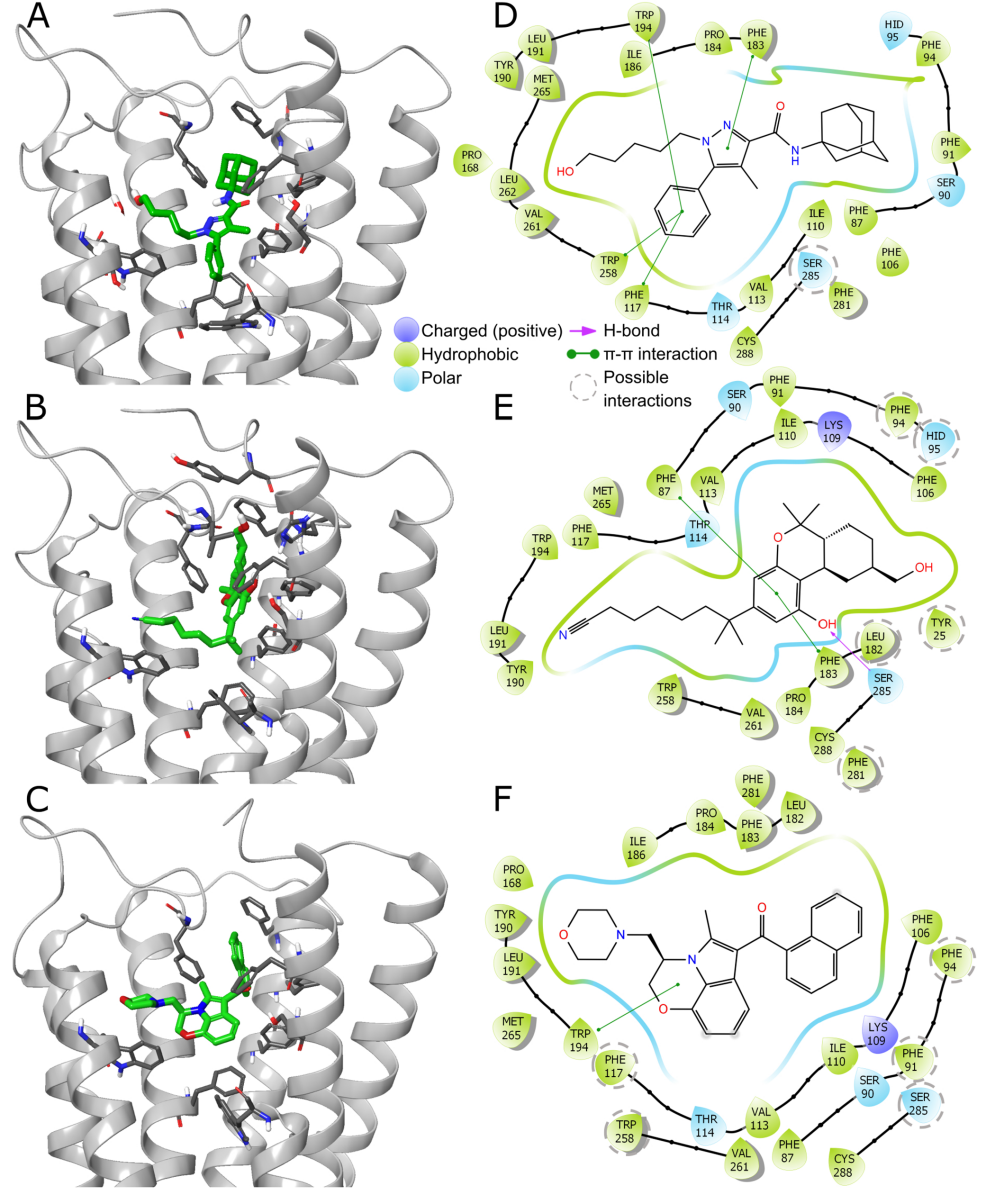
CB2–ligand complexes. (A–C) Binding sites with ligands (green) and amino acids (gray) important for ligand binding depicted in stick representation. PDB IDs 5ZTY, 6KPC, and 6PT0, respectively. (D–F) 2D interaction schemes generated using Schrödinger Maestro. Additionally, we marked with gray, dashed circles the amino acids that are too far away from the ligand to create protein–ligand interactions in deposited structures but probably do so alternately, for limited periods of time in natural, nonstatic complexes.

The first step of our workflow consisted of the initial pharmacophore screening in LigandScout. We created a hybrid, structure/ligand-based model by merging two constituent pharmacophores generated from PDB IDs 6KPC and 6PT0 (Figure 2A). Additionally, because of the abundance of π–π interactions and rare H-bonds in the known CB2–ligand complexes (Figure 4), we manually added two aromatic ring descriptors and removed the excessive number of H-bonds (Figure 2B). The aromatic ring descriptors were placed in the positions of aromatic rings present in AM-12033 and WIN 55,212-2 (Figure 2C). The initial pharmacophore proposed by LigandScout included three H-bond donor descriptors for one of the hydroxyl groups of AM-12033 (Figure 2A). However, those interactions are not present in the crystal structure (Figure 4B,E). Thus, those three descriptors would put an unjustified focus on hydrogen bonding near the N-terminus, which is also contrary to the data obtained from the analysis of other PDB-deposited CB2 structures (Figure 4). As H-bonds in this area are not present in the known CB2 structures, but Tyr25, His95, and Leu182 are in such proximity that the interactions are possible for slightly different binding site conformations (Figure 4D–F), we decided to leave only one H-bond donor descriptor for His95 (Figure 2B). The choice of a merged model, along with allowing the screening algorithm to omit a limited number of descriptors for compounds to fit into, gave us the advantage of lesser restrictivity toward a single chemotype.

Based on the comprehensive analysis of the validation results (Figure S1), we proposed a cutoff for the Pharmacophore-Fit function value: >60. This threshold allowed for high enrichment, retaining a number of compounds feasible for computationally more costly methods. Using the determined cutoff, we selected over 160 000 candidates for the next phase.

#### Initial Docking

The second main part of the study was based on docking using Schrödinger Glide. First, because of the large number of compounds retrieved from the pharmacophore screening, we performed a rough prediction of the potential CB2 ligands by docking to a single CB2 structure using Glide SP with a mild cutoff for the scoring function.

For this step we chose a CB2 model based on PDB ID 5ZTY, as the only available inactive conformation. Its binding site’s volume is thus greater than for other CB2 structures, mainly because of the shift of transmembrane helix 1 (TM1), but also due to the conformation of Trp258,^60^ maximizing the capability to sterically fit the potential ligands, regardless of their functional activity. The smaller volume of the CB2 active conformation’s orthosteric binding site is a discriminatory factor between agonists and antagonists, hindering the sterical fit of the latter because of their larger size. On the other hand, the greater volume of the inactive site allows for the placement of ligands from both intrinsic activities. However, as docking agonists to the inactive conformation is possible, it is not optimal because of the relatively too large volume of the pocket. Fortunately, the CB2 inactive site is far more similar to the active one than in the case of the corresponding pair for CB1.^7,50^ This allows for obtaining sufficiently good results also in the case of docking potential agonists to 5ZTY. The capability of the proper pose prediction for ligands with various chemotypes and intrinsic activities docked to 5ZTY was proved by cross-docking of ligands from other CB2 PDB-deposited structures (Table S5), which was conducted as a part of validation.

The cutoff for this part of the screening procedure was set to docking score ≤ –9. This value was based on the validation results of binding affinity prediction for docking to PDB ID 5ZTY (Figure S6A). The selected threshold filtered out test compounds mainly with *K*_i_ values <100 nM. After this step of the screening procedure, we retrieved over 24,000 compounds that we further subjected to more rigorous docking.

#### Docking and MM–GBSA

Before the virtual screening, we had conducted a comprehensive docking validation, during which multiple combinations of CB2 structures, scoring functions, and settings had been tested. Although there are four CB2 structures with three unique ligands available in the PDB, we wanted to explore the conformational space of the binding site even further. As shown in Figure 4D–F, several amino acids are too far away from the ligands to create interactions in the PDB-deposited structures, but they are in such a proximity that they could form the interactions after minor conformational shifts.

New CB2 structures generated using MD, with different binding site architecture and subsequently slightly different binding modes possible for the ligands, would provide more receptor models for docking. For this purpose, we performed MD simulations of CB2–ligand complexes from PDB IDs 5ZTY, 6KPC, and 6PT0. Also, we conducted MD simulations of apo-CB2 based on active (6PT0) and inactive (5ZTY) conformations of the receptor. For each of them, we obtained a 1 μs trajectory that we further clustered. We retrieved five CB2 conformations from the most populated clusters from each simulation, giving the total of 25 new CB2 models.

The new CB2 models were subjected to validation of docking binding affinity prediction along with four PDB-deposited structures. The results showed that the structures from the biggest clusters performed similarly well to the PDB-deposited ones. As we moved to the less populous clusters, the test results usually grew further apart from the in vitro data. As predicted, the apo-structures performed far worse and in many cases test ligands were even unable to fit into the binding site. It shows a considerable induced-fit effect that is necessary for obtaining amino acid conformations suitable to later conduct rigid-protein docking.

On the grounds of validation results, we conducted Glide SP docking to two more CB2 models, based on 6KPC PDB-deposited structure and on the central structure of the first cluster derived from the MD of CB2–ligand complex from PDB ID 6PT0. Cross-docking to CB2 PDB-deposited structures showed that in many cases the docking algorithm is unable to properly predict binding pose for a ligand from a specific chemotype at a binding site from CB2 structure determined with a ligand from other structural group (Table S5). This is an issue especially for CB2 active conformations. One of the more viable strategies to overcome this problem in VS is to conduct docking to multiple CB2 structures with varying conformations of the binding site. As one compound could have completely different binding poses proposed for specific CB2 conformation, considering only best-scored pose is a superior strategy compared to using average values. This approach proved to be effective by validation, providing a better correlation with experimental results than for a single CB2 structure (Figure S6B). Based on the validation results, we proposed a cutoff for docking score: ≤−10 for at least one of the three CB2 models used (including PDB ID 5ZTY), which corresponded to most of the test compounds, fulfilling this criterion, possessing *K*_i_ values <100 nM. Using this threshold, we filtered out around 9000 compounds. In the case of one ligand obtaining desired score for more than one CB2 model, we took into account all complexes of such candidate.

In the next step, we estimated protein–ligand binding energies using the MM–GBSA method. We selected 776 compounds with Δ*G*_bind_ ≤ −80 kcal/mol in the case of compounds docked to 5ZTY and 6KPC and ≤−90 kcal/mol for the 6PT0 MD-derived model. The threshold of −80 kcal/mol was established similarly to the cutoff for docking score (Figure S6C). The modification of the value for 6PT0 was caused by considerably higher number of compounds obtaining −80 kcal/mol than for the two other CB2 structures, which led to a need for tightening the threshold.

#### Physicochemical Properties Filtration and QSAR

The next phase of the VS consisted of LB prediction of the selected candidates’ p*K*_i_ values. For this purpose, we utilized Schrödinger AutoQSAR. Based on the ChEMBL-deposited compounds with known *K*_i_ values toward human CB2, we created ten QSAR models (Table S9 and Figure S7). Before their implementation in VS, we had calculated physicochemical properties of the remained compounds and filtered out those that fulfill the Lipinski’s and Veber’s rules and additionally obtain log *P* ≥ 3 to account for the high hydrophobicity of the CBRs’ ligands. This filtration allowed not only for the selection of drug-like molecules but also for narrowing down the set to reduce the risk of an inaccurate extrapolation of the QSAR models to the ligands physicochemically dissimilar to the training set. We estimated the remaining compounds’ p*K*_i_ values using the consensus prediction based on our ten QSAR models. We selected potential CB2 ligands with theoretical p*K*_i_ values ≥6.

#### Final Selection

In order to select compounds for the in vitro binding assay, we analyzed in detail the best of the remained 575 candidates for CB2 ligands. We focused on molecules with high docking scores and MM–GBSA binding energy values, especially on those that obtained desired Δ*G*_bind_ from docking to more than one CB2 model. We conducted visual inspection of binding poses and protein–ligand interactions. In some cases we also took the QSAR-predicted p*K*_i_ values into account, although we used these values only auxiliarily in order to lay emphasis on the structure-based part of the study and to maximize the chances of finding structurally new CB2 ligands. We favored compounds with calculated log *P* above 4, based on the properties of most of the potent cannabinoids (Figure S8). We aimed to pick structurally diverse compounds. Among the best-scored ligands, we found multiple known cannabinoids, their metabolites or close derivatives. Such compounds were not considered for the final selection. Lastly, we also evaluated the commercial availability of the most prominent candidates. Finally, we chose 16 structurally diverse compounds for the in vitro binding assay (Table 2, Table S10, Figure S9).

**Table 2:** VS Results for 16 Compounds Selected for the Radioligand Displacement Assay

		5ZTY		6KPC		6PT0			
ID	pharmacophore-fit score	docking score	Δ*G*_bind_ (kcal/mol)	docking score	Δ*G*_bind_ (kcal/mol)	docking score	Δ*G*_bind_ (kcal/mol)	pred. p*K*_i_	pred. p*K*_i_ SD
AS-1	76.1	–10.3	–85.5	–	–	–11.6	–94.9	6.4	0.5
AS-2	64.7	–10.3	–88.3	–9.3	–	–10.2	–92.5	6.1	0.6
AS-3	66.2	–9.1	–	–	–	–10.8	–104.3	6.5	0.5
AS-4	66.3	–9.5	–	–10.5	–69.7	–10.6	–92.2	6.5	0.2
AS-5	65.3	–9.7	–	–	–	–10.6	–93.6	7.3	0.2
AS-6	66.3	–9.6	–	–8.9	–	–10.6	–107.4	6.5	0.1
AS-7	65.5	–10.9	–85.9	–7.2	–	–10.4	–93.0	6.7	0.3
AS-8	66.3	–10.7	–87.5	–7.7	–	–8.2	–	6.3	0.4
AS-9	66.4	–9.5	–	–9.4	–	–10.6	–92.1	6.2	0.4
AS-10	65.6	–10.3	–85.3	–10.5	–81.4	–9.7	–	6.2	0.1
AS-11	64.5	–10.2	–75.6	–11.6	–91.2	–9.5	–	6.4	0.6
AS-12	65.4	–9.7	–	–7.1	–	–10.3	–92.0	6.6	0.3
AS-13	65.7	–10.8	–80.4	–9.0	–	–10.6	–100.3	6.4	0.2
AS-14	65.6	–10.2	–87.8	–9.4	–	–10.0	–	6.1	0.6
AS-15	67.6	–11.7	–85.0	–9.1	–	–12.2	–95.1	6.7	0.1
AS-16	65.7	–9.5	–	–10.4	–17.0	–11.4	–90.6	7.4	0.3

### In Vitro Binding and Functional Assays

**Figure 5:**
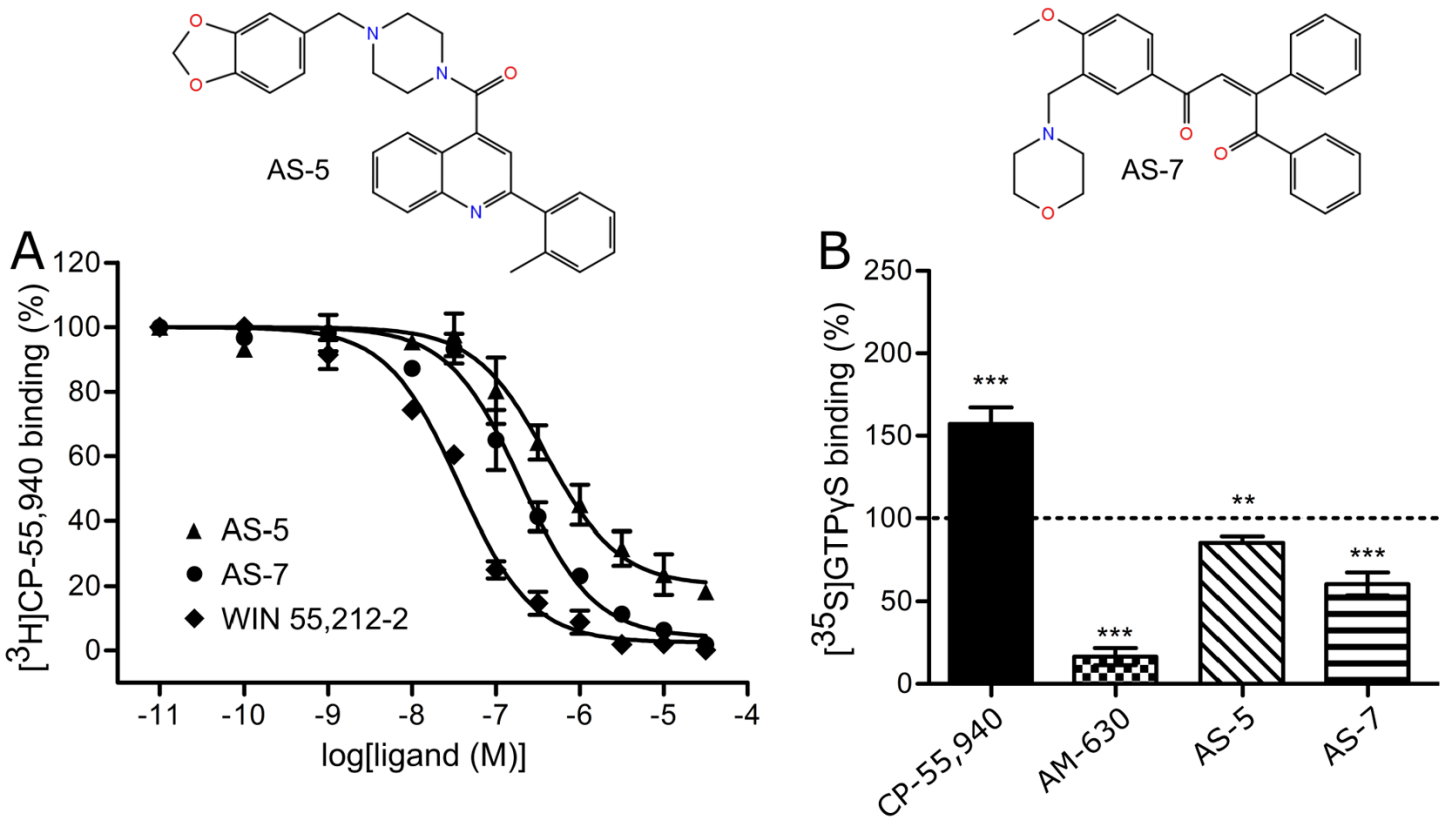
(A) Radioligand displacement curves for two screened compounds with the lowest *K*_i_ values toward human CB2—AS-5 and AS-7. WIN 55,212-2 was issued as the reference compound. Both identified CB2 ligands exhibit desired nanomolar *K*_i_ and structural distinctiveness compared to the other known compounds with high affinity for CB2. (B) Inhibition of CP-55,940-stimulated [^35^S]GTPγS at the CB2 receptor by the compounds at 10 μM. Results were expressed as mean percent of basal [^35^S]GTPγS binding in the presence of 100 nM CP-55,940 as stimulating ligand. AM-630 served as a reference CB2 antagonist. Basal binding was set to 100% and is represented by the dotted line. Data was collected from three separate experiments and analyzed with the two-tailed *t* test. Statistical significance was depicted as follows: **p < 0.01; ***p < 0.001.

CB2 binding affinities of 16 selected compounds were evaluated in the radioligand displacement assay. Four ligands exhibited nanomolar or low micromolar binding affinities toward CB2 (Table 3). The most potent compounds, AS-7 and AS-5 obtained *K*_i_ values of 65 and 210 nM, respectively (Figure 5A). AS-9 and AS-8 showed affinities of 2.1 and 6.37 μM (Figure S10A). AS-8 was tested as a racemic mixture, hence the *K*_i_ of its enantiomer predicted as active is probably lower. The rest of the compounds obtained *K*_i_ values >10 μM or exhibited no binding affinity (Table S11).

**Table 3:** Selected Results of the *K*_i_ Determination with [^3^H]CP-55,940 Displacement Assay

		CB2		CB1	
ID	ZINC ID	p*K*_i_ ± SEM	*K*_i_ (μM, 95% CI)	p*K*_i_ ± SEM	*K*_i_ (μM, 95% CI)
AS-5	ZINC000013893769	6.68 ± 0.10	0.21 (0.13–0.35)	no activity	no activity
AS-7	ZINC000020414208	7.18 ± 0.07	0.065 (0.047–0.090)	5.57 ± 0.9	2.67 (1.7–4.1)
AS-8^a^	ZINC000021727012	5.20 ± 0.09	6.37 (4.1–9.9)	no activity	no activity
AS-9	ZINC000022535815	5.68 ± 0.36	2.1 (0.3–12.2)	no activity	no activity
WIN 55,212-2 (CB2 reference)		8.05 ± 0.06	0.0088 (0.0065–0.012)	–	–
rimonabant (CB1 reference)		–	–	8.22 ± 0.12	0.0059 (0.0035–0.0099)

a Racemic mixture.

Four best compounds were tested for the selectivity in the CB1 binding affinity assay. AS-5, AS-8, and AS-9 exhibited no activity. AS-7 obtained *K*_i_ value = 2670 nM for CB1 (Table 3), which corresponds to a high selectivity index >40 for CB2 (K_i-CB1_/K_i-CB2_). Moreover, CB2 intrinsic activities of these four compounds were evaluated in the [^35^S]GTPγS assay. All identified ligands were shown to act as CB2 antagonists (Figure 5B, Figure S10B).

### Analysis of the Screening Results

In the course of the VS procedure paired with the in vitro verification of compound affinity, we found two potent CB2 ligands from novel chemotypes (Figure 5). Moreover, among the top in silico results we also encountered known cannabinoids, including the already acknowledged CB2 ligands. This is partially an expected result, however, it serves as an additional proof of concept as it confirms that our VS workflow is able to retrieve such compounds, despite combining multiple, diverse techniques and applying various filters and cutoffs.

Among the already known CB2 ligands, our VS techniques placed highly such compounds as WIN 55,212-2, MN-25, and MN-25 2-methyl derivative^84^ (Table 4, Table S12). We also retrieved a few synthetic cannabinoids, including JWH-193, JWH-198, and JWH-200, with high affinity for CB1 that were not evaluated for CB2 affinity.^85^ Finally, among the well-placed compounds we found cannabinoid metabolites and cannabinoid-like compounds, e.g., JWH-203 *N*-(5-hydroxypentyl) metabolite, JWH-250 *N*-pentanoic acid metabolite, or pravadoline.

**Table 4:** Docking and MM–GBSA Results for the Four Most Potent Compounds from the In Vitro Assay and Three Already Known CB2 Ligands for Comparison

	5ZTY		6KPC		6PT0	
ID/name	docking score	Δ*G*_bind_ (kcal/mol)	docking score	Δ*G*_bind_ (kcal/mol)	docking score	Δ*G*_bind_ (kcal/mol)
AS-5	–9.7	–57.9	–	–	–10.6	–93.6
AS-7	–10.9	–85.9	–7.2	–37.5	–10.4	–93.0
AS-8	–10.7	–87.5	–7.7	–61.6	–8.2	–73.3
AS-9	–9.5	–74.5	–9.4	–50.2	–10.6	–92.1
WIN 55.212-2	–11.0	–85.8	–11.7	–71.8	–11.5	–102.8
MN-25	–9.4	–64.4	–8.9	–41.0	–11.0	–97.3
MN-25 2-methyl derivative^a^	–10.3	–85.1	–10.0	–45.1	–10.6	–100.2

a ZINC000013519818.

We analyzed in detail the two compounds with nanomolar affinity—AS-5 and AS-7. Overall, AS-7 exhibited very good docking and MM–GBSA results, while AS-5 was among the compounds with the highest p*K*_i_ values predicted by QSAR (Table 2). This shows the complementarity of both approaches when used together and their inaccuracy if considered separately.

**Figure 6:**
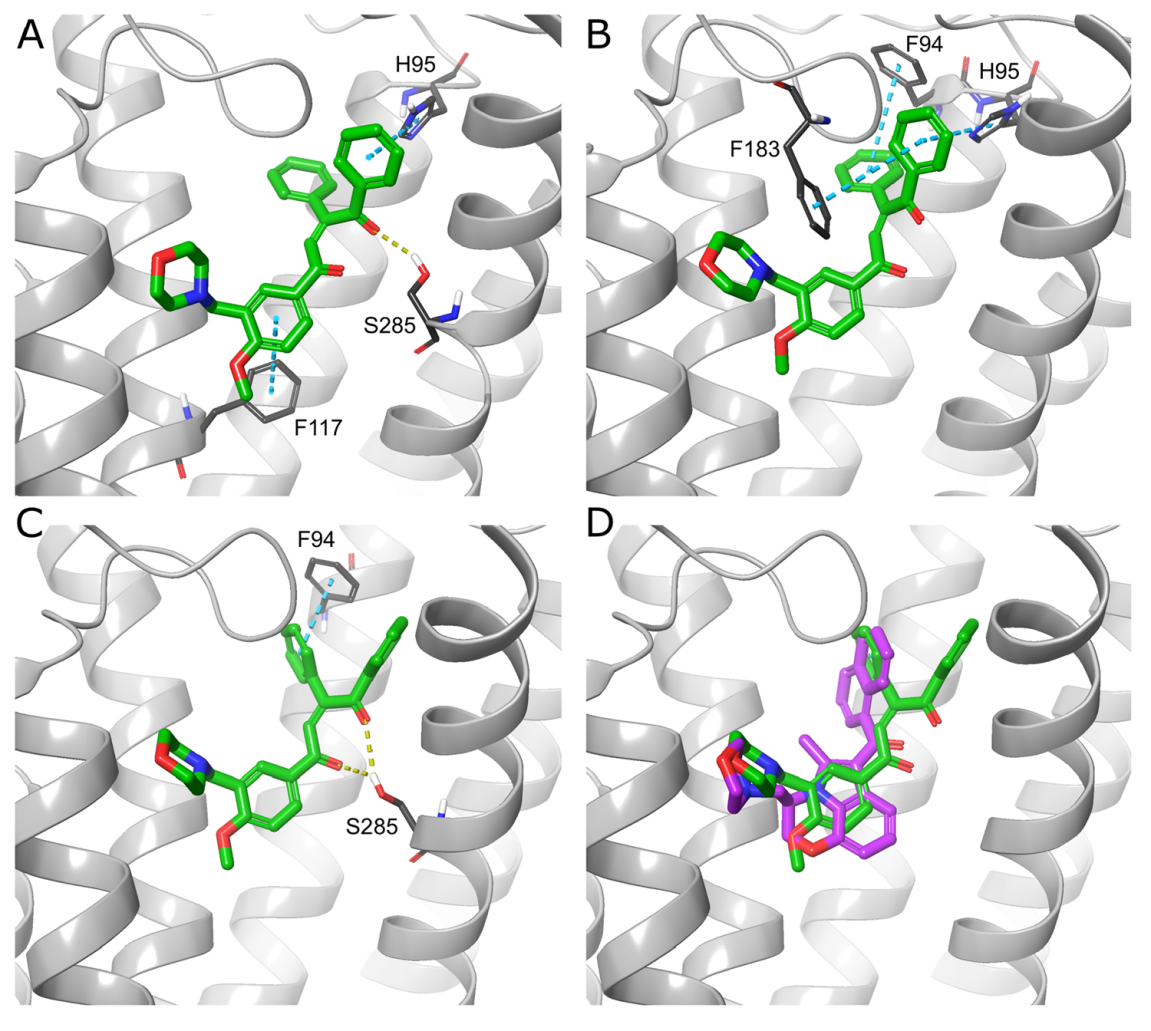
Best identified compound—AS-7 (green) docked to CB2 models based on PDB IDs 5ZTY (A), 6KPC (B) and 6PT0 MD-derived structure (C). Yellow dashed line, H-bond; teal dashed line, π–π interaction. (D) CB2–WIN 55,212-2 (magenta) complex from the largest 6PT0 MD cluster with AS-7 (green) docked to this model. The superposition shows, that despite the different chemotypes, the binding modes of both ligands exhibit similarities, mainly in the placement of the morpholine moieties and carbonyl oxygen atoms and to a lesser extent in the location of two AS-7 benzene rings in similar positions to WIN 55,212-2 central tricyclic moiety and naphthyl group.

AS-7 was docked to all three selected CB2 structures and achieved high docking scores and MM–GBSA Δ*G*_bind_ for two of them, based on PDB ID 5ZTY and the MD-derived 6PT0 model. In all three docking complexes, AS-7 adopts similar binding poses. Depending on the CB2 conformation, the ligand creates various combinations of the following interactions: H-bonds with Ser285, π–π interactions with Phe94, His95, Phe117, and Phe183 (Figure 6A–C). The H-bond with Ser285 occurred in two output complexes (with 5ZTY and 6PT0 CB2 models), and according to the docking results, it is possible that the interaction may be formed with either of the ligand’s carbonyl oxygen atoms. This H-bond was not present in the complex with 6KPC (Figure 6B), which is one of the main reasons for significantly worse Δ*G*_bind_ than for the two other models (Table 4). Moreover, AS-7 exhibits some structural similarities to WIN 55,212-2, namely the occurrence of a morpholine moiety in both compounds. AS-7 docked to 6PT0 model adopted a pose analogical to WIN 55,212-2 with the same placement of the morpholine rings. Additionally, there are also similarities in the localization of WIN 55,212-2 and one of the AS-7 carbonyl oxygen atoms. Also, two of the AS-7 benzene rings are placed in nearly the same positions compared to WIN 55,212-2 central tricyclic moiety and the distal ring of the naphthyl group (Figure 6D). All these similarities suggest that the putative binding mode of AS-7 is probably predicted properly.

**Figure 7:**
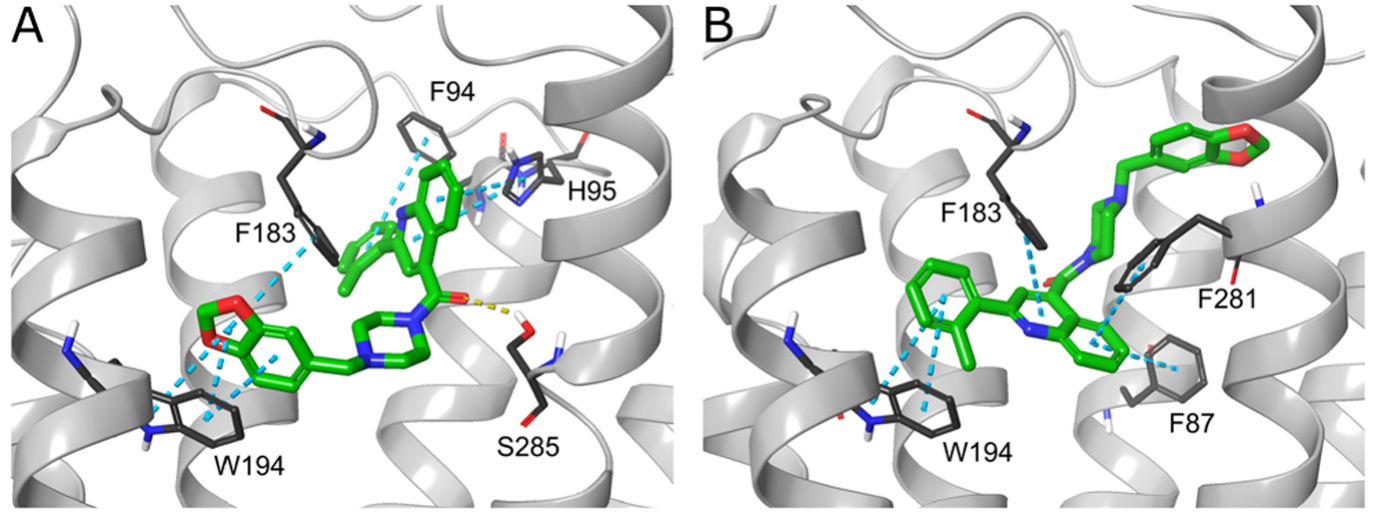
AS-5 (green) docked to CB2 models based on PDB IDs 5ZTY (A) and 6PT0 MD-derived structure (B). Yellow dashed line, H-bond; teal dashed line, π–π interaction.

AS-5 was able to fit into the two CB2 models: 5ZTY and 6PT0. However, substantially different binding poses were predicted for each of them (Figure 7), indicating that at least one pose is false. AS-5 is structurally diverse from all ligands cocrystallized with CB1 or CB2; thus, there is no certain way to state which binding mode is the correct one. Based on the functional activity of AS-5, the pose bound to the inactive CB2 model (5ZTY) has a higher chance of being properly predicted. In complex with 5ZTY, AS-5 forms an H-bond with Ser285 as well as several π–π interactions with Phe94, His95, Phe183, and Trp194 (Figure 7A). Regardless of the ambiguous docking results, AS-5 achieved one of the best QSAR-predicted p*K*_i_ values (7.3).

AS-5 and AS-7 possess similar calculated physicochemical properties, which are also in agreement with ranges observed for the majority of potent CB2 ligands (Figure S8). Both compounds have the log *P* > 4, no H-bond donors and low PSA < 70 Å^2^ (Table S10).

It is important to state that AS-7 possesses an α,β-unsaturated ketone moiety. This structural feature comes with a risk of high reactivity and toxicity.^86,87^ Interestingly, AS-7 was not flagged by the PAINS (pan assay interfering compounds) filter in Schrödinger Canvas. Nevertheless, because of the potential toxicity, AS-7 is not a perfect candidate for a lead compound itself. However, the C=C double bond is probably not important for CB2 binding. If one would consider working on this ligand or chemotype further, we would advise trying a saturated version of AS-7 for the hit-to-lead stage. We performed additional calculations for the modified compound, AS-7-1 (Figure S11A), including docking to three CB2 models used in the VS procedure. AS-7-1 obtained nearly the same putative binding modes as AS-7 (Figure S11C,D) and achieved similar docking scores, MM–GBSA binding free energies and the p*K*_i_ value predicted with QSAR (Table S13).

Two other compounds—AS-8 and AS-9 exhibited low micromolar affinity toward CB2 (Table 3). Both ligands were docked to all three CB2 structures used. However, each compound showed two putative binding modes (Figure S12). Notably, in both instances the predicted binding mode was the same in complexes with PDB ID 6KPC and 6PT0 MD model, while different for 5ZTY. This division is related to docking to different CB2 activation states, which is not always observed in silico, as shown by the results of the cross-docking performed during validation (Table S5). Taking into account the functional activity of both ligands, we presume that the poses predicted for 5ZTY are more likely to be correct. The examples of AS-5, AS-8, and AS-9 illustrate the difficulties of proper pose prediction while docking to highly hydrophobic binding sites, such as in CB2.

### The Potential and Limitations of Rational In Silico Optimization

Apart from the hit identification, methods we employed in this VS may also be utilized in rational computer-aided hit-to-lead stage or lead optimization. However, these techniques should be used with caution, bearing in mind their limited accuracy.

SB methods allow for rational structure modification based on the putative binding mode of the hit compound. In the case of highly hydrophobic GPCRs such as CB2, proper pose prediction is a nontrivial task, because of the abundance of Phe and other residues with aromatic side chains at the binding site along with usually multiple aromatic rings of CBRs’ ligands. This in turn leads to multiple π–π interactions, which tend to be nonspecific; thus, docking algorithms may propose different poses for a single compound combined with specific protein conformations (Figure 7). We also encountered this problem during validation of docking pose prediction (Table S5). Thus, rational SB optimization can only be employed for ligands with well-predicted binding modes. In this case, it is a valid strategy for AS-7 but could be inadequate for AS-5.

On the other hand, LB methods may also be useful tools for optimization. Pharmacophore screening conducted at the beginning of our VS procedure was an important element of the study. Nonetheless, this technique is suitable for hit identification. By contrast, the machine learning QSAR models might represent a valuable addition to the optimization process. However, this method should be used with caution as well. First, p*K*_i_ values predicted by QSAR have limited accuracy and possess a significant standard deviation. In the case of our models for CB2 ligands it was 0.46–0.79 p*K*_i_ units (Table S9), which is a considerable amount, especially for optimization. Second, apart from the occurrence of false positives, for some ligands the QSAR-predicted p*K*_i_ values are inferior to values produced by the in vitro analyses, e.g., for AS-7. Nevertheless, such QSAR models may be a valuable additional estimate of binding affinity, especially when combined with other methods. They could be particularly useful for compounds that are not suitable for SB optimization, such as AS-5. Finally, during the in silico-augmented optimization, the basic physicochemical properties prediction may prove exceptionally useful for such specific, highly hydrophobic ligands as cannabinoids.

### Insights from and Possible Alterations in the VS Workflow

The aim of this study was primarily to find novel CB2 ligands, but also to test the proposed VS procedure. Indeed, based on the screening and on the in vitro binding results, we came to valuable conclusions and ideas for possible modifications of the procedure.

An important finding derived from the docking validation suggests that Glide SP is probably more suitable for CB2 than Glide XP (extra precision). In the case of a limited number of receptor structures, docking to MD-derived structures could be a valid strategy. However, in our study, most of the CB2 conformations from MD proved to be similar or inferior versions of PDB structures. We decided to dock to only one CB2 model from the MD, based on the trajectory of PDB ID 6PT0, as it performed slightly better in our validation. It may be worth to explore more CB2 conformations, although they should be introduced into VS with caution.

In all PDB-deposited CB2 structures, the hydroxyl group of Ser285 either forms an H-bond with the carbonyl or hydroxyl group of the ligand or is localized in such proximity that the interaction may be formed in a conformational landscape of the flexible receptor. Similarly, the H-bond with Ser285 is crucial for AS-7 and for one of the putative binding modes of AS-5. Thus, it may be a potentially rewarding strategy to conduct docking with constraints for the H-bond with Ser285 hydroxyl group for PDB IDs 5ZTY and 6PT0.

Herein, for the physicochemical properties filtration, we used Lipinski’s and Veber’s rules with an additional cutoff for log *P*. However, there are also other valid approaches to tackle this issue. Some of the cutoffs can be altered if one does not want to obtain hit compounds that fulfill drug-like criteria, but rather prefers the latter optimization of the parameters. For CB2, the most important properties to consider include log *P*, PSA, the number of H-bond donors, and the number of rotatable bonds. As CBRs’ ligands are highly hydrophobic, the log *P* values do not obey Lipinski’s rule, usually ranging from 4 to 7.5 (Figure S8). On the other hand, the criteria for PSA may be set even more strictly than in Veber’s rule—e.g. to ≤100 Å^2^ or even ≤80 Å^2^ (Figure S8). Similarly, the number of H-bond donors does not exceed 3, with an average of 0.6 for the CB2 ligands deposited in ChEMBL with *K*_i_ ≤ 100 nM (Table S14). Lastly, some of the CBRs’ ligands, mainly endocannabinoid-like compounds, possess more than 10 rotatable bonds (in some cases even more than 20).

We employed the QSAR models mainly auxilarily. However, based on our in vitro verification, they proved to perform reasonably well and their contribution for the selection of potential hits could be increased. Thus, it may be effective to change the cutoff for the predicted p*K*_i_ to around 6.5. Also, utilization of different combinations of specific QSAR models might be considered (Table S12).

Herein, we conducted the QSAR after docking and MM–GBSA calculations to obtain more information on the VS procedure’s performance. In order to minimize the time required for the VS campaign, physicochemical properties filtration and QSAR should precede docking. In this study, the reversed order was established mainly to gain more insight in the effectiveness of SB methods for such specific, hydrophobic receptor as CB2, without previous QSAR filtering. Docking and MM–GBSA proved to be effective, being able to significantly narrow-down the number of potential ligands. Nevertheless, the procedure still needed an additional filter, which should be based on a LB method. This shows the complementarity of both approaches. For similar VS campaigns, conducting QSAR before docking would be advised.

## Conclusions

CB2 is a very promising molecular target for a plethora of possible therapeutic applications. Despite multiple well-known CB2 ligands, a large amount of them is comprised in only a few chemotypes. Because of the complexity of CBRs and ECS, there is a need to explore other, structurally diverse CB2 ligands. However, to date, the rational, SB design of such compounds is not a widespread approach. In this study, we established a VS workflow and conducted subsequent screening based on SB methods augmented with LB techniques, which we verified in vitro. We showed that the proper combination and utilization of widely accessible, computational tools is effective even for such a problematic molecular target with a highly hydrophobic binding site. The VS procedure established here may be employed not only for the identification of new CB2 ligands, but also for designing compounds acting via other, similar proteins.

Based on our in silico tests and on in vitro verification of the screening results, we provide insight into the potential and limitations of the computational procedure. Combination of docking, MM–GBSA calculations, pharmacophore screening, and QSAR proved to be effective. Despite the simplicity, computation of physicochemical properties is of significant importance for such a specific group of hydrophobic ligands. Focusing on docking and MM–GBSA during the selection of candidates for the in vitro binding assay allowed us to find CB2 ligands from new chemotypes. Because SB methods were the central focus of this study, a few conclusions regarding docking should be emphasized. Glide SP is a preferable choice for CB2. Difficulty in pose prediction is a considerable limitation. Utilization of multiple CB2 conformations is necessary due to the substantial induced-fit effect. The arrangement of Ser285 hydroxyl group is one of the crucial factors as it is the most important H-bond donor for various chemotypes.

In summary, using the workflow established here, we identified two novel, selective CB2 antagonists—AS-5 and AS-7, with *K*_i_ values of 210 and 65 nM, respectively. Both compounds belong to new chemotypes. AS-7 exhibits limited structural and binding mode similarities to WIN 55,212-2. These novel CB2 ligands, especially AS-7, provide a promising starting point for future optimization and for development of drug candidates acting via CB2.

## Data Availability

The input data used to conduct the study were obtained from freely available databases. CB2 structures were downloaded from PDB (https://www.rcsb.org/). Structures of the compounds used for pharmacophores’ and docking validation come from PubChem (https://pubchem.ncbi.nlm.nih.gov/) and free Schrödinger decoy set (https://www.schrodinger.com/products/glide/). Training and test compounds’ structures for QSAR models were obtained from ChEMBL (https://www.ebi.ac.uk/chembl/). Screening compounds structures were downloaded from ZINC (https://zinc.docking.org/). Human CB2 sequence used for building CB2 models for MD was retrieved from UniProt (https://www.uniprot.org/). A free for academic use Web server—CHARMM-GUI (https://charmm-gui.org/) was used for generation of systems for MD. MD was conducted in free software—GROMACS 2018.8 (https://www.gromacs.org/). The main part of the VS requires several commercially available programs. Pharmacophore screening was performed using LigandScout 4.4.4 (http://www.inteligand.com/). Schrödinger Maestro 2017–1 (https://www.schrodinger.com/products/maestro/) was employed for docking, MM–GBSA calculations, and QSAR. The analysis of the in vitro experiment was conducted with GraphPad Prism 5.0 software (https://www.graphpad.com/scientific-software/prism/). BIOVIA Discovery Studio v20.1.0.19295 (https://www.3ds.com/products-services/biovia/products/molecular-modeling-simulation/biovia-discovery-studio/) was used to prepare CB2 structures for MD. The most crucial data were deposited on GitHub (https://github.com/ilbsm/Identification_of_CB2_ligands).

## Supporting Information Available

Additional MD details (Methods); tables with CB2 ligands used as test compounds for pharmacophore, docking, and MM–GBSA validation; selected results, curves, and plots obtained during the validation; RMSD plots from MD simulations and replicas; thresholds used for specific stages of the virtual screening procedure; statistical parameters and results of QSAR tests; computed physicochemical properties (table and histograms) of CB2 ligands; structural formulas and calculated physicochemical properties of the compounds selected for the in vitro assay; results of the in vitro binding and functional assays; and selected results of the virtual screening procedure or additional computations (QSAR predictions, docking scores, putative binding modes) (PDF)
Information regarding purity and identity of the compounds selected for the in vitro binding assay, with chromatograms and spectra provided by the suppliers (ZIP)
SMILES of the 16 compounds selected for the in vitro binding assay(XLSX)

## Abbreviations Used

[^35^S]GTPγS: [^35^S]guanosine 5′-[γ-thio]triphosphate
ADME: absorption, distribution, metabolism, excretion
CB1: cannabinoid receptor type 1
CB2: cannabinoid receptor type 2
CBR: cannabinoid receptor
CgenFF: CHARMM General Force Field
CI: confidence interval
CNS: central nervous system
cryo-EM: cryoelectron microscopy
CXCR4: C-X-C chemokine receptor type 4
ECL2: extracellular loop 2
ECS: endocannabinoid system
GPCR: G-protein-coupled receptor
GPR55: G-protein-coupled receptor 55
ICL3: intracellular loop 3
*K*_i_: inhibition constant
LB: ligand-based
LBDD: ligand-based drug design
MAGL: monoacylglycerol lipase
MD: molecular dynamics
MM–GBSA: molecular mechanics–generalized Born surface area
MW: molecular weight
PAINS: pan assay interfering compounds
PDB: Protein Data Bank
POPC: 1-palmitoyl-2-oleoylphosphatidylcholine
PSA: polar surface area
QSAR: quantitative structure–activity relationship
RMSD: root-mean-square deviation
SB: structure-based
SBDD: structure-based drug design
SD: standard deviation
SEM: standard error of measurement
SP: standard precision
TM: transmembrane (helix)
TRPV1: transient receptor potential vanilloid type 1 (channel)
VS: virtual screening
XP: extra precision
XRD: X-ray diffraction
