# Supplementary material for: Identification of Novel CB2 Ligands through Virtual Screening and In Vitro Evaluation
Source: J Chem Inf Model. 2023 Jan 24;63(3):1012–27. doi: 10.1021/acs.jcim.2c01503 (PMC9930120; doi:10.1021/acs.jcim.2c01503)
Supplement: Supplementary file 1 — ci2c01503_si_001.pdf [file ci2c01503_si_001.pdf]

Supporting Information: Identification of Novel CB2 Ligands through Virtual Screening and In Vitro Evaluation

Adam Stasiulewicz,[†,‡] Anna Lesniak,[¶] Magdalena Bujalska-Zadrożny,[¶] Tomasz Pawiński,[†] and Joanna I. Sulkowska[*,‡]

†*Department of Drug Chemistry, Faculty of Pharmacy, Medical University of Warsaw, Banacha 1, 02-097 Warsaw, Poland*

‡*Centre of New Technologies, University of Warsaw, Banacha 2c, 02-097 Warsaw, Poland*

¶*Department of Pharmacodynamics, Faculty of Pharmacy, Medical University of Warsaw, Banacha 1b, 02-097 Warsaw, Poland*

E-mail: j.sulkowska@cent.uw.edu.pl

Methods

Molecular Dynamics

The substitutions from the crystal structures were reverted to the wild-type version during the initial protein preparation. The reverted point mutations included: Gly78Leu, Thr127Ala, Thr153Leu, Arg242Glu, Gly304Glu for PDB ID: 5ZTY and Gly78Leu, Thr127Ala, Thr153Leu, Gly210Ala, Arg242Glu, Gly304Glu for PDB ID: 6KPC.

The MD simulations were conducted in GROMACS 2018.8.[1] Firstly, we performed steepest-descent energy minimization with 10,000 steps. Then, we conducted six phases of equilibration with different parameters and variable constraints. Steps 1 and 2 included NVT ensemble with Berendsen thermostat, $t = 1$ ns, and $\Delta t = 0.001$ ps. Temperature was set to 310.15 K. Step 3 consisted of NPT ensemble with Berendsen thermostat, Berendsen pressure coupling, $t = 5$ ns, and $\Delta t = 0.001$ ps. Steps 4–6 consisted of NPT ensembles with Berendsen thermostat, Berendsen pressure coupling, $t = 5$ ns, and $\Delta t = 0.002$ ps. MD production was conducted using Nose–Hoover thermostat, Parrinello–Rahman pressure coupling, $t = 1$ μs, and $\Delta t = 0.002$ ps.

Obtained trajectories were clustered using gmx cluster with gromos algorithm.[2] Clustering was based on heavy atoms of amino acids within 5 Å of ligands in PDB IDs: 5ZTY, 6KPC and 6PT0. The specific amino acid numbers: 25, 86, 87, 90, 91, 94, 95, 106, 109–111, 113, 114, 117, 164, 165, 168, 180–184, 186, 190, 191, 194, 195, 198, 258, 261, 262, 265, 281, 282, 285, 288.

Additionally, we conduced replica simulations for all five original MD production runs. We utilized the same starting points for both original runs and replicas (independent runs beginning with the state derived from a single equilibration process). We analyzed the replica runs similarly to the original simulations. We calculated root-mean-square deviation (RMSD) of CB2 C_α atoms as well as RMSD of heavy atoms of the ligands in the case of three CB2–ligand simulations. Moreover, for both original runs and replicas, we calculated

RMSD of the heavy atoms of selected binding site residues (amino acids specified above). All RMSD calculations were performed after superposition on CB2 C_α atoms. We compared average RMSD values for the original and replica simulations in Table S4 and showed the similarities of the trajectories using RMSD vs time plots (Figures S3–S5).

The original and replica production runs exhibited only expected, slight differences across the trajectories. Importantly, the small differences were visible mainly in C_α RMSD (due to peripheral, flexible regions of CB2, e.g. intracellular loop 3), whereas the trajectories exhibited very high similarities in binding site residue RMSD or ligand RMSD. This proves the significance of the original MD simulations and their suitability for the clustering performed to obtain a larger number of CB2 binding site conformations for the docking validation.

Table S1: CB2 ligands with K_i <100 nM toward human CB2 used for pharmacophores' validation.

Name/ID	Structural formula	K_i (nM)	Reference
AM-10257	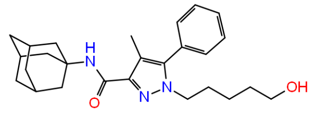	0.08	[3]
AM-12033	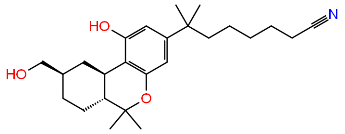	0.37	[4]
AM-1241	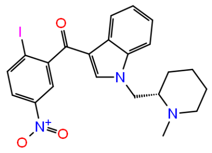	7.1	[5]
AM-630	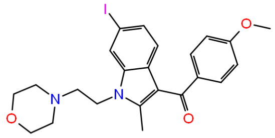	31.2	[6]
AM-841	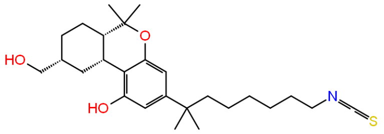	1.51	[7]
BAY 59-3074	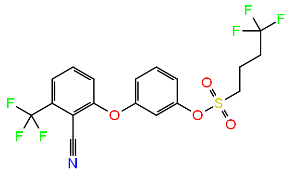	45.5	[8]
Cannabinol	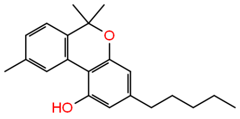	96.3	[9]
CP-55,940	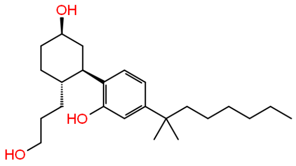	0.79	[10]
GW-405,833	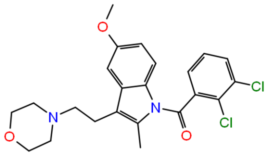	3.92	[11]
HU-210	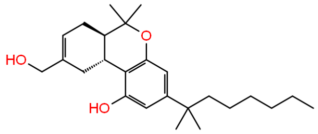	0.22	[5]

HU-308	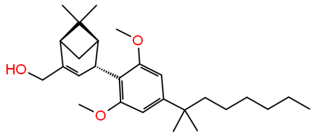	22.7	5
JWH-015	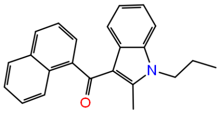	35	12
JWH-133	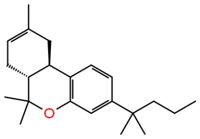	3.4	13
JWH-151	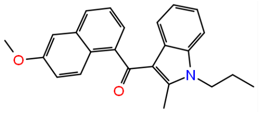	30	14
MDMB-Fubinaca	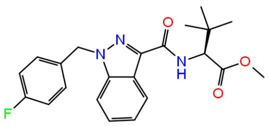	0.1228	15
Nabilone	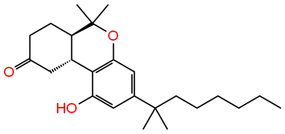	17.6	16
SR-144,528	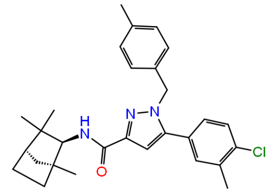	5.6	6
WIN 55,212-2	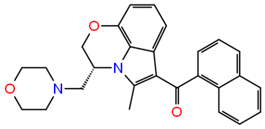	5.36	17
Δ-8-THC	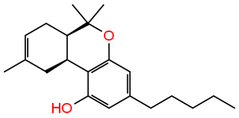	25	18
Δ-9-THC	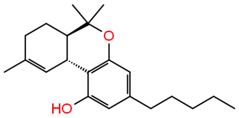	32.2	19

Table S2: Selected best pharmacophores.

**PDB IDs**	**Superposition**	**Feature combination**	**Max. no. of omitted descriptors**	$\mathbf{EF_{1\%}}$	$\mathbf{EF_{5\%}}$	**True positives**	**Hits**
6KPC	–	–	5	15.3	5	13	326
6KPC	–	–	6	15.3	5	18	715
6PT0	–	–	2	30.6	6	10	346
6PT0	–	–	3	30.6	6	19	810
6KPC, 6PT0	features	shared	0	15.3	4	19	598
6KPC, 6PT0	ref. points	merged	9	25.5	9	15	339
6KPC, 6KPF	features	shared	0	25.5	10	15	386
6KPC, 6KPF	ref. points	shared	0	25.5	9	14	329
5ZTY, 6KPC, 6PT0	ref. points	merged	14	25.5	7	15	313
5ZTY, 6KPC, 6PT0	ref. points	merged	15	25.5	7	16	501
6KPC, 6KPF, 6PT0	ref. points	merged	10	20.4	10	13	130
6KPC, 6KPF, 6PT0	ref. points	merged	11	20.4	10	17	604
5ZTY, 6KPC, 6KPF, 6PT0	ref. points	merged	14	25.5	8	11	328
5ZTY, 6KPC, 6KPF, 6PT0	ref. points	merged	15	25.5	8	13	513

Table S3: CB2 ligands with K_i <100 nM toward human CB2 used for pharmacophores' confirmatory validation.

Name/ID	Structural formula	K_i (nM)	Reference
2	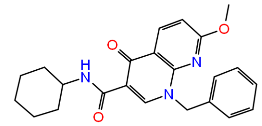	11	20
4g	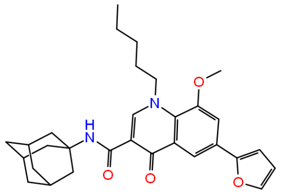	8.5	21
13	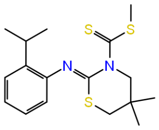	9	22
22e	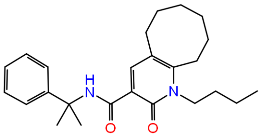	0.1	23
24	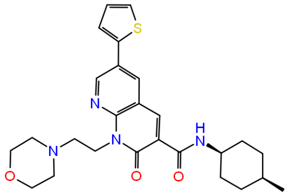	0.17	24
40	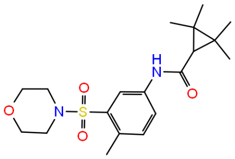	23	25
52	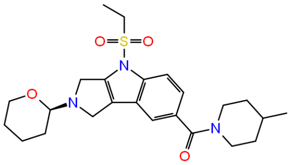	17.6	26
AM-10257	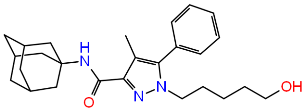	0.08	3
AM-12033	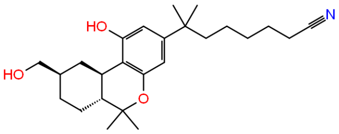	0.37	4
AM-1241	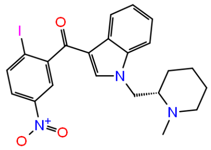	7.1	5

BAY-59-3074	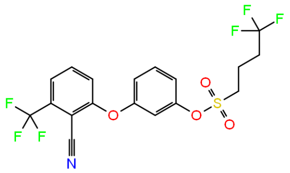	45.5	8
CP-55,940	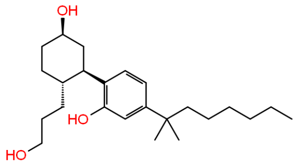	0.79	10
GW-405,833	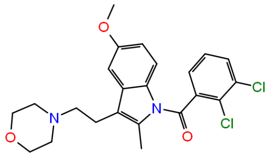	3.92	11
JWH-015	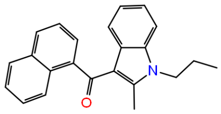	35	12
MDMB-Fubinaca	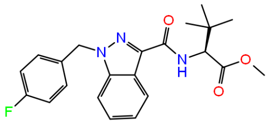	0.1228	15
S-777469	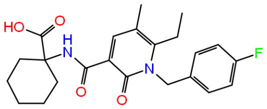	36	27
Sch35966	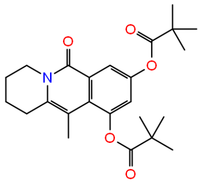	6.8	28
SR-144,528	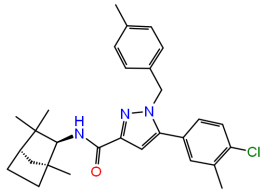	5.6	6
WIN 55,212-2	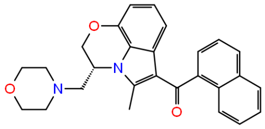	5.36	17
Δ-9-THC	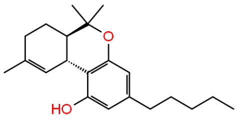	32.2	19

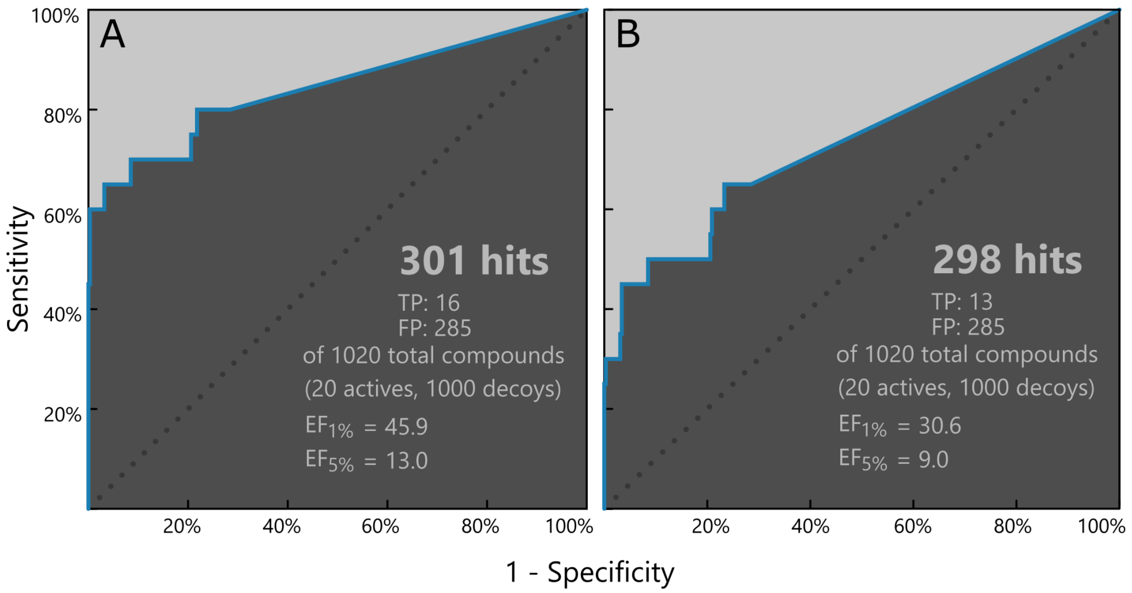

Figure S1: Receiver operating characteristic curve from the validation of the pharmacophore used for screening. (A) Validation performed using standard active test set (Table S1). (B) Confirmatory validation with modified active set (Table S3).

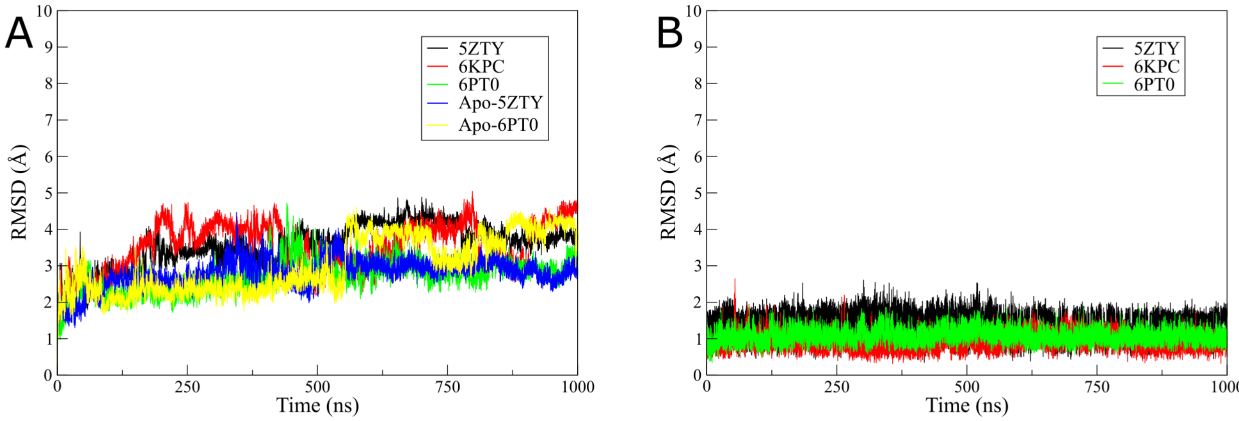

Figure S2: RMSD plots of protein C_α atoms (A) and ligands' heavy atoms (B) from MD simulations. RMSD computed after superposition on C_α atoms.

Table S4: Comparison of average RMSD (Å) ± SD for the original MD runs and replicas. RMSD computed after superposition on protein C_α atoms.

**PDB ID**	**Protein (C_α atoms)**		**Binding site (heavy atoms)**		**Ligand (heavy atoms)**	
	Original run	Replica	Original run	Replica	Original run	Replica
**5ZTY**	3.56 ± 0.58	2.91 ± 0.32	1.38 ± 0.12	1.75 ± 0.16	1.43 ± 0.37	1.50 ± 0.35
**6KPC**	3.54 ± 0.66	4.50 ± 0.66	1.36 ± 0.17	1.53 ± 0.24	0.91 ± 0.22	1.00 ± 0.23
**6PT0**	2.67 ± 0.46	2.82 ± 0.67	1.47 ± 0.18	1.46 ± 0.17	1.07 ± 0.20	1.07 ± 0.26
**Apo-5ZTY**	2.80 ± 0.39	3.76 ± 0.39	2.61 ± 0.47	2.77 ± 0.29	–	–
**Apo-6PT0**	3.01 ± 0.78	3.13 ± 0.86	1.89 ± 0.20	2.26 ± 0.46	–	–

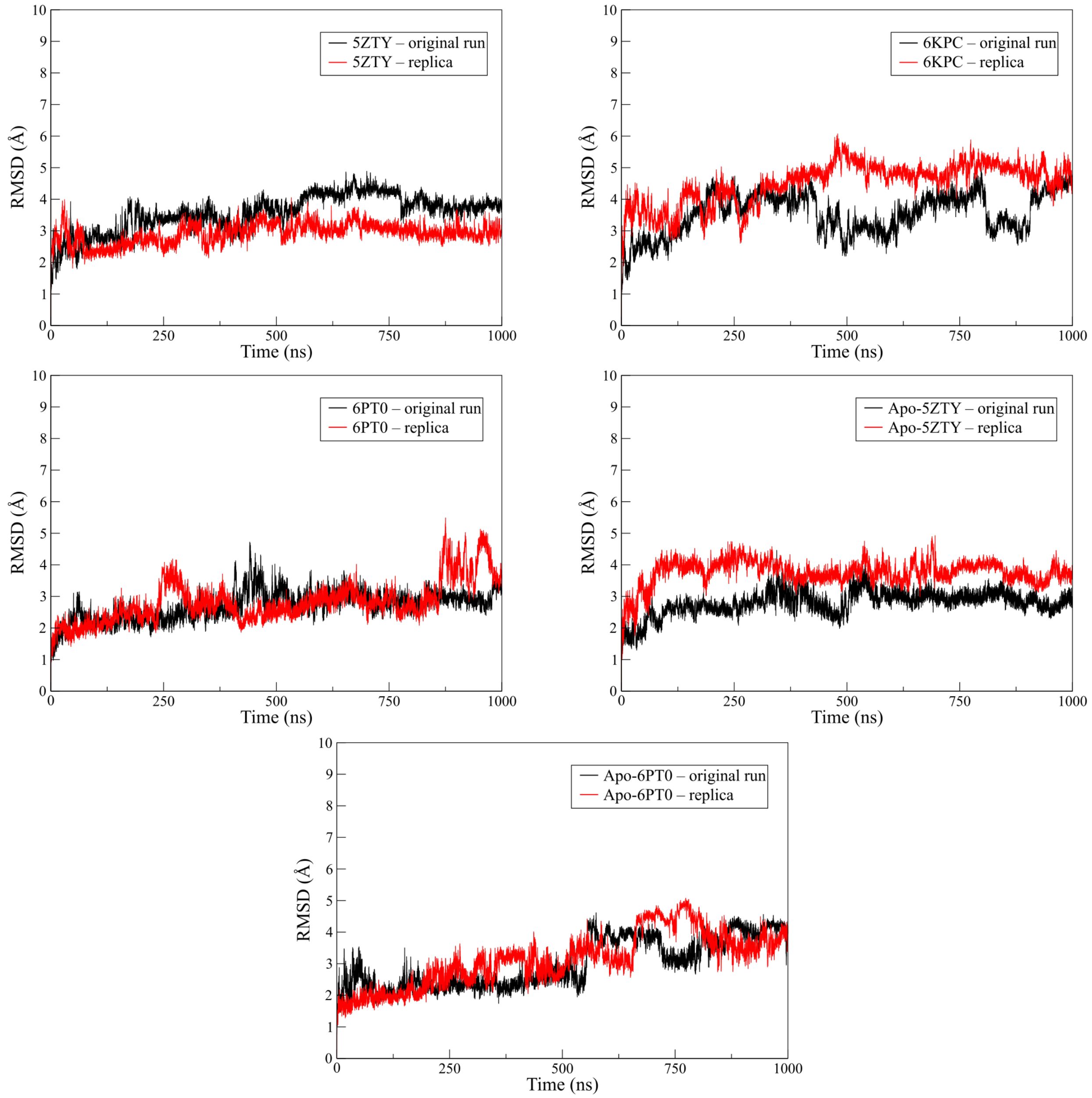

Figure S3: RMSD plots of protein C_α atoms from the original MD simulations and from replicas. RMSD computed after superposition on C_α atoms.

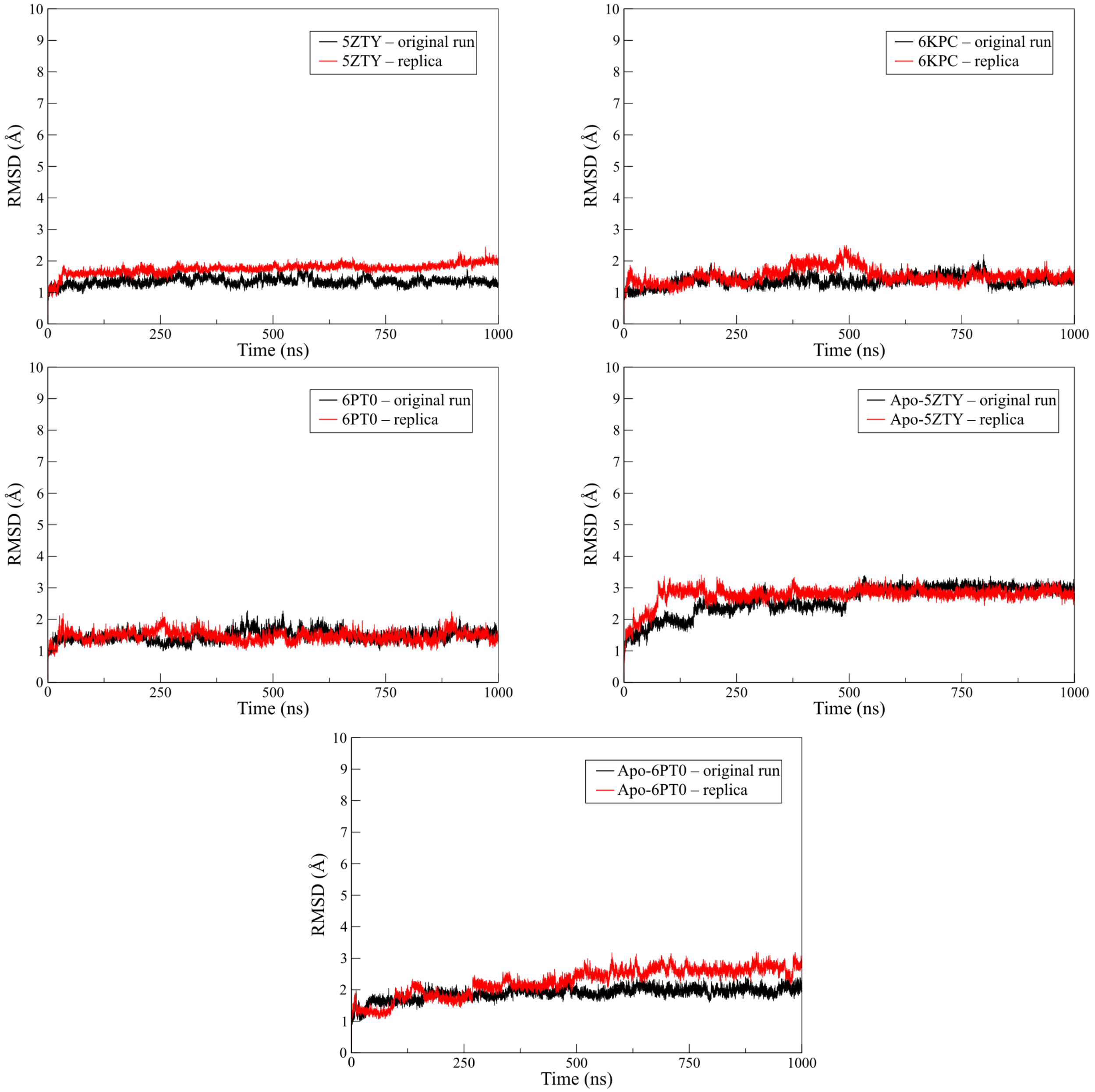

Figure S4: RMSD plots of heavy atoms of the selected binding site residues from the original MD simulations and from replicas. RMSD computed after superposition on C_α atoms.

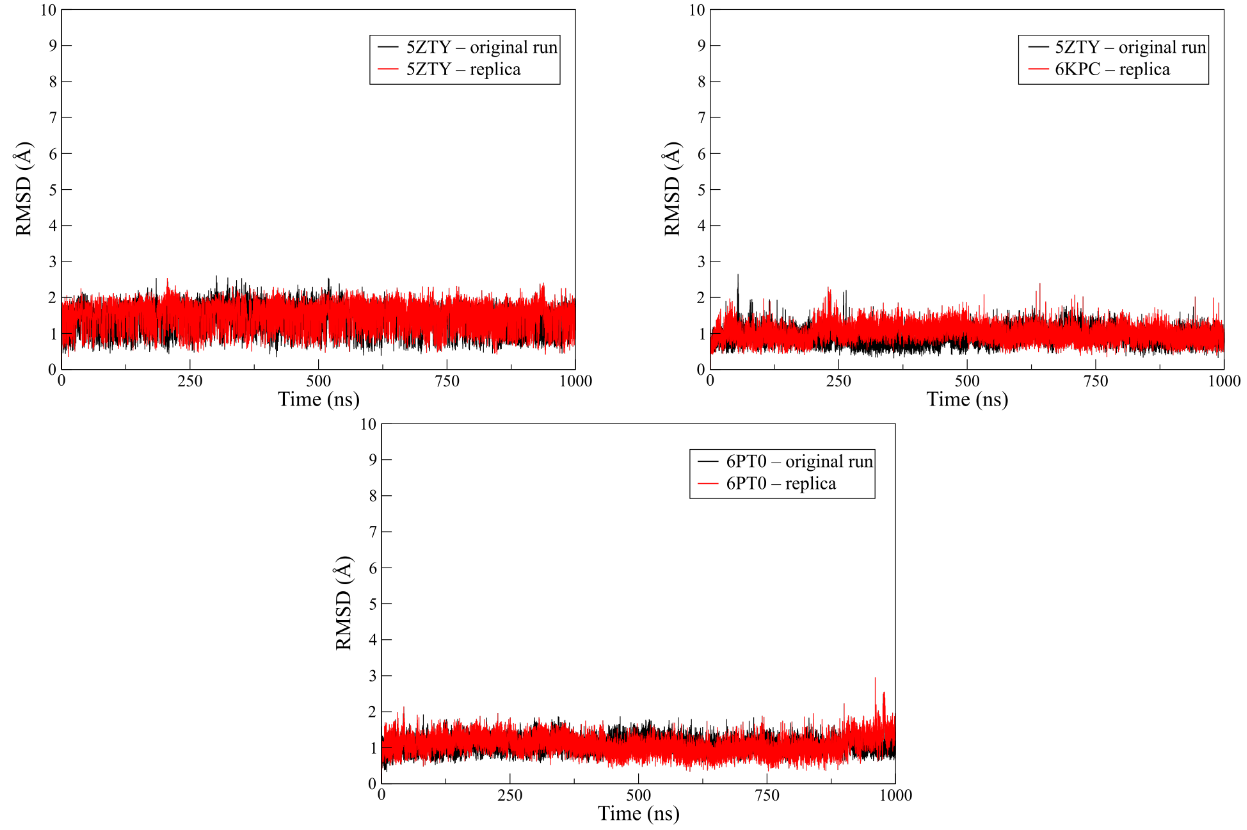

Figure S5: RMSD plots of ligand heavy atoms from the original MD simulations and from replicas. RMSD computed after superposition on C_α atoms.

Table S5: RMSD (Å) of ligands' heavy atoms from cross-docking.

Ligand PDB ID \ Protein PDB ID	**5ZTY**	**6KPC**	**6KPF**	**6PT0**
**5ZTY**	1.49	8.52	8.29	3.17
**6KPC**	1.04	0.69	1.42	8.66
**6KPF**	1.57	1.03	0.97	8.48
**6PT0**	2.89	3.19	7.56	2.00

Table S6: CB2 ligands with different K_i values used as test compounds in docking and MM–GBSA validation.

Name/ID	Structural formula	K_i (nM)	Reference
2-AG	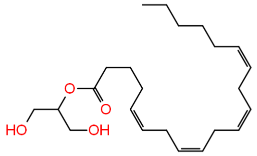	1400	[29]
Ajulemic acid	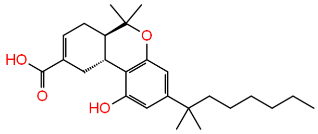	170.5	[13]
AM-10257	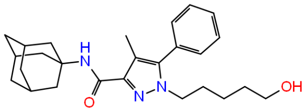	0.08	[3]
AM-12033	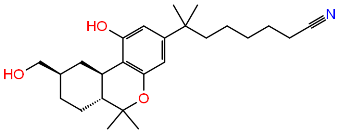	0.37	[4]
AM-1241	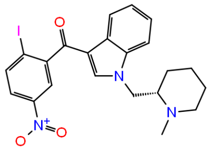	7.1	[5]
AM-2233	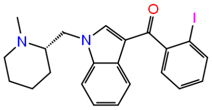	9.2	[7]
AM-251	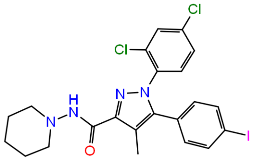	110	[30]
AM-281	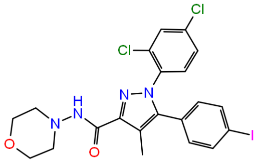	4870	[17]
AM-4056 (HU-243)	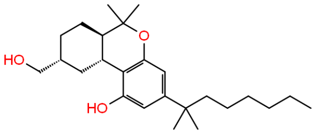	2.14	[7]
AM-630	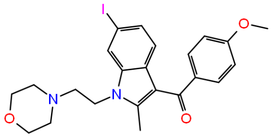	31.2	[6]
AM-7528	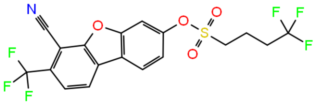	317	[8]

AM-841	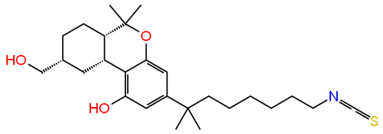	1.51	7
Anandamide	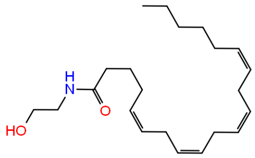	180	31
BAY 59-3074	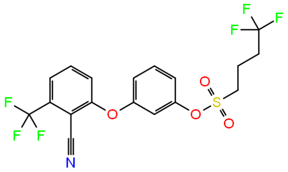	45.5	8
Cannabichromanone B	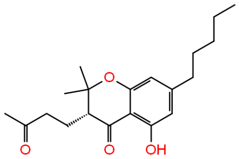	4371	32
Cannabidiol	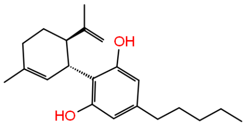	4582	32
Cannabidivarin	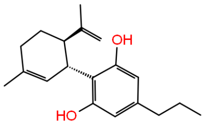	3970	32
Cannabigerol	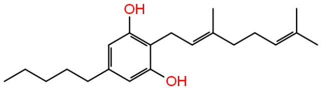	2919	32
Cannabinol	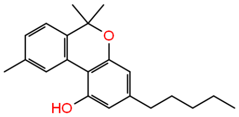	96.3	9
Cannabiripsol	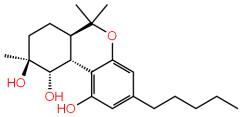	2143	32
CP-55,940	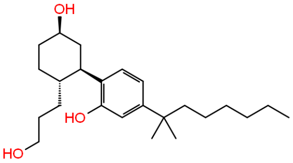	0.79	10
GW-405,833	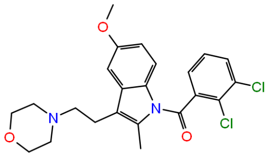	3.92	11
HU-210	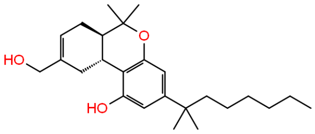	0.22	5

Ibipinabant (SLV319)	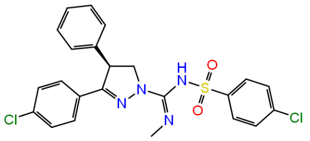	7943	33
JWH-015	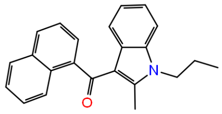	35	12
JWH-133	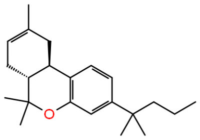	3.4	13
JWH-213	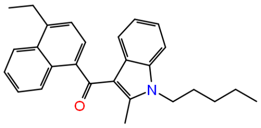	0.42	34
JWH-241	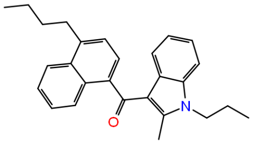	49	34
L-759,633	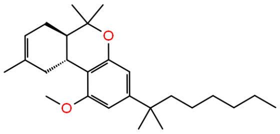	6.4	6
MDMB-Fubinaca	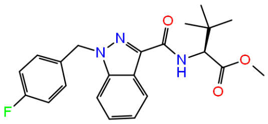	0.1228	15
Methanandamide	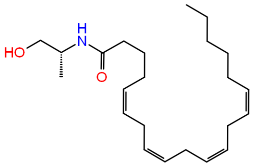	220	35
Nabilone	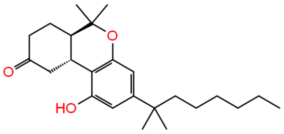	17.6	16
NNEI	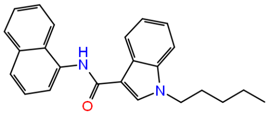	45.29	15
Rimonabant	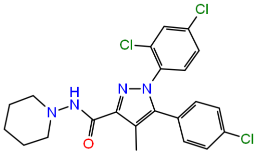	313	36
SR-144,528	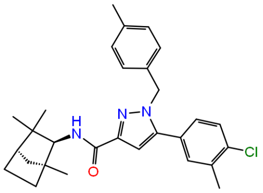	5.6	6

Surinabant	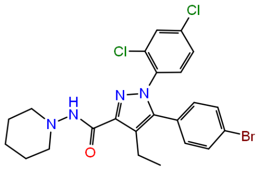	442	[37]
WIN 55,212-2	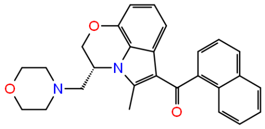	5.36	[17]
Δ-8-THC	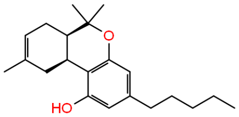	25	[18]
Δ-9-tetrahydrocannabinolic acid	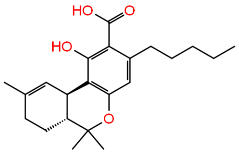	1650	[32]
Δ-9-THC	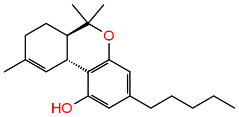	32.2	[19]

Table S7: CB2 ligands with different K_i values used as test compounds in docking and MM–GBSA confirmatory validation.

Name/ID	Structural formula	K_i (nM)	Reference
2	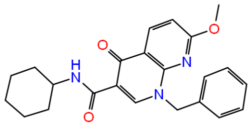	11	[20]
4g	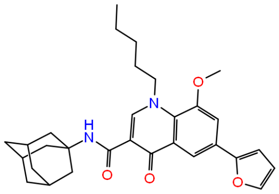	8.5	[21]
10a	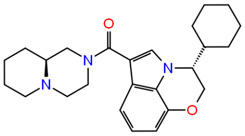	0.3981	[38]
17k	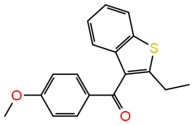	1600	[39]
19	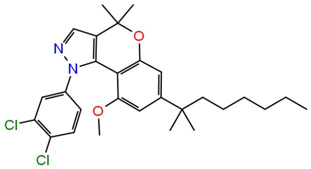	2256	[40]
26	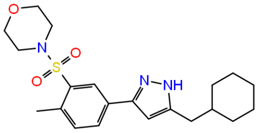	87	[25]
26	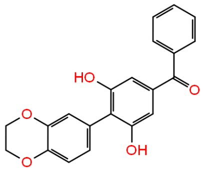	4740	[41]
40	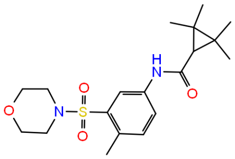	23	[25]
45	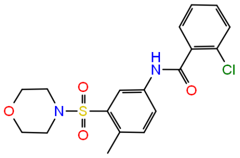	170	[25]

52	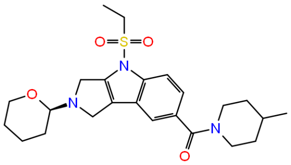	17.6	26
2-AG	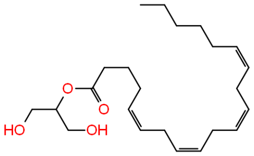	1400	29
ALICB459	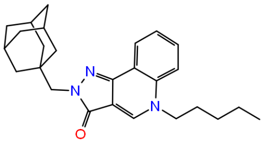	0.39	42
AM-10257	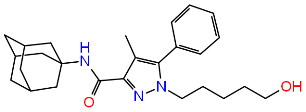	0.08	3
AM-1241	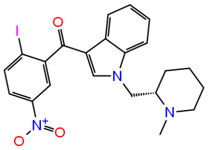	7.94	5
AM-2233	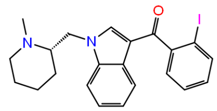	9.2	7
AM-251	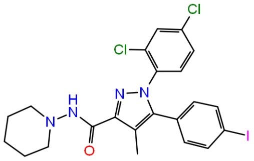	110	30
AM-281	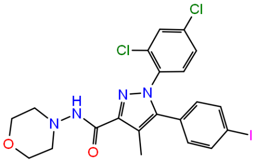	4870	17
AM-630	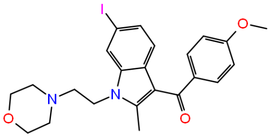	31.2	6
AM-7528	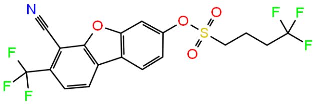	317	8
Anandamide	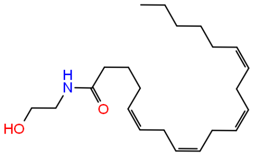	180	31

BAY 59-3074	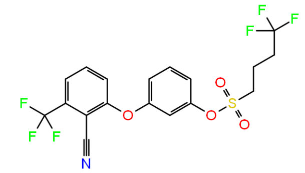	45.5	8
Cannabidivarin	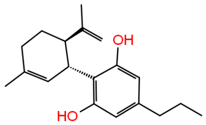	3970	32
Cannabigerol	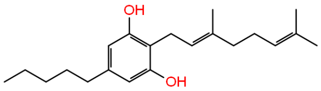	2919	32
CP-55,940	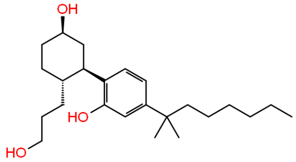	0.79	10
GW-405,833	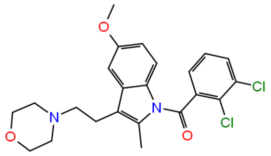	3.92	11
Honokiol	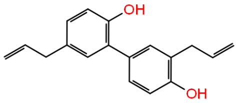	5610	43
Ibipinabant (SLV319)	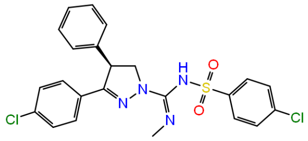	7943	33
JTE-907	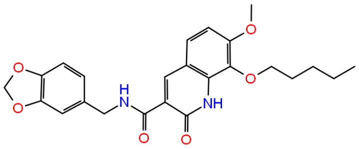	35.9	44
JWH-015	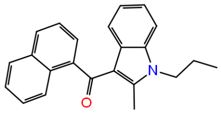	35	12
JWH-133	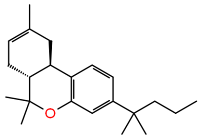	3.4	13
JWH-213	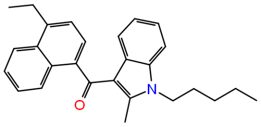	0.42	34
JWH-241	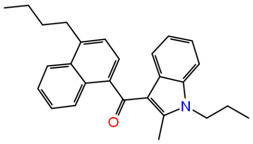	49	34

MDMB-Fubinaca	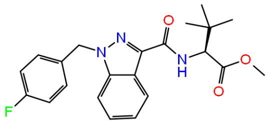	1.14	[15]
Methanandamide	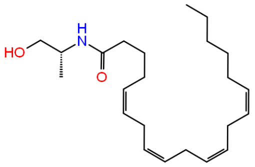	220	[35]
NNEI	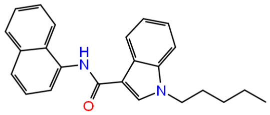	60.09	[15]
Rimonabant	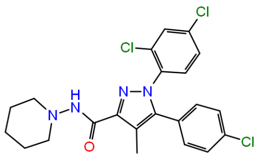	313	[36]
Sch35966	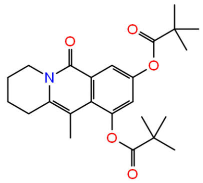	6.8	[28]
SR-144,528	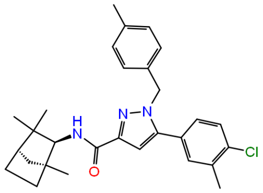	5.6	[6]
Surinabant	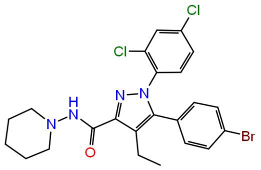	442	[37]
WIN 55,212-2	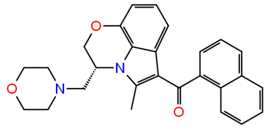	5.36	[17]

Table S8: Thresholds used for specific stages of the virtual screening procedure.

**Stage**	**Threshold**
Pharmacophore screening	Pharmacophore-Fit function value > 60
Preliminary docking (5ZTY)	Docking score ≤ -9
Docking (5ZTY, 6KPC, 6PT0)	Docking score ≤ -10
MM–GBSA (5ZTY, 6KPC, 6PT0)	$\Delta G_{bind} \leq -80$ kcal/mol (5ZTY, 6KPC) or ≤ -90 kcal/mol (6PT0)
Physichemical filtration	Lipinski's and Vebers's rules, logP ≥ 3
QSAR	Predicted $K_i \leq 1000$ nM

Table S9: Statistical parameters of the prepared QSAR models.

**Model code**	**Score**	$\mathbf{R^2}$	$\mathbf{Q^2}$	**SD**	**RMSE**
kpls_molprint2D_40	0.5264	0.6432	0.5696	0.7046	0.7733
kpls_dendritic_8	0.5127	0.8228	0.6322	0.4965	0.7135
kpls_molprint2D_15	0.5102	0.5485	0.5244	0.7919	0.8129
kpls_molprint2D_22	0.5084	0.7526	0.5990	0.5879	0.7448
kpls_linear_31	0.5076	0.8186	0.6275	0.5021	0.7186
kpls_dendritic_40	0.5047	0.7880	0.6131	0.5426	0.7332
kpls_dendritic_31	0.5031	0.7885	0.6113	0.5421	0.7341
kpls_radial_40	0.5025	0.6851	0.5696	0.6617	0.7733
kpls_linear_8	0.5012	0.8465	0.6347	0.4622	0.7111
kpls_dendritic_37	0.5005	0.6705	0.5619	0.6763	0.7796

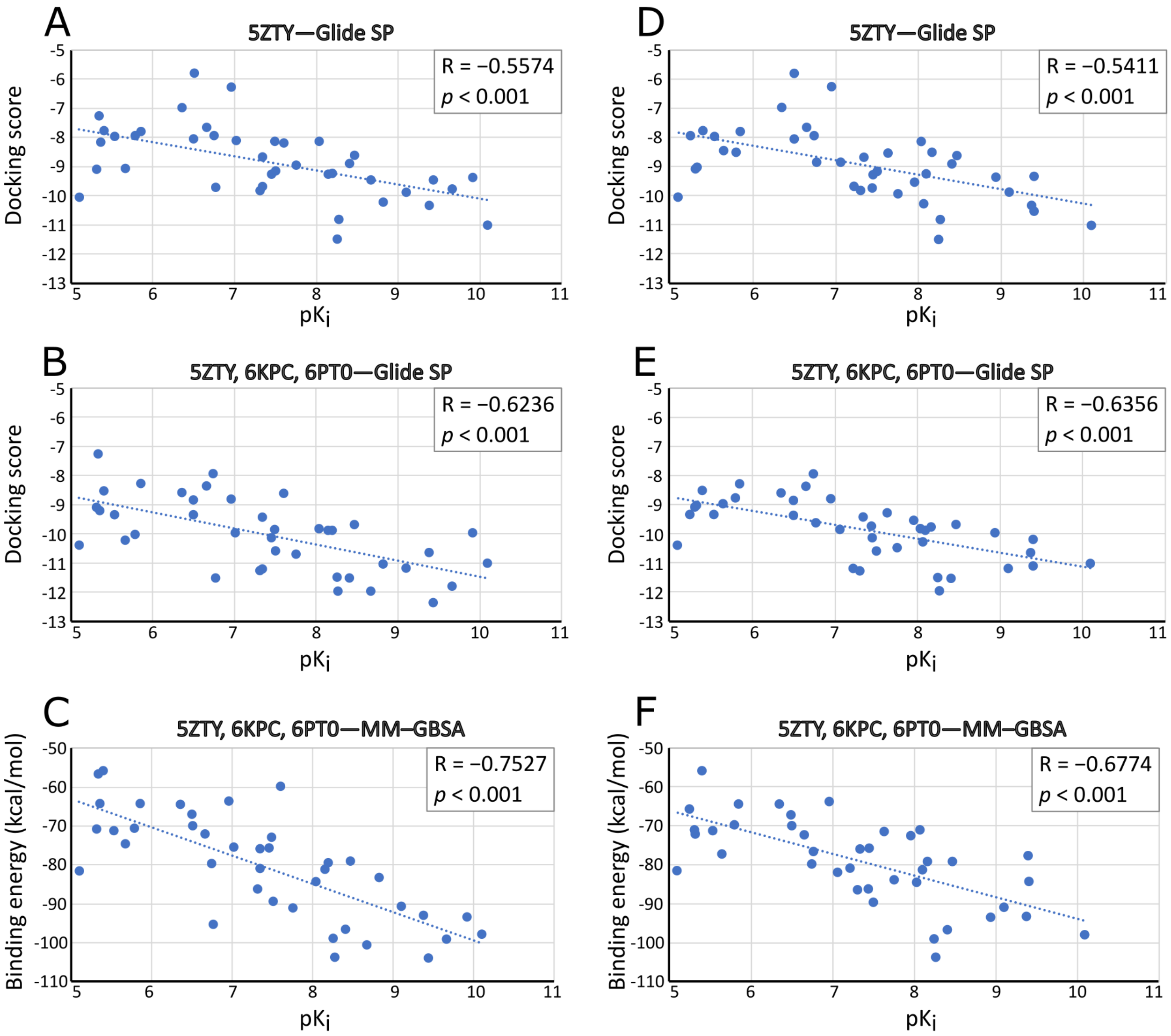

Figure S6: Selected scatter plots from docking and MM–GBSA validation. (A) Docking score–pK_i correlation from test docking to PDB ID: 5ZTY. (B, C) Correlation of pK_i and best docking score (B) or MM–GBSA binding energy (C) after docking to PDB IDs: 5ZTY and 6KPC and 6PT0 MD-derived model. (D-F) Scatter plots from confirmatory validation of the aforementioned combinations of methods and CB2 models, conducted using modified test set (Table S6).

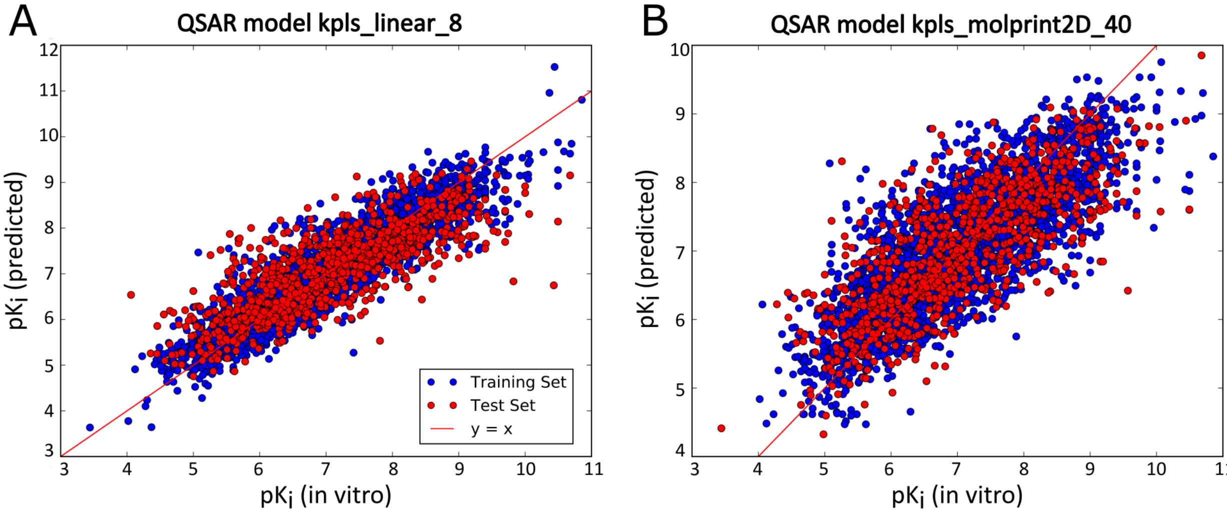

Figure S7: Selected scatter plots from QSAR tests. Correlation of QSAR-predicted and in vitro pK_i values for training and test sets for the best QSAR models based on Q^2 and R^2 (A) or on AutoQSAR score (B). Specific values are deposited in Table S9.

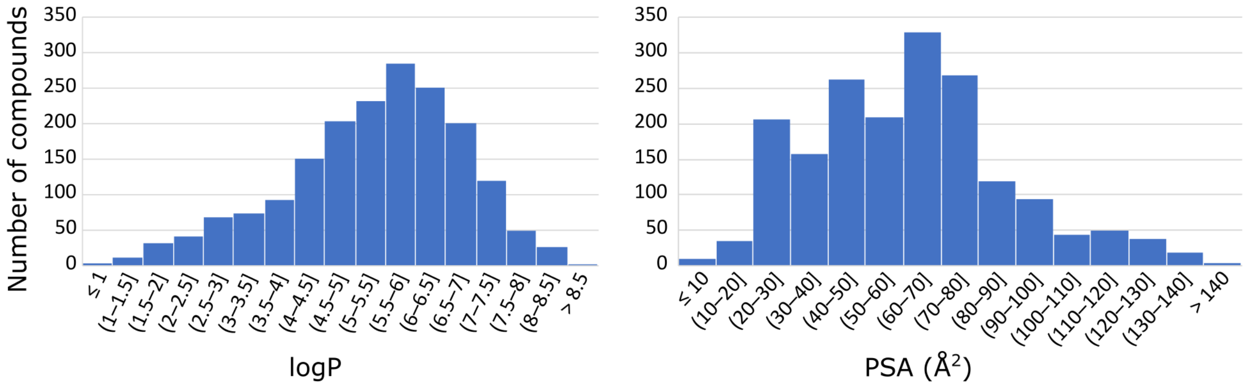

Figure S8: Histograms of computed logP and PSA of CB2 ligands with $K_i \leq 100$ nM deposited in ChEMBL.

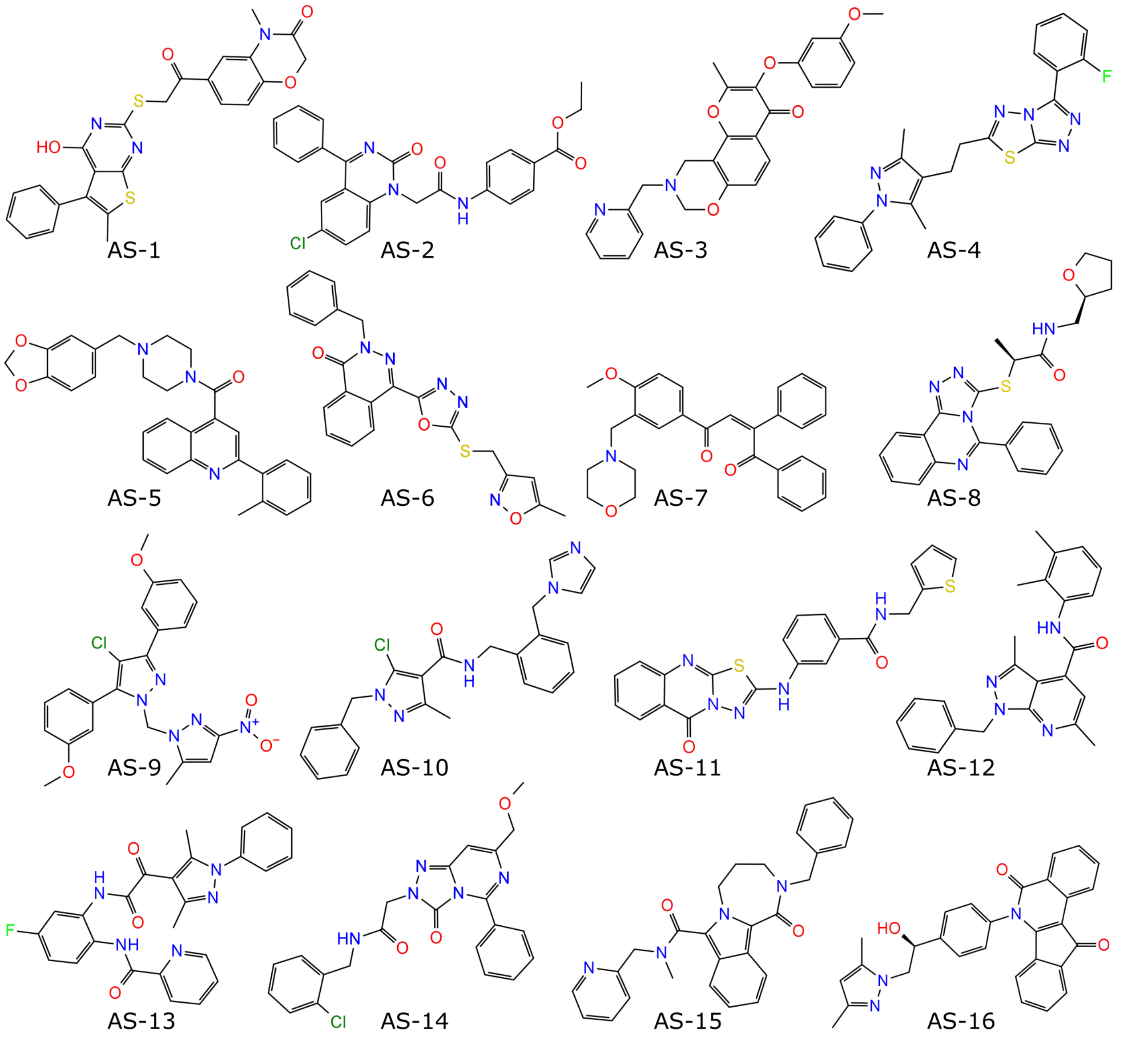

Figure S9: Structural formulas of the compounds selected for the in vitro assay.

Table S10: Calculated physicochemical properties of the compounds selected for the in vitro assay.

ID	MW (g/mol)	LogP	HBD	HBA	PSA (Å^2)	No. rot. bonds
AS-1	477.55	4.2	1	8	106.9	5
AS-2	461.90	4.3	1	8	109.7	6
AS-3	430.46	3.8	0	8	70.6	5
AS-4	418.49	5.8	0	4	59.1	3
AS-5	465.55	4.6	0	8	58.4	4
AS-6	431.47	4.7	0	6	100.0	5
AS-7	441.53	4.1	0	8	67.4	9
AS-8	433.53	3.8	1	7	83.9	6
AS-9	453.88	5.2	0	5	94.2	5
AS-10	419.91	5.2	1	6	66.2	7
AS-11	433.50	4.0	2	8	103.8	5
AS-12	384.48	5.3	1	5	55.5	4
AS-13	457.46	4.5	1	8	120.9	5
AS-14	437.89	3.5	1	8	98.9	7
AS-15	438.53	5.0	0	7	63.6	5
AS-16	461.52	4.4	1	8	86.6	4

Table S11: Results of the K_i determination with [^{3}H]CP-55,940 displacement assay.

ID	ZINC ID	pK_i ± SEM	K_i (μM, 95% CI)
AS-1	ZINC000009043923	no activity	no activity
AS-2	ZINC000010433568	no activity	no activity
AS-3	ZINC000012442653	no activity	no activity
AS-4	ZINC000013691866	no activity	no activity
AS-5	ZINC000013893769	6.68 ± 0.10	0.21 (0.13–0.35)
AS-6	ZINC000020066886	no activity	no activity
AS-7	ZINC000020414208	7.18 ± 0.07	0.065 (0.047–0.090)
AS-8[a]	ZINC000021727012	5.2 ± 0.09	6.37 (4.1–9.9)
AS-9	ZINC000022535815	5.68 ± 0.36	2.1 (0.3–12.2)
AS-10	ZINC000025665877	4.74 ± 0.10	17.9 (10.9–29.4)
AS-11	ZINC000032952152	no activity	no activity
AS-12	ZINC000036615775	no activity	no activity
AS-13	ZINC000059352726	no activity	no activity
AS-14	ZINC000090610158	no activity	no activity
AS-15[b]	ZINC000306134726	no activity	no activity
AS-16[a]	ZINC000825148912	no activity	no activity
WIN 55,212-2 (reference)		8.05 ± 0.06	0.0088 (0.0065–0.012)

[a] Racemic mixture; [b] trifluoroacetic acid salt.

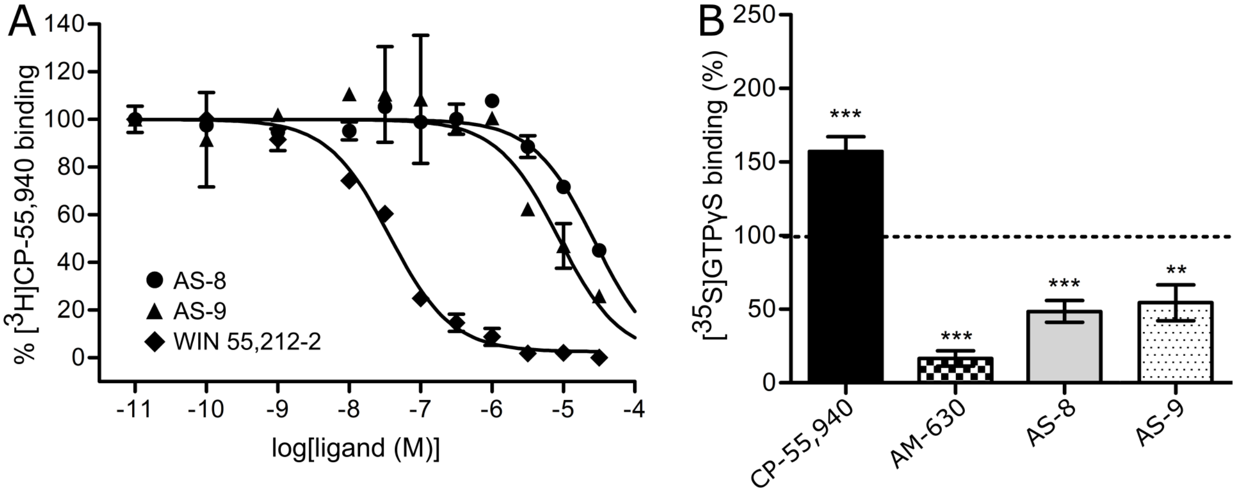

Figure S10: (A) Radioligand displacement curves for AS-8 and AS-9, along with a reference compound WIN 55,212-2. (B) Inhibition of CP-55,940-stimulated [^{35}S]GTPγS at the CB2 receptor by the compounds at 10 μM. Results were expressed as mean percent of basal [^{35}S]GTPγS binding in the presence of 100 nM CP-55,940 as stimulating ligand. AM-630 served as a reference CB2 antagonist. Basal binding was set to 100% and is represented by the dotted line. Data was collected from three separate experiments and analyzed with the two-tailed t test. Statistical significance was depicted as follows: ** $p < 0.01$; *** $p < 0.001$.

Table S12: Different QSAR models' prediction of pK_i of the screening compounds tested in vitro and known potent CB2 ligands well-placed in virtual screening.

ID/Name	All models	Top 5 models (Q^2)	kpls_linear_8	kpls_molprint2D_40
AS-1	6.4	6.7	6.8	5.0
AS-2	6.1	6.5	6.2	5.7
AS-3	6.5	6.8	6.9	5.9
AS-4	6.5	6.4	6.4	6.5
AS-5	7.3	7.1	7.1	7.6
AS-6	6.5	6.5	6.5	6.7
AS-7	6.7	6.9	6.9	6.8
AS-8	6.3	6.6	6.7	5.8
AS-9	6.2	6.4	6.3	6.5
AS-10	6.2	6.3	6.2	6.1
AS-11	6.4	6.6	6.5	6.8
AS-12	6.6	6.7	6.6	6.3
AS-13	6.4	6.4	6.2	6.6
AS-14	6.1	6.5	6.5	4.7
AS-15	6.7	6.7	6.7	6.5
AS-16	7.4	7.1	7.1	8.0
WIN 55.212-2	8.4	8.3	8.3	9.2
MN-25	8.0	8.1	8.0	7.8
MN-25 2-methyl derivative[a]	7.3	7.3	7.3	6.8

[a] ZINC000013519818.

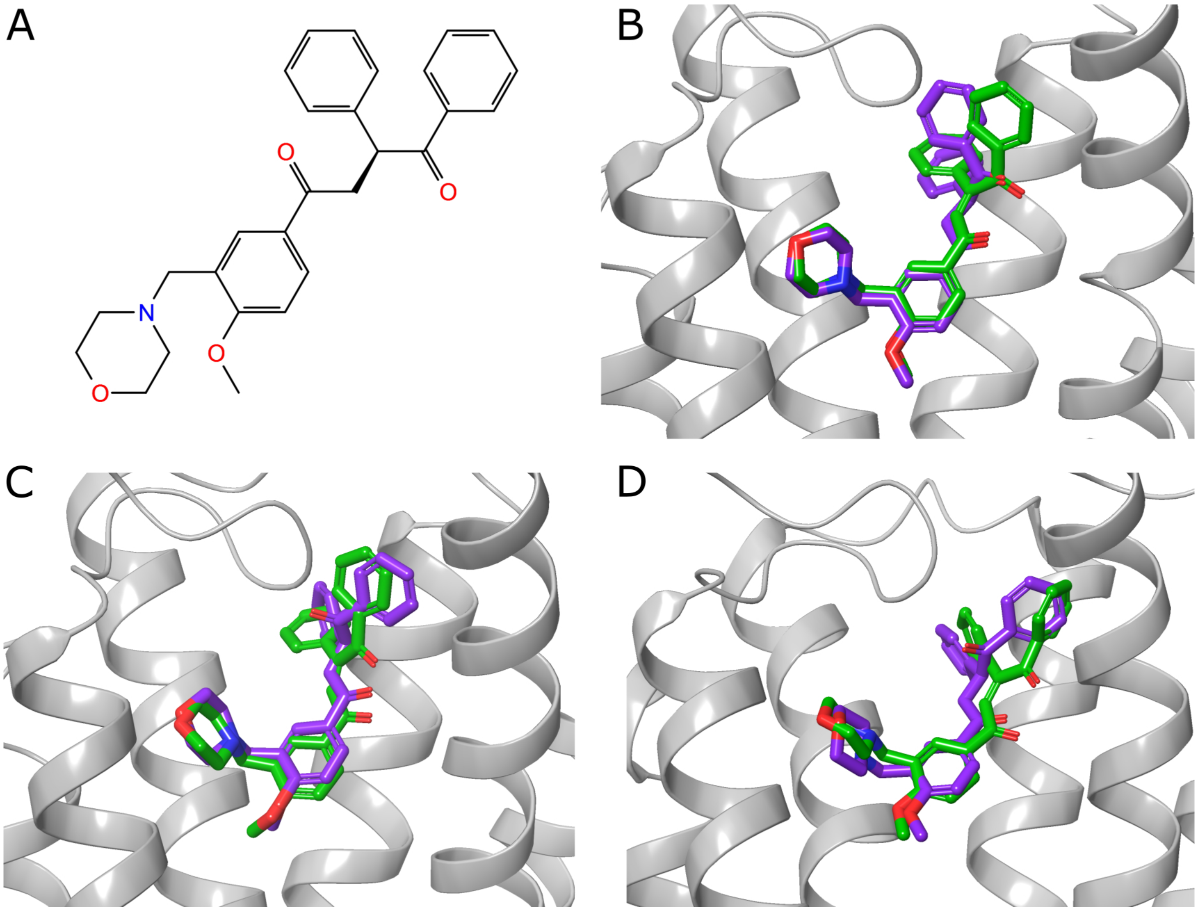

Figure S11: Docking results for AS-7-1. (A) AS-7-1 structural formula. (B–D) Putative binding modes of AS-7-1 (magenta) compared to AS-7 (green) docked to CB2 models based on PDB IDs: 5ZTY (B), 6KPC (C), and 6PT0 MD-derived structure (D).

Table S13: Docking, MM–GBSA and QSAR results for AS-7-1.

**5ZTY**		**6KPC**		**6PT0**		**Pred.**	**Pred.**
**Docking score**	**ΔG_{bind} (kcal/mol)**	**Docking score**	**ΔG_{bind} (kcal/mol)**	**Docking score**	**ΔG_{bind} (kcal/mol)**	**pK_i**	**pK_i SD**
−9.9	−74.4	−7.5	−52.1	−10.2	−92.6	6.7	0.4

Table S14: Average computed physicochemical properties of CB2 ligands with $K_i \leq 100$ nM deposited in ChEMBL.

**Property**	**Value**
MW (g/mol)	421.10
logP	5.3
HBD	0.6
HBA	5.2
PSA (Å^2)	61.5
No. of rotatable bonds	6.6

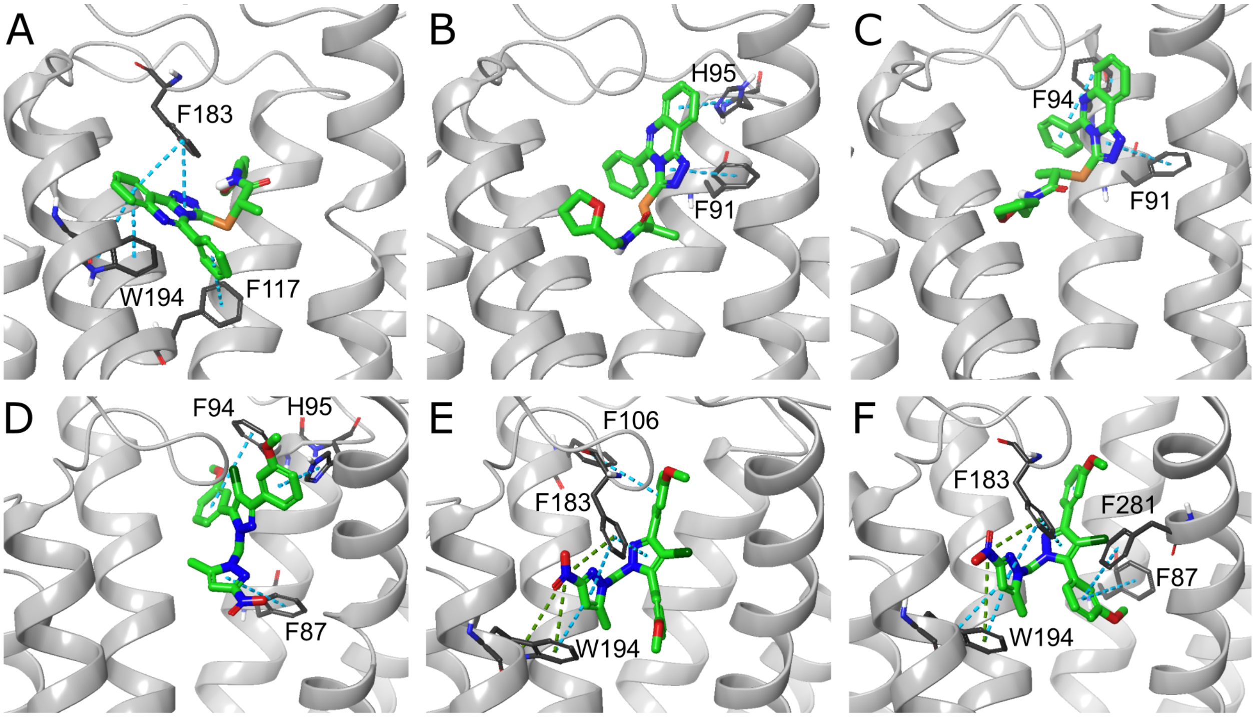

Figure S12: Putative binding modes of AS-8 and AS-9. (A–C) AS-8 (green) docked to CB2 models based on PDB IDs: 5ZTY (A), 6KPC (B), and 6PT0 MD-derived structure (C). (D–F) AS-9 (green) docked to CB2 models based on PDB IDs: 5ZTY (D), 6KPC (E), and 6PT0 MD-derived structure (F). Teal dashed line—π–π interaction, green dashed line—π–cation interaction.

Abbreviations Used

[^{35}S]GTPγS, [^{35}S] guanosine 5'-[γ-thio]triphosphate; CB2, cannabinoid receptor type 2; CI, confidence interval; EF, enrichment factor; HBA, (number of) hydrogen bond acceptor(s); HBD, (number of) hydrogen bond donor(s); IC_{50}, half maximal inhibitory concentration; K_i, inhibition constant; MD, molecular dynamics; MM–GBSA, molecular mechanics–generalized Born surface area; MW, molecular weight; PDB, Protein Data Bank; PSA, polar surface area; QSAR, quantitative structure–activity relationship; RMSD, root-mean-square deviation; RMSE, root-mean-square error; SD, standard deviation; SEM, standard error of measurement; SP, standard precision.

References

(1) Abraham, M. J.; Murtola, T.; Schulz, R.; Páll, S.; Smith, J. C.; Hess, B.; Lindahl, E. GROMACS: High Performance Molecular Simulations Through Multi-Level Parallelism from Laptops to Supercomputers. *SoftwareX* **2015**, *1*, 19–25.

(2) Daura, X.; Gademann, K.; Jaun, B.; Seebach, D.; Van Gunsteren, W. F.; Mark, A. E. Peptide Folding: When Simulation Meets Experiment. *Angew. Chem., Int. Ed.* **1999**, *38*, 236–240.

(3) Li, X.; Hua, T.; Vemuri, K.; Ho, J.-H.; Wu, Y.; Wu, L.; Popov, P.; Benchama, O.; Zvonok, N.; Qu, L.; Han, G. W.; Iyer, M. R.; Cinar, R.; Coffey, N. J.; Wang, J.; Wu, M.; Katritch, V.; Zhao, S.; Kunos, G.; Bohn, L. M.; Makriyannis, A.; Stevens, R. C.; Liu, Z.-J. Crystal Structure of the Human Cannabinoid Receptor CB2. *Cell* **2019**, *176*, 459–467.

(4) Hua, T.; Li, X.; Wu, L.; Iliopoulos-Tsoutsouvas, C.; Wang, Y.; Wu, M.; Shen, L.; Johnston, C. A.; Nikas, S. P.; Song, F.; Song, X.; Yuan, S.; Sun, Q.; Wu, Y.; Jiang, S.; Grim, T. W.; Benchama, O.; Stahl, E. L.; Zvonok, N.; Zhao, S.; Bohn, L. M.; Makriyan-

nis, A.; Liu, Z.-J. Activation and Signaling Mechanism Revealed by Cannabinoid Receptor-Gi Complex Structures. *Cell* **2020**, *180*, 655–665.

(5) Han, S.; Thatte, J.; Buzard, D. J.; Jones, R. M. Therapeutic Utility of Cannabinoid Receptor Type 2 (CB2) Selective Agonists. *J. Med. Chem.* **2013**, *56*, 8224–8256.

(6) Ross, R. A.; Brockie, H. C.; Stevenson, L. A.; Murphy, V. L.; Templeton, F.; Makriyannis, A.; Pertwee, R. G. Agonist-Inverse Agonist Characterization at CB1 and CB2 Cannabinoid Receptors of L759633, L759656 and AM630. *Br. J. Pharmacol.* **1999**, *126*, 665–672.

(7) Pei, Y.; Mercier, R. W.; Anday, J. K.; Thakur, G. A.; Zvonok, A. M.; Hurst, D.; Reggio, P. H.; Janero, D. R.; Makriyannis, A. Ligand-Binding Architecture of Human CB2 Cannabinoid Receptor: Evidence for Receptor Subtype-Specific Cinding Motif and Modeling GPCR Activation. *Chem. Biol.* **2008**, *15*, 1207–1219.

(8) Teng, H.; Thakur, G. A.; Makriyannis, A. Conformationally Constrained Analogs of BAY 59–3074 as Novel Cannabinoid Receptor Ligands. *Bioorg. Med. Chem. Lett.* **2011**, *21*, 5999–6002.

(9) Showalter, V. M.; Compton, D. R.; Martin, B. R.; Abood, M. E. Evaluation of Binding in a Transfected Cell Line Expressing a Peripheral Cannabinoid Receptor (CB2): Identification of Cannabinoid Receptor Subtype Selective Ligands. *J. Pharmacol. Exp. Ther.* **1996**, *278*, 989–999.

(10) Yao, B.; Mukherjee, S.; Fan, Y.; Garrison, T.; Daza, A.; Grayson, G.; Hooker, B.; Dart, M.; Sullivan, J.; Meyer, M. In Vitro Pharmacological Characterization of AM1241: A Protean Agonist at the Cannabinoid CB2 Receptor? *Br. J. Pharmacol.* **2006**, *149*, 145–154.

(11) Valenzano, K. J.; Tafesse, L.; Lee, G.; Harrison, J. E.; Boulet, J. M.; Gottshall, S. L.; Mark, L.; Pearson, M. S.; Miller, W.; Shan, S.; Rabadi, L.; Rotshteyn, Y.; Chaf-

fer, S. M.; Turchin, P. I.; Elsemore, D. A.; Toth, M.; Koetzner, L.; Whiteside, G. T. Pharmacological and Pharmacokinetic Characterization of the Cannabinoid Receptor 2 Agonist, GW405833, Utilizing Rodent Models of Acute and Chronic Pain, Anxiety, Ataxia and Catalepsy. *Neuropharmacology* **2005**, *48*, 658–672.

(12) Frost, J. M.; Dart, M. J.; Tietje, K. R.; Garrison, T. R.; Grayson, G. K.; Daza, A. V.; El-Kouhen, O. F.; Miller, L. N.; Li, L.; Yao, B. B.; Hsieh, G. C.; Pai, M.; Zhu, C. Z.; Chandran, P.; Meyer, M. D. Indol-3-yl-tetramethylcyclopropyl Ketones: Effects of Indole Ring Substitution on CB2 Cannabinoid Receptor Activity. *J. Med. Chem.* **2008**, *51*, 1904–1912.

(13) Pertwee, R. G.; Howlett, A.; Abood, M. E.; Alexander, S.; Di Marzo, V.; Elphick, M.; Greasley, P.; Hansen, H.; Kunos, G.; Mackie, K.; Mechoulam, R.; Ross, R. International Union of Basic and Clinical Pharmacology. LXXIX. Cannabinoid Receptors and Their Ligands: Beyond CB1 and CB2. *Pharmacol. Rev.* **2010**, *62*, 588–631.

(14) Markt, P.; Feldmann, C.; Rollinger, J. M.; Raduner, S.; Schuster, D.; Kirchmair, J.; Distinto, S.; Spitzer, G. M.; Wolber, G.; Laggner, C.; Altmann, K.-H.; Langer, T.; Jürg, G. Discovery of Novel CB2 Receptor Ligands by a Pharmacophore-Based Virtual Screening Workflow. *J. Med. Chem.* **2009**, *52*, 369–378.

(15) Gamage, T. F.; Farquhar, C. E.; Lefever, T. W.; Marusich, J. A.; Kevin, R. C.; McGregor, I. S.; Wiley, J. L.; Thomas, B. F. Molecular and Behavioral Pharmacological Characterization of Abused Synthetic Cannabinoids MMB- and MDMB-FUBINACA, MN-18, NNEI, CUMYL-PICA, and 5-Fluoro-CUMYL-PICA. *J. Pharmacol. Exp. Ther.* **2018**, *365*, 437–446.

(16) Lange, J. H.; Attali, A.; van der Neut, M. A.; Wals, H. C.; Mulder, A.; Zilaout, H.; Duursma, A.; van Aken, H. H.; van Vliet, B. J. Two Distinct Classes of Novel Pyrazo-

linecarboxamides as Potent Cannabinoid CB1 Receptor Agonists. *Bioorg. Med. Chem. Lett.* **2010**, *20*, 4992–4998.

(17) Behrenswerth, A.; Volz, N.; Toräng, J.; Hinz, S.; Bräse, S.; Müller, C. E. Synthesis and Pharmacological Evaluation of Coumarin Derivatives as Cannabinoid Receptor Antagonists and Inverse Agonists. *Bioorg. Med. Chem.* **2009**, *17*, 2842–2851.

(18) Bhattacharjee, H.; Gurley, S. N.; Moore II, B. M. Design and Synthesis of Novel Tri-Aryl CB2 Selective Cannabinoid Ligands. *Bioorg. Med. Chem. Lett.* **2009**, *19*, 1691–1693.

(19) Rhee, M.-H.; Vogel, Z.; Barg, J.; Bayewitch, M.; Levy, R.; Hanuš, L.; Breuer, A.; Mechoulam, R. Cannabinol Derivatives: Binding to Cannabinoid Receptors and Inhibition of Adenylylcyclase. *J. Med. Chem.* **1997**, *40*, 3228–3233.

(20) Manera, C.; Cascio, M. G.; Benetti, V.; Allarà, M.; Tuccinardi, T.; Martinelli, A.; Saccomanni, G.; Vivoli, E.; Ghelardini, C.; Di Marzo, V.; Luigi Ferrarini, P. New 1,8-Naphthyridine and Quinoline Derivatives as CB2 Selective Agonists. *Bioorg. Med. Chem. Lett.* **2007**, *17*, 6505–6510.

(21) Pasquini, S.; De Rosa, M.; Pedani, V.; Mugnaini, C.; Guida, F.; Luongo, L.; De Chiaro, M.; Maione, S.; Dragoni, S.; Frosini, M.; Ligresti, A.; di Marzo, V.; Corelli, F. Investigations on the 4-Quinolone-3-carboxylic Acid Motif. 4. Identification of New Potent and Selective Ligands for the Cannabinoid Type 2 Receptor with Diverse Substitution Patterns and Antihyperalgesic Effects in Mice. *J. Med. Chem.* **2011**, *54*, 5444–5453.

(22) Kai, H.; Morioka, Y.; Murashi, T.; Morita, K.; Shinonome, S.; Nakazato, H.; Kawamoto, K.; Hanasaki, K.; Takahashi, F.; Mihara, S.-i.; Arai, T.; Abe, K.; Okabe, H.; Baba, T.; Yoshikawa, T.; Takenaka, H. 2-Arylimino-5,6-dihydro-4H-1,3-thiazines as a New Class of Cannabinoid Receptor Agonists. Part 1: Discovery of CB2 Receptor Selective Compounds. *Bioorg. Med. Chem. Lett.* **2007**, *17*, 4030–4034.

(23) Kusakabe, K.-i.; Iso, Y.; Tada, Y.; Sakagami, M.; Morioka, Y.; Chomei, N.; Shinonome, S.; Kawamoto, K.; Takenaka, H.; Yasui, K.; Hamana, H.; Hanasaki, K. Selective CB2 Agonists with Anti-Pruritic Activity: Discovery of Potent and Orally Available Bicyclic 2-Pyridones. *Bioorg. Med. Chem.* **2013**, *21*, 3154–3163.

(24) Lucchesi, V.; Hurst, D. P.; Shore, D. M.; Bertini, S.; Ehrmann, B. M.; Allara, M.; Lawrence, L.; Ligresti, A.; Minutolo, F.; Saccomanni, G.; Sharir, H.; Macchia, M.; Di Marzo, V.; Abood, M. E.; Reggio, P. H.; Manera, C. CB2-Selective Cannabinoid Receptor Ligands: Synthesis, Pharmacological Evaluation, and Molecular Modeling Investigation of 1,8-Naphthyridin-2(1H)-one-3-carboxamides. *J. Med. Chem.* **2014**, *57*, 8777–8791.

(25) Goodman, A. J.; Ajello, C. W.; Worm, K.; Le Bourdonnec, B.; Savolainen, M. A.; O'Hare, H.; Cassel, J. A.; Stabley, G. J.; DeHaven, R. N.; Labuda, C. J.; Koblish, M.; Little, P. J.; Brogdon, B. L.; Smith, S. A.; Dolle, R. E. CB2 Selective Sulfamoyl Benzamides: Optimization of the Amide Functionality. *Bioorg. Med. Chem. Lett.* **2009**, *19*, 309–313.

(26) Pagé, D.; Yang, H.; Brown, W.; Walpole, C.; Fleurent, M.; Fyfe, M.; Gaudreault, F.; St-Onge, S. New 1,2,3,4-Tetrahydropyrrolo[3,4-b]indole Derivatives as Selective CB2 Receptor Agonists. *Bioorg. Med. Chem. Lett.* **2007**, *17*, 6183–6187.

(27) Odan, M.; Ishizuka, N.; Hiramatsu, Y.; Inagaki, M.; Hashizume, H.; Fujii, Y.; Mitsumori, S.; Morioka, Y.; Soga, M.; Deguchi, M.; Yasui, K.; Arimura, A. Discovery of S-777469: An Orally Available CB2 Agonist as an Antipruritic Agent. *Bioorg. Med. Chem. Lett.* **2012**, *22*, 2803–2806.

(28) Gonsiorek, W.; Lunn, C.; Fan, X.; Deno, G.; Kozlowski, J.; Hipkin, R. Sch35966 is a Potent, Selective Agonist at the Peripheral Cannabinoid Receptor (CB2) in Rodents and Primates. *Br. J. Pharmacol.* **2007**, *151*, 1262–1271.

(29) Brizzi, A.; Cascio, M. G.; Frosini, M.; Ligresti, A.; Aiello, F.; Biotti, I.; Brizzi, V.; Pertwee, R. G.; Corelli, F.; Di Marzo, V. Resorcinol-sn-glycerol Derivatives: Novel 2-Arachidonoylglycerol Mimetics Endowed with High Affinity and Selectivity for Cannabinoid Type 1 Receptor. *J. Med. Chem.* **2011**, *54*, 8278–8288.

(30) Urbani, P.; Cascio, M. G.; Ramunno, A.; Bisogno, T.; Saturnino, C.; Di Marzo, V. Novel Sterically Hindered Cannabinoid CB1 Receptor Ligands. *Bioorg. Med. Chem.* **2008**, *16*, 7510–7515.

(31) Balas, L.; Durand, T.; Saha, S.; Johnson, I.; Mukhopadhyay, S. Total Synthesis of Photoactivatable or Fluorescent Anandamide Probes: Novel Bioactive Compounds with Angiogenic Activity. *J. Med. Chem.* **2009**, *52*, 1005–1017.

(32) Husni, A. S.; McCurdy, C. R.; Radwan, M. M.; Ahmed, S. A.; Slade, D.; Ross, S. A.; ElSohly, M. A.; Cutler, S. J. Evaluation of Phytocannabinoids from High-Potency Cannabis Sativa Using In Vitro Bioassays to Determine Structure–Activity Relationships for Cannabinoid Receptor 1 and Cannabinoid Receptor 2. *Med. Chem. Res.* **2014**, *23*, 4295–4300.

(33) Lange, J. H.; Coolen, H. K.; van Stuivenberg, H. H.; Dijksman, J. A.; Herremans, A. H.; Ronken, E.; Keizer, H. G.; Tipker, K.; McCreary, A. C.; Veerman, W.; Wals, H. C.; Stork, B.; Verveer, P. C.; den Hartog, A. P.; de Jong, N. M.; Adolfs, T. J.; Hoogendoorn, J.; Kruse, C. G. Synthesis, Biological Properties, and Molecular Modeling Investigations of Novel 3,4-Diarylpyrazolines as Potent and Selective CB1 Cannabinoid Receptor Antagonists. *J. Med. Chem.* **2004**, *47*, 627–643.

(34) Huffman, J. W.; Zengin, G.; Wu, M.-J.; Lu, J.; Hynd, G.; Bushell, K.; Thompson, A. L.; Bushell, S.; Tartal, C.; Hurst, D. P.; Reggio, P. H.; Selley, D. E.; Cassidy, M. P.; Wiley, J. L.; Martin, B. R. Structure–Activity Relationships for 1-Alkyl-3-(1-naphthoyl)indoles at the Cannabinoid CB1 and CB2 Receptors: Steric and Elec-

tronic Effects of Naphthoyl Substituents. New Highly Selective CB2 Receptor Agonists. *Bioorg. Med. Chem.* **2005**, *13*, 89–112.

(35) Liu, Y.; Ji, L.; Eno, M.; Kudalkar, S.; Li, A.-L.; Schimpgen, M.; Benchama, O.; Morales, P.; Xu, S.; Hurst, D.; Wu, S.; Mohammad, K. A.; Wood, J. T.; Zvonok, N.; Papahatjis, D. P.; Zhou, H.; Honrao, C.; Mackie, K.; Reggio, P.; Hohmann, A. G.; Marnett, L. J.; Makriyannis, A.; Nikas, S. P. (R)-N-(1-Methyl-2-hydroxyethyl)-13-(S)-methyl-arachidonamide (AMG315): A Novel Chiral Potent Endocannabinoid Ligand with Stability to Metabolizing Enzymes. *J. Med. Chem.* **2018**, *61*, 8639–8657.

(36) Francisco, M. E. Y.; Seltzman, H. H.; Gilliam, A. F.; Mitchell, R. A.; Rider, S. L.; Pertwee, R. G.; Stevenson, L. A.; Thomas, B. F. Synthesis and Structure–Activity Relationships of Amide and Hydrazide Analogues of the Cannabinoid CB1 Receptor Antagonist N-(Piperidinyl)-5-(4-chlorophenyl)-1-(2,4-dichlorophenyl)-4-methyl-1 H-pyrazole-3-carboxamide (SR141716). *J. Med. Chem.* **2002**, *45*, 2708–2719.

(37) Rinaldi-Carmona, M.; Barth, F.; Congy, C.; Martinez, S.; Oustric, D.; Pério, A.; Poncelet, M.; Maruani, J.; Arnone, M.; Finance, O.; Soubrié, P.; Le Fur, G. SR147778 [5-(4-Bromophenyl)-1-(2,4-dichlorophenyl)-4-ethyl-N-(1-piperidinyl)-1H-pyrazole-3-carboxamide], a New Potent and Selective Antagonist of the CB1 Cannabinoid Receptor: Biochemical and Pharmacological Characterization. *J. Pharmacol. Exp. Ther.* **2004**, *310*, 905–914.

(38) Moir, E. M.; Yoshiizumi, K.; Cairns, J.; Cowley, P.; Ferguson, M.; Jeremiah, F.; Kiyoi, T.; Morphy, R.; Tierney, J.; Wishart, G.; York, M.; Baker, J.; Cottney, J. E.; Houghton, A. K.; McPhail, P.; Osprey, A.; Walker, G.; Adam, J. M. Design, Synthesis, and Structure–Activity Relationship Study of Bicyclic Piperazine Analogs of Indole-3-carboxamides as Novel Cannabinoid CB1 Receptor Agonists. *Bioorg. Med. Chem. Lett.* **2010**, *20*, 7327–7330.

(39) Romero-Parra, J.; Mella-Raipan, J.; Palmieri, V.; Allarà, M.; Torres, M. J.; Pessoa-Mahana, H.; Iturriaga-Vásquez, P.; Escobar, R.; Faúndez, M.; Di Marzo, V.; Pessoa-Mahana, C. D. Synthesis, Binding Assays, Cytotoxic Activity and Docking Studies of Benzimidazole and Benzothiophene Derivatives with Selective Affinity for the CB2 Cannabinoid Receptor. *Eur. J. Med. Chem.* **2016**, *124*, 17–35.

(40) Morales, P.; Gómez-Cañas, M.; Navarro, G.; Hurst, D. P.; Carrillo-Salinas, F. J.; Lagartera, L.; Pazos, R.; Goya, P.; Reggio, P. H.; Guaza, C.; Franco, R.; Fernández-Ruiz, J.; Jagerovic, N. Chromenopyrazole, a Versatile Cannabinoid Scaffold with In Vivo Activity in a Model of Multiple Sclerosis. *J. Med. Chem.* **2016**, *59*, 6753–6771.

(41) Alghamdi, S. S.; Mustafa, S. M.; Moore II, B. M. Synthesis and Biological Evaluation of a Ring Analogs of the Selective CB2 Inverse Agonist SMM-189. *Bioorg. Med. Chem.* **2021**, *33*, 116035.

(42) El Bakali, J.; Muccioli, G. G.; Body-Malapel, M.; Djouina, M.; Klupsch, F.; Ghinet, A.; Barczyk, A.; Renault, N.; Chavatte, P.; Desreumaux, P.; Lambert, D. M.; Millet, R. Conformational Restriction Leading to a Selective CB2 Cannabinoid Receptor Agonist Orally Active Against Colitis. *ACS Med. Chem. Lett.* **2015**, *6*, 198–203.

(43) Rempel, V.; Fuchs, A.; Hinz, S.; Karcz, T.; Lehr, M.; Koetter, U.; Muller, C. E. Magnolia Extract, Magnolol, and Metabolites: Activation of Cannabinoid CB2 Receptors and Blockade of the Related GPR55. *ACS Med. Chem. Lett.* **2013**, *4*, 41–45.

(44) Iwamura, H.; Suzuki, H.; Ueda, Y.; Kaya, T.; Inaba, T. In Vitro and In Vivo Pharmacological Characterization of JTE-907, a Novel Selective Ligand for Cannabinoid CB2 Receptor. *J. Pharmacol. Exp. Ther.* **2001**, *296*, 420–425.
