# Supplementary material for: Identification of Novel CB2 Ligands through Virtual Screening and In Vitro Evaluation
Source: J Chem Inf Model. 2023 Jan 24;63(3):1012–27. doi: 10.1021/acs.jcim.2c01503 (PMC9930120; doi:10.1021/acs.jcim.2c01503)

Openlynx Report - HELEN

Sample: 19	Vial:1,2:B,7	ID:CD
File:263722	Date:27-Jan-2003	Time:14:53:41
Description:5164	Submitter:KERI	

Printed: Mon Jan 27 15:06:07 2003

Sample Report:

Sample 19 Vial 1,2:B,7 ID CD File 263722 Date 27-Jan-2003 Time 14:53:41 Description 5164 Submitter KERI

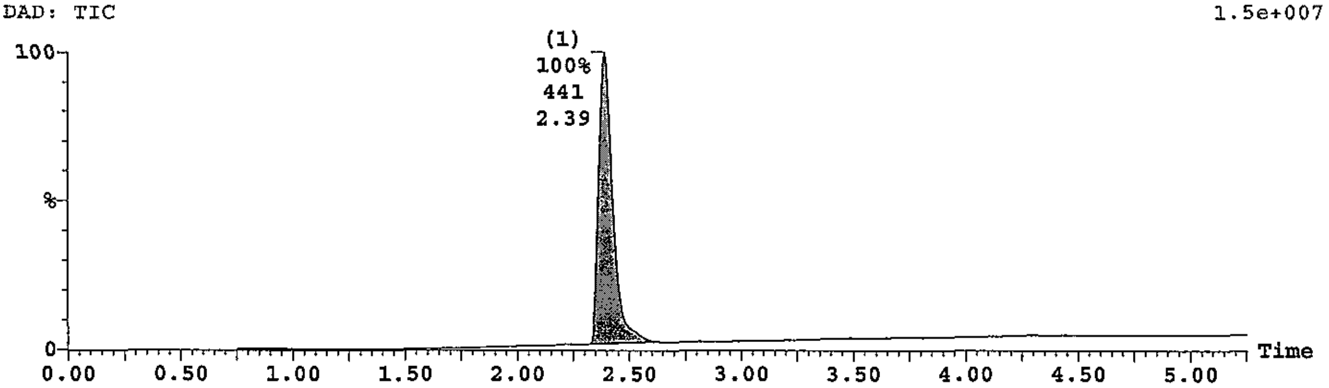

Peak Number	Compound	Time	AreaAbs	Area %Total	Width	Height	Mass Found
1	Found	2.39	.115e+006	100.000	0.310	510e+007	

Peak ID	Compound	Time	Mass Found
1		2.39	

Combine (33:43-(31:32+47:48))

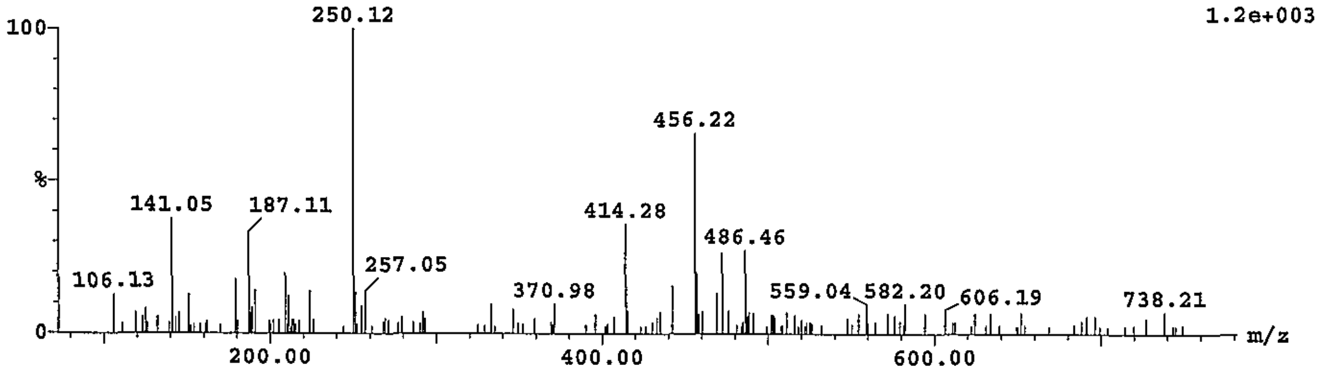

Peak ID	Compound	Time	Mass Found
1	Found	2.39	441

Combine (33:43-(31:32+47:48))

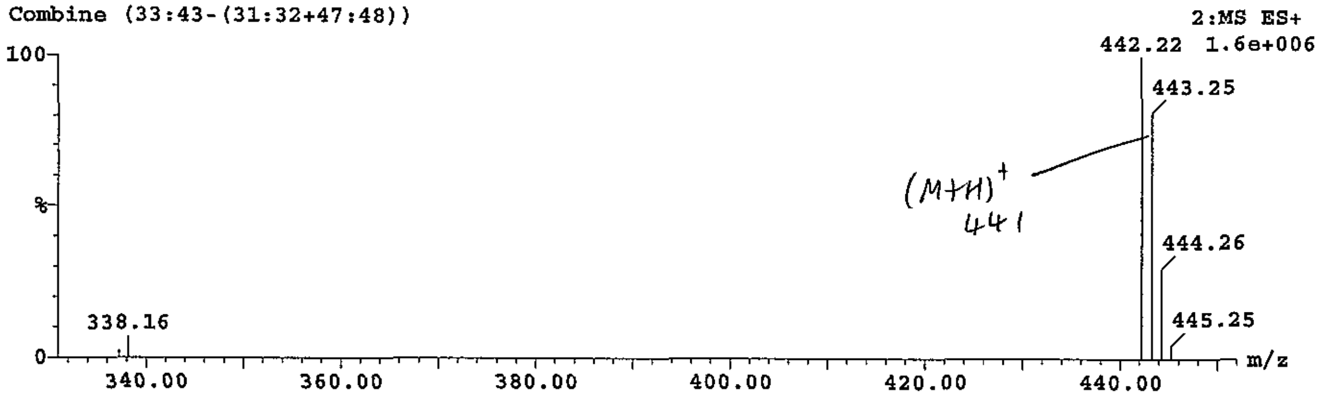

Supplement: Supplementary file 2 — ci2c01503_si_002.zip [file ci2c01503_si_002.zip › CD05164_LCMS.pdf]
