# Supplementary material for: Identification of Novel CB2 Ligands through Virtual Screening and In Vitro Evaluation
Source: J Chem Inf Model. 2023 Jan 24;63(3):1012–27. doi: 10.1021/acs.jcim.2c01503 (PMC9930120; doi:10.1021/acs.jcim.2c01503)
Supplement: Supplementary file 2 — ci2c01503_si_002.zip [file ci2c01503_si_002.zip › CD05164_NMR.pdf]

Analysis number 263722
Compound Code cd 05164
Project Code scr hf2
Chemist Name jom

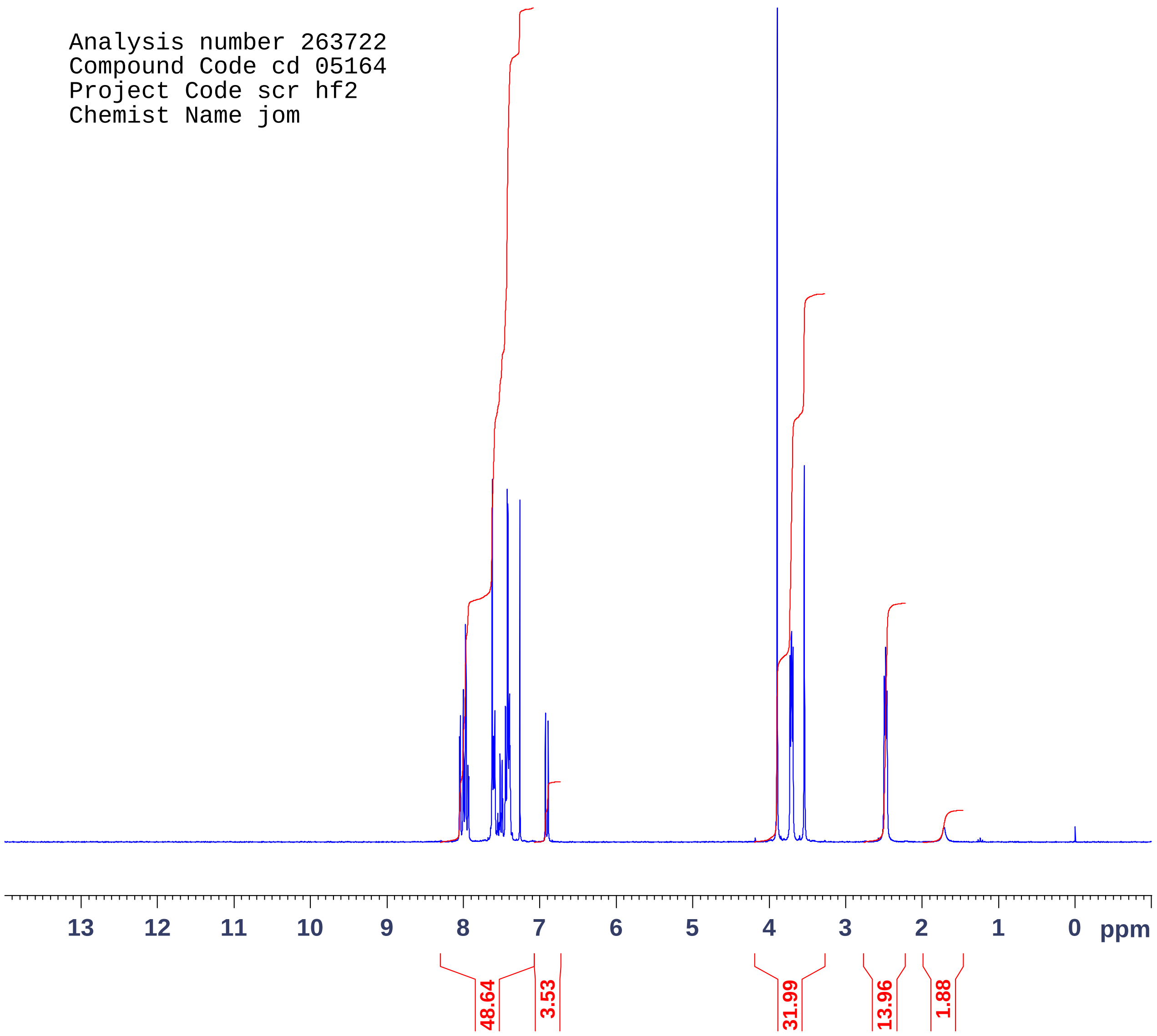

Current Data Parameters

NAME	SAMPLE_STORE
EXPNO	620
PROCNO	1

F2 - Acquisition Parameters

Date_	20070105
Time	0.43
INSTRUM	dpx250
PROBHD	5 mm Dual 13C/
PULPROG	zg30
TD	65536
SOLVENT	CDCl3
NS	16
DS	2
SWH	5175.983 Hz
FIDRES	0.078979 Hz
AQ	6.3308277 sec
RG	574.7
DW	96.600 usec
DE	6.00 usec
TE	294.2 K
D1	1.00000000 sec
MCREST	0.00000000 sec
MCWRK	0.01500000 sec

======== CHANNEL f1 ========

NUC1	1H
P1	9.50 usec
PL1	0.00 dB
SFO1	250.1315447 MHz

F2 - Processing parameters

SI	32768
SF	250.1300008 MHz
WDW	EM
SSB	0
LB	0.30 Hz
GB	0
PC	1.00
