# Supplementary material for: Identification of Novel CB2 Ligands through Virtual Screening and In Vitro Evaluation
Source: J Chem Inf Model. 2023 Jan 24;63(3):1012–27. doi: 10.1021/acs.jcim.2c01503 (PMC9930120; doi:10.1021/acs.jcim.2c01503)
Supplement: Supplementary file 2 — ci2c01503_si_002.zip [file ci2c01503_si_002.zip › F127-0520.pdf]

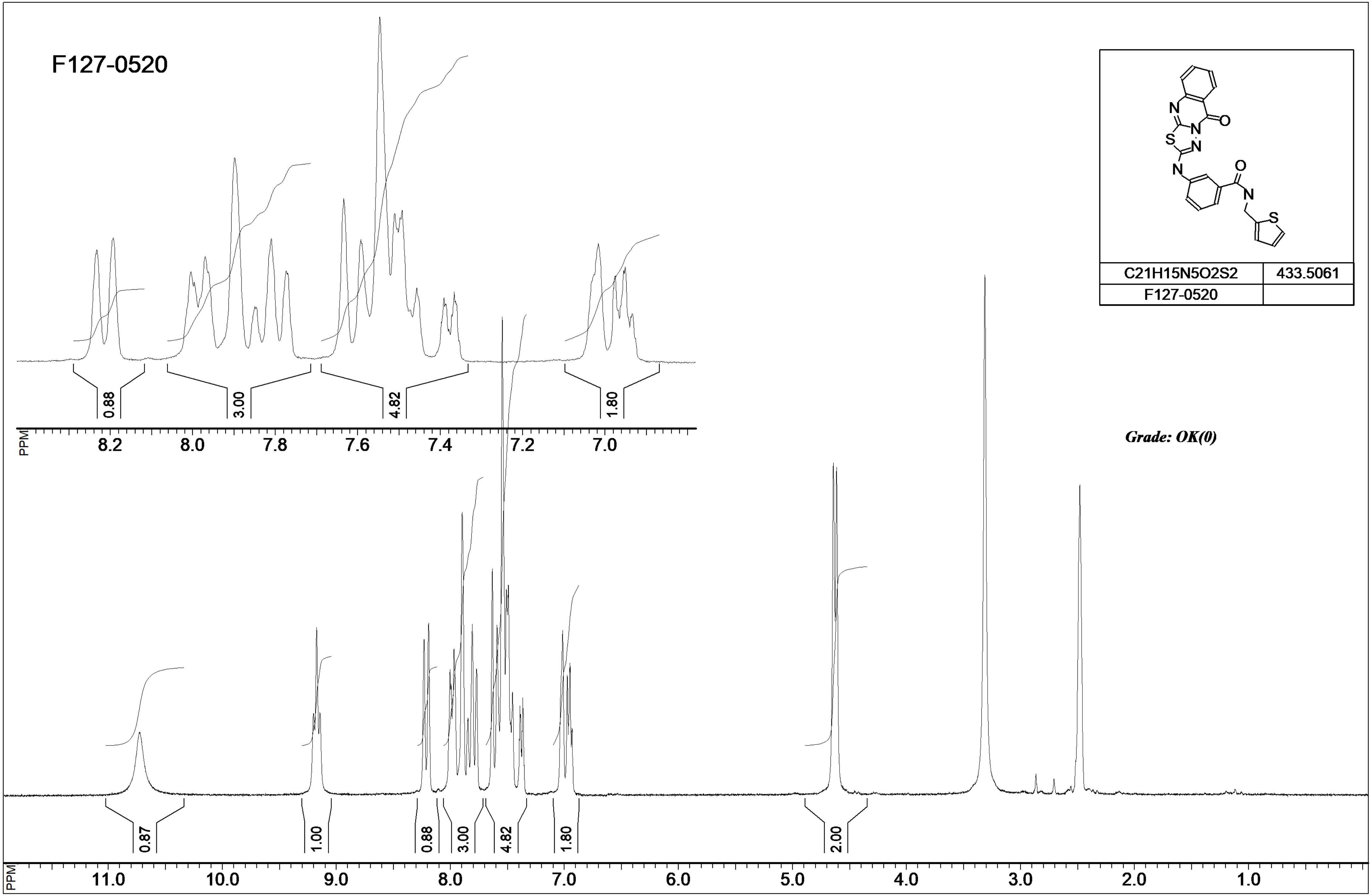

**File name: F127-0520**	Operator:	SF: 199.9703 MHz	NSC: 0	PW: 7.00 usec, RG: 26	Grade: OK(0)
Date: 11-May-2007	Solvent: DMSO	SW: 5000 Hz	TE: 295 K	AQ: 1.99 sec, RD: 0.00 sec	STANDARD 1H OBSERVE
