# Supplementary material for: Identification of Novel CB2 Ligands through Virtual Screening and In Vitro Evaluation
Source: J Chem Inf Model. 2023 Jan 24;63(3):1012–27. doi: 10.1021/acs.jcim.2c01503 (PMC9930120; doi:10.1021/acs.jcim.2c01503)
Supplement: Supplementary file 2 — ci2c01503_si_002.zip [file ci2c01503_si_002.zip › F6411-1421.pdf]

-.o.-Syntez Purity Report -.o.-

Agilent 1100 LC/MSD SL
Diodearrey G1315B (DAD1A-215nm; DAD1B-254nm)
Mass Quad G1956B (MSD1-Pos, MSD2-Neg)
ELSD Altech 3300 (ADC1 A, ELSD)

Mobile Phase:A-H2O+0.1%HCOOH;B-MeCN+0.1HCOOH
Separation column:
Rapid Resolutionn HT Cartige 4.6x30mm,
1.8-Micron, Zorbx SB-C18

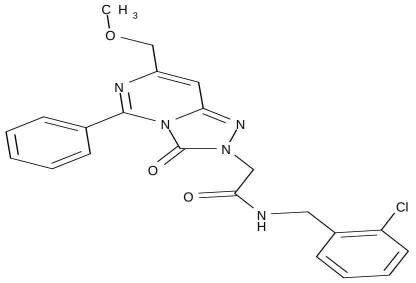

Mol.Weight: 437.89

F6411-1421

M10962 ->

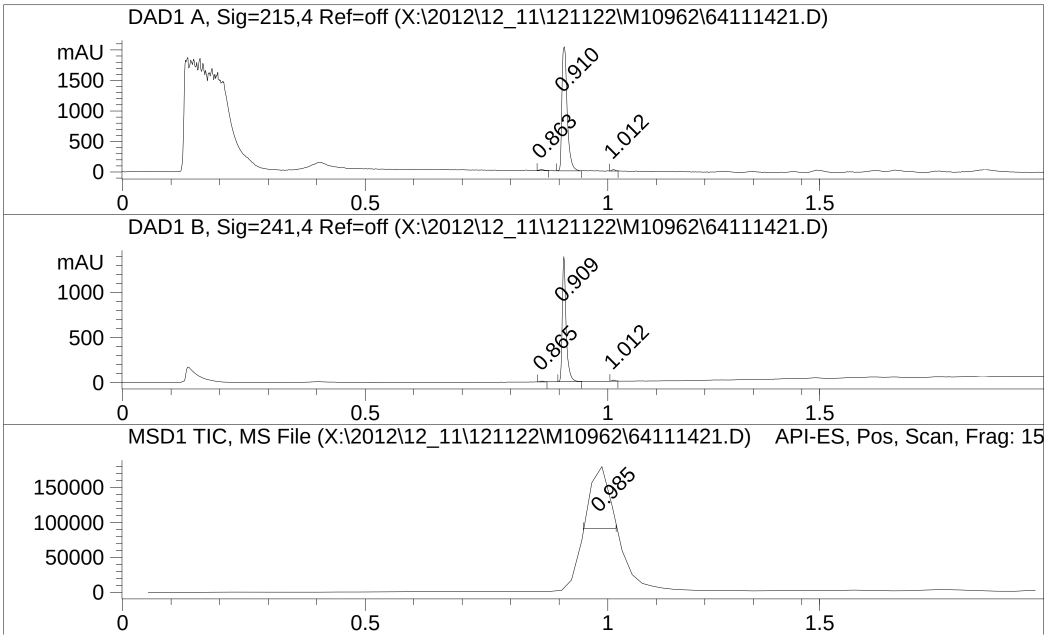

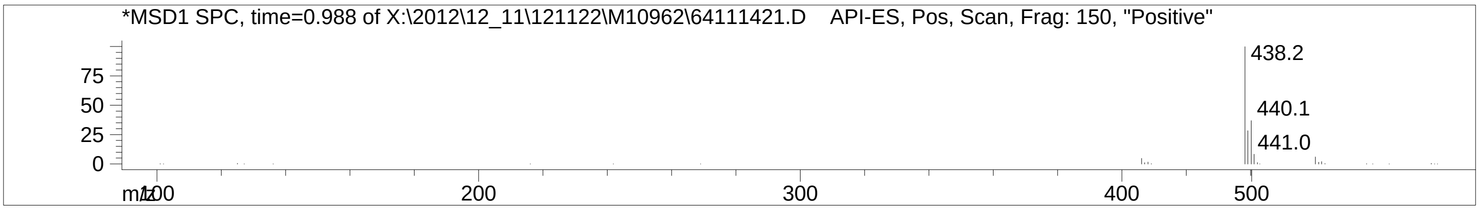

#	Signal	R.Time	Area %
1	DAD1 A, Sig=215,4 Ref=off	0.863	0.932
2		0.910	98.221
3		1.012	0.848

#	Signal	R.Time	Area %
1	DAD1 B, Sig=241,4 Ref=off	0.865	0.806
2		0.909	98.358
3		1.012	0.836

#	Signal	R.Time	Area %
1	MSD1 TIC, MS File	0.985	100.000
