# Supplementary material for: Identification of Novel CB2 Ligands through Virtual Screening and In Vitro Evaluation
Source: J Chem Inf Model. 2023 Jan 24;63(3):1012–27. doi: 10.1021/acs.jcim.2c01503 (PMC9930120; doi:10.1021/acs.jcim.2c01503)
Supplement: Supplementary file 2 — ci2c01503_si_002.zip [file ci2c01503_si_002.zip › J084-0328.pdf]

J084-0328

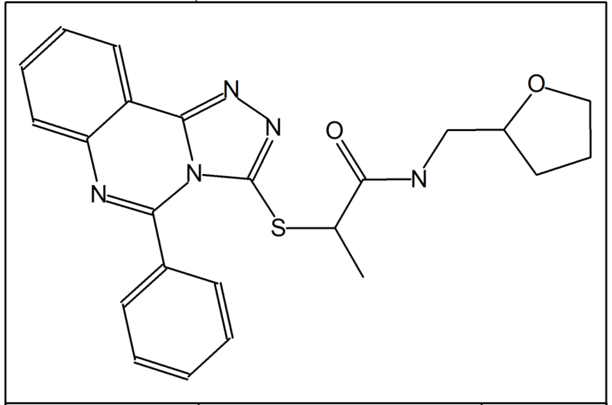

J084-0328	C23H23N5O2S	433.53

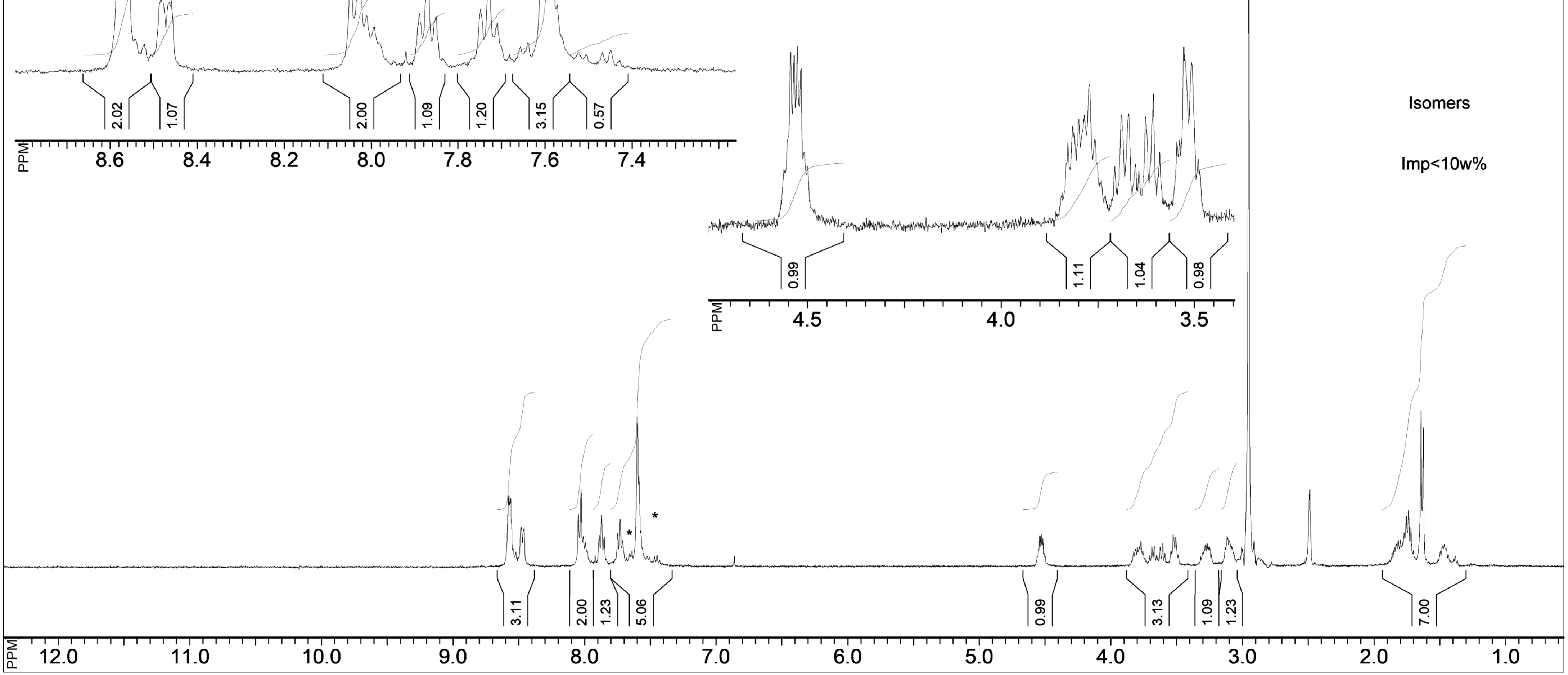

File name: J084-0328	Operator: ChSZR, Expert: PEB	SF: 399.9525 MHz	NSC: 0	PW: 6.00 usec, RG: 20, SI: 16384	Grade: OK(10)
Date: 18-May-2007	Solvent: DMSO-d6 +CCl4	SW: 7502 Hz	TE: 300 K	AQ: 1.09 sec, RD: 1.50 sec	* J084- 0328. 18- May- 2007. 10*
