# Supplementary figures and images for: Identification of Novel CB2 Ligands through Virtual Screening and In Vitro Evaluation
Source: J Chem Inf Model. 2023 Jan 24;63(3):1012–27. doi: 10.1021/acs.jcim.2c01503 (PMC9930120; doi:10.1021/acs.jcim.2c01503)

F3167-1559

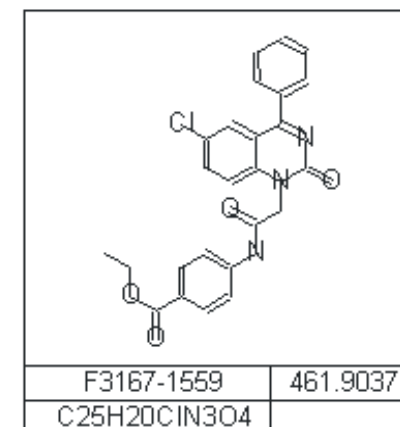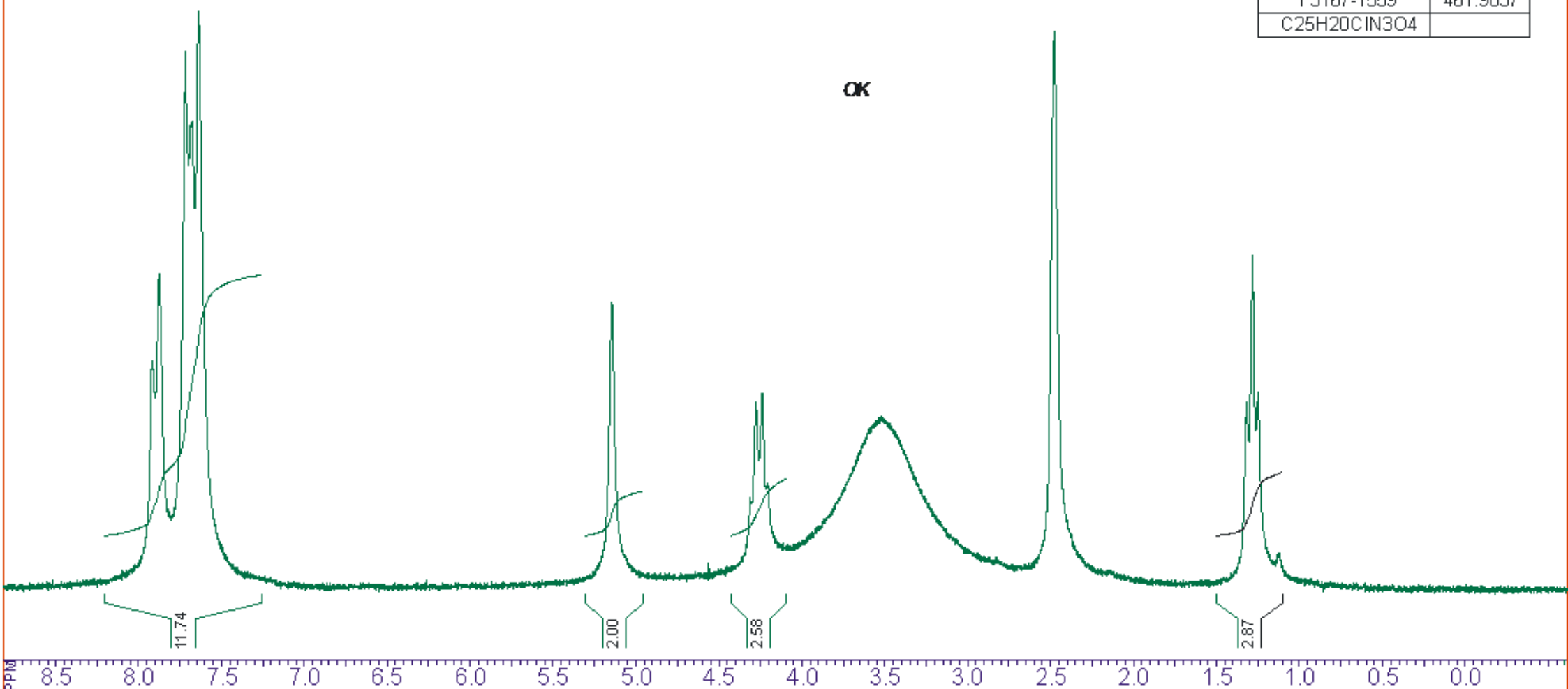

|                       |               |                  |           |                            |        |
|-----------------------|---------------|------------------|-----------|----------------------------|--------|
| File name: F3167-1559 | Operator: MVI | SF: 199.9703 MHz | NSC: 0    | PW: 7.00 usec, RG: 24      | Grade: |
| Date: 07-Jun-2005     | Solvent: DMSO | SW: 5000 Hz      | TE: 298 K | AQ: 1.99 sec, RD: 0.00 sec |        |

Supplement: Supplementary file 2 — ci2c01503_si_002.zip [file ci2c01503_si_002.zip › F3167-1559.pdf]

Bruker AV-500, SF=500.13 MHz, 03-03-2021 Base: BBB7720-40

IVX46565 in DMSO-d6/CCl4

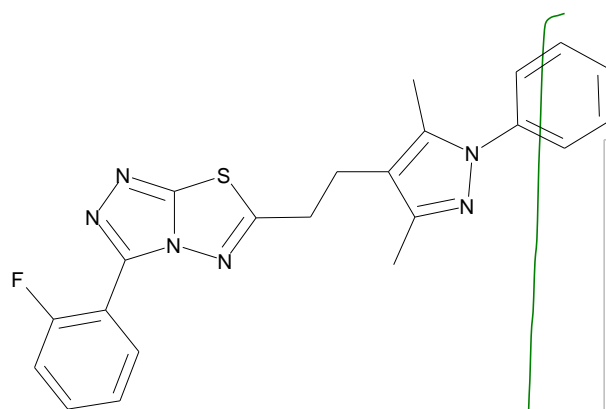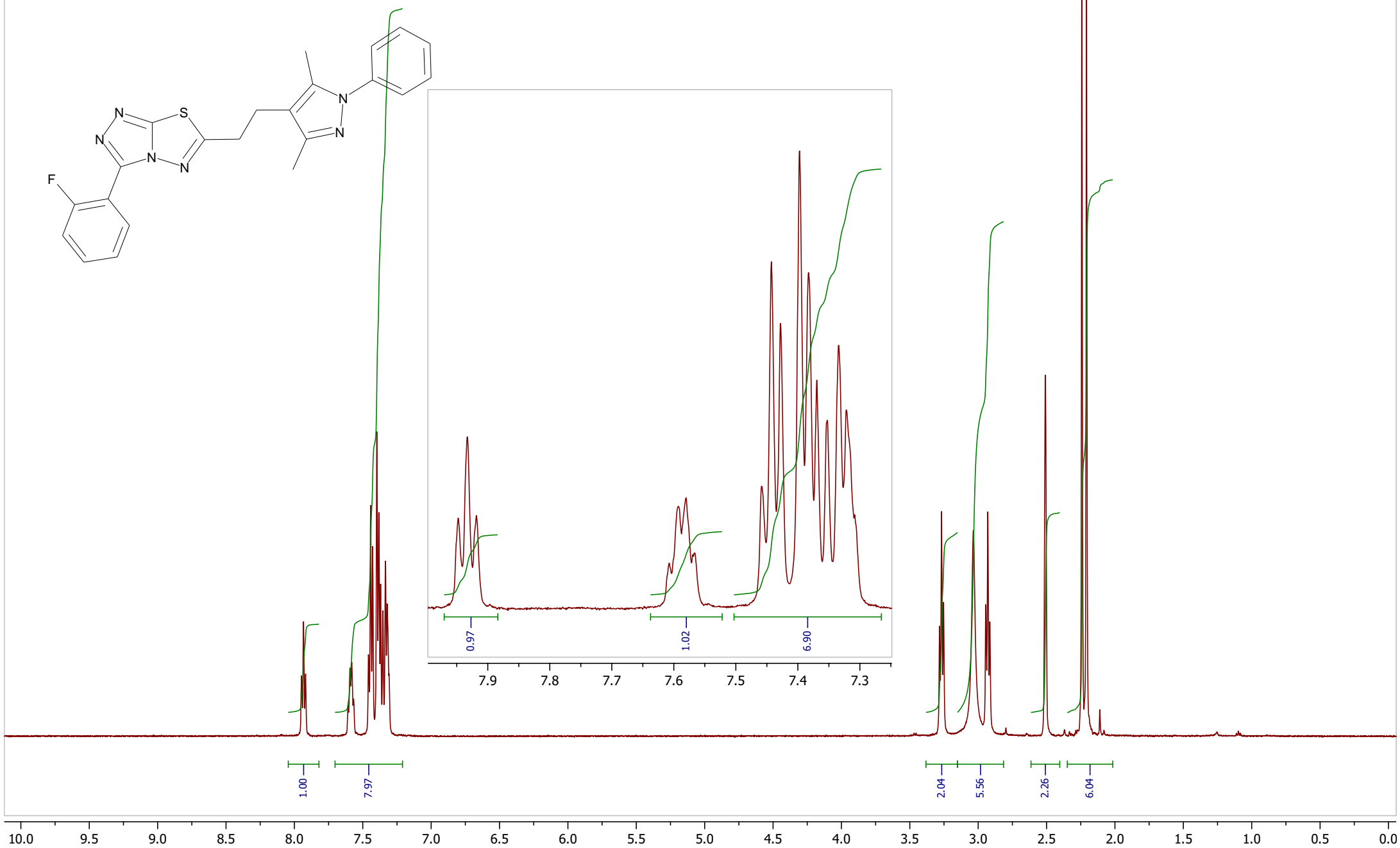

Supplement: Supplementary file 2 — ci2c01503_si_002.zip [file ci2c01503_si_002.zip › STK643755.PDF]

Bruker AV-500, SF=500.13 MHz, 03-03-2021 Base: BBB7720-4

FRS12854 in CDCl<sub>3</sub>

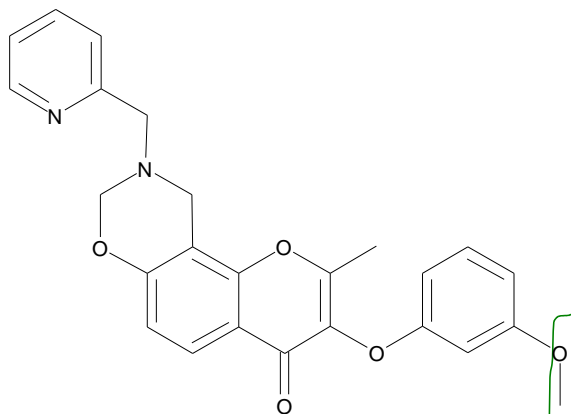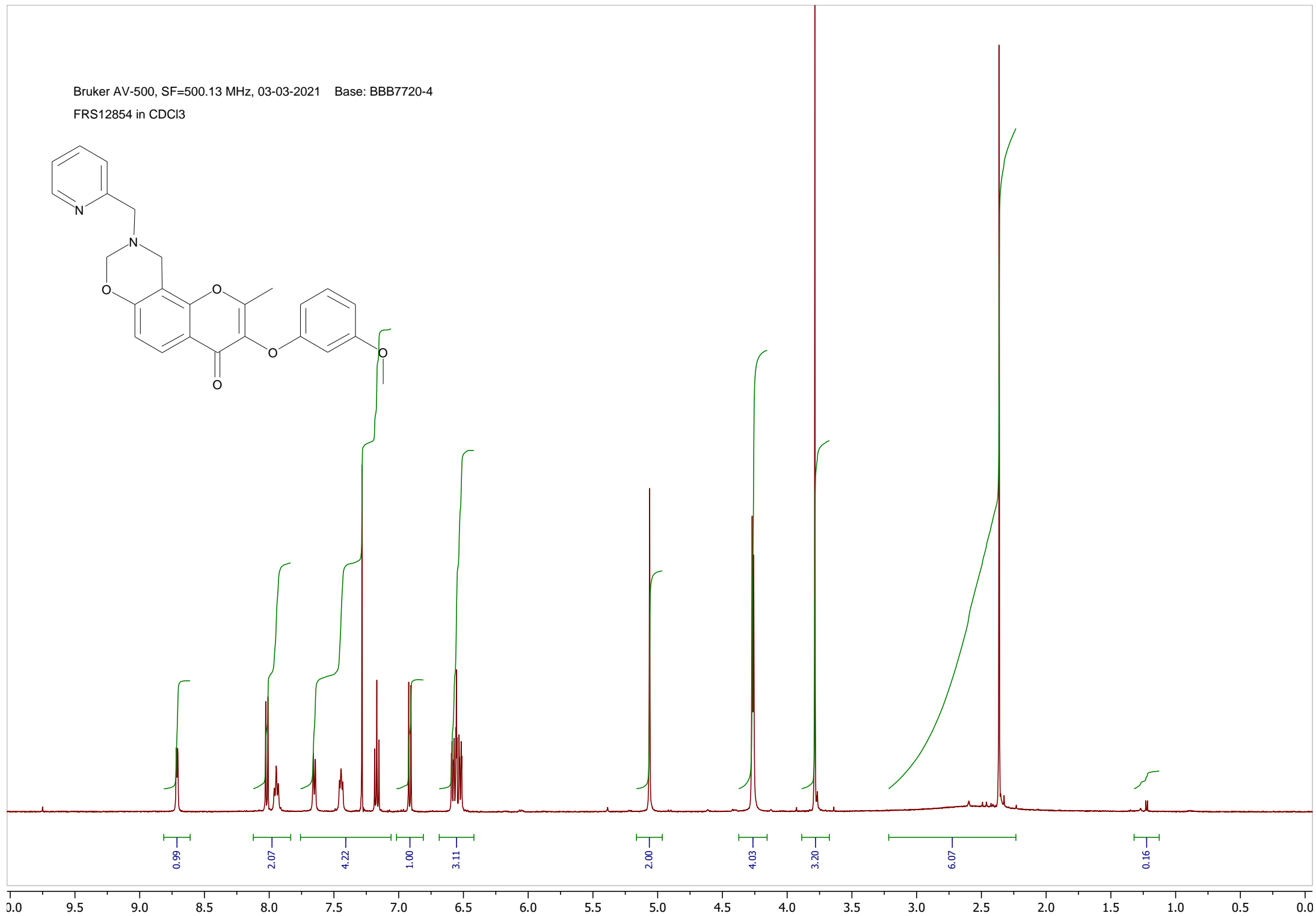

Supplement: Supplementary file 2 — ci2c01503_si_002.zip [file ci2c01503_si_002.zip › STL099362.PDF]

Bruker AC-200, SF=200.13 MHz, 07-10-2022 Base: BBB8438-3

UGS17987 in DMSO-d<sub>6</sub>/CCl<sub>4</sub>

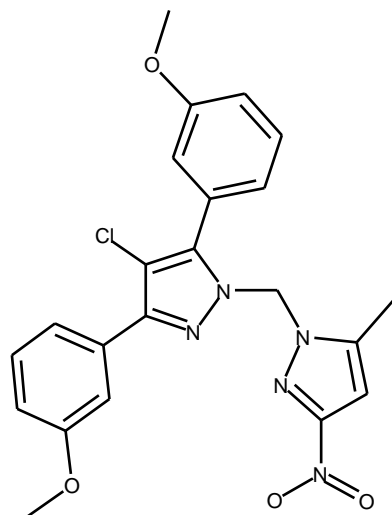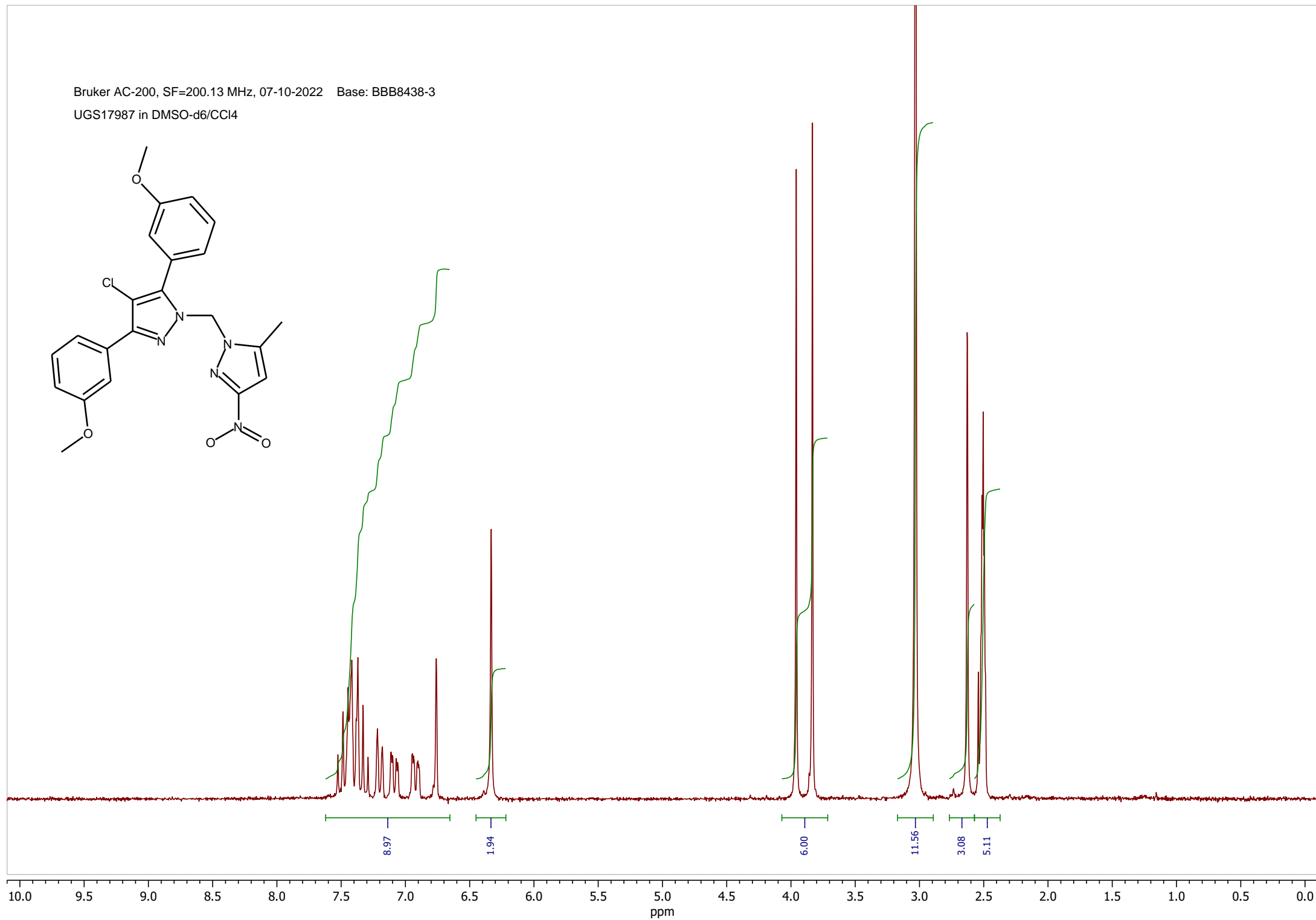

Supplement: Supplementary file 2 — ci2c01503_si_002.zip [file ci2c01503_si_002.zip › STL414105.pdf]

Bruker AC-200, SF=200.13 MHz, 07-10-2022 Base: BBB8438-3

UGS20017 in DMSO-d6/CCl4

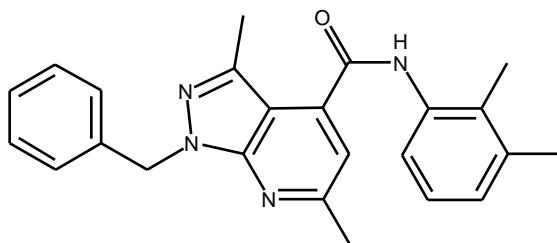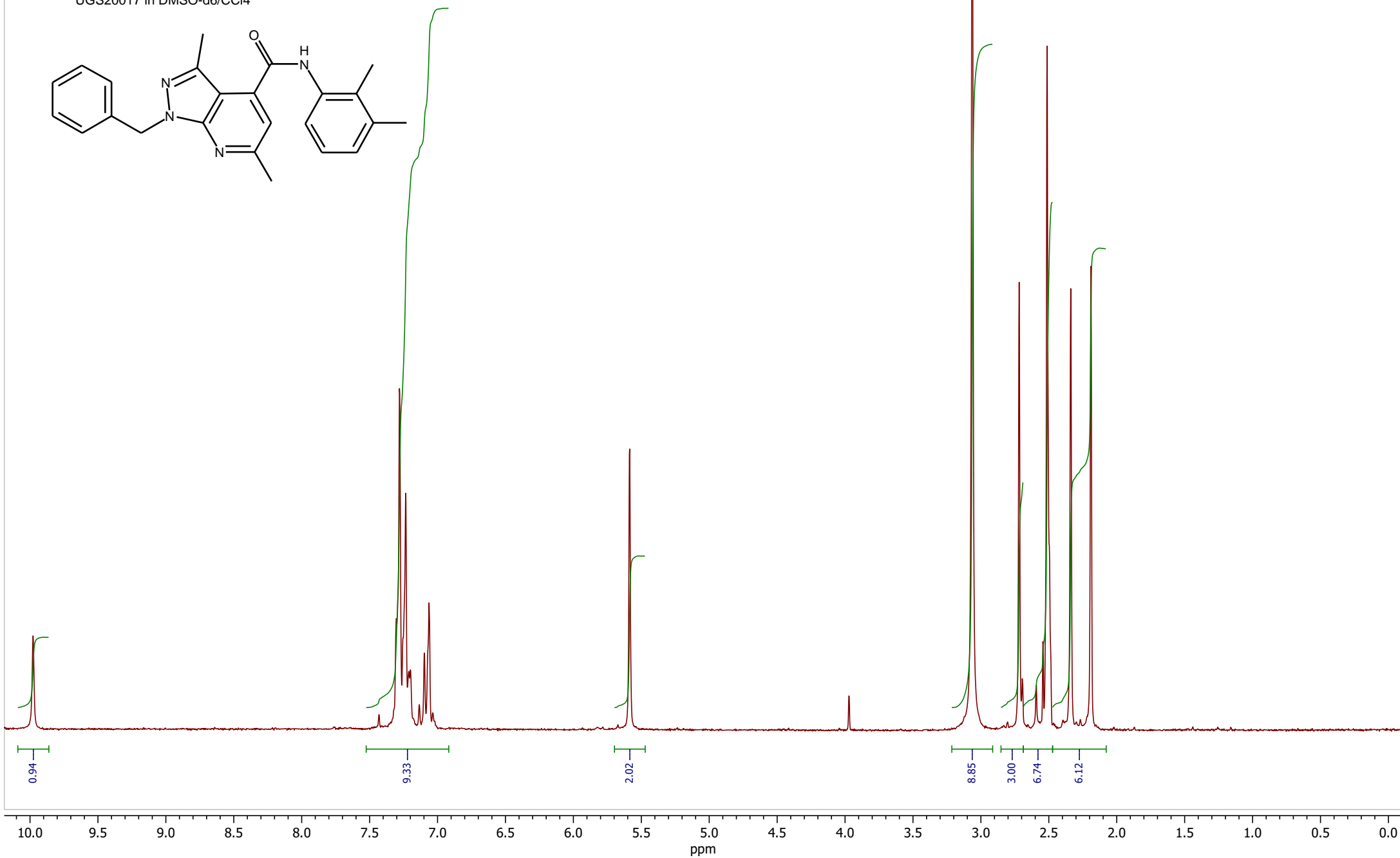

Supplement: Supplementary file 2 — ci2c01503_si_002.zip [file ci2c01503_si_002.zip › STL414201.pdf]

P2001S-504417

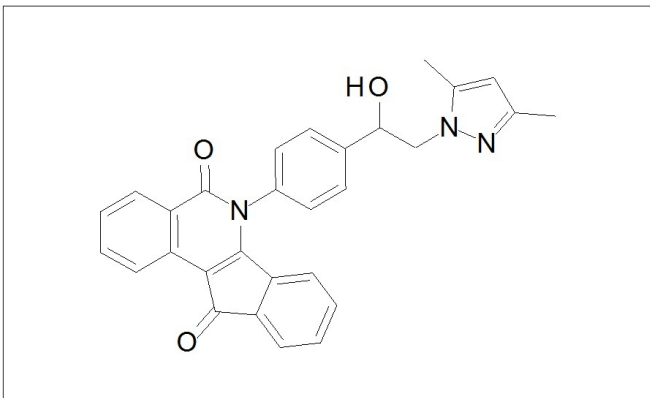

G74691

MAVAS-212850

C<sub>29</sub>H<sub>23</sub>N<sub>3</sub>O<sub>3</sub>

461.52

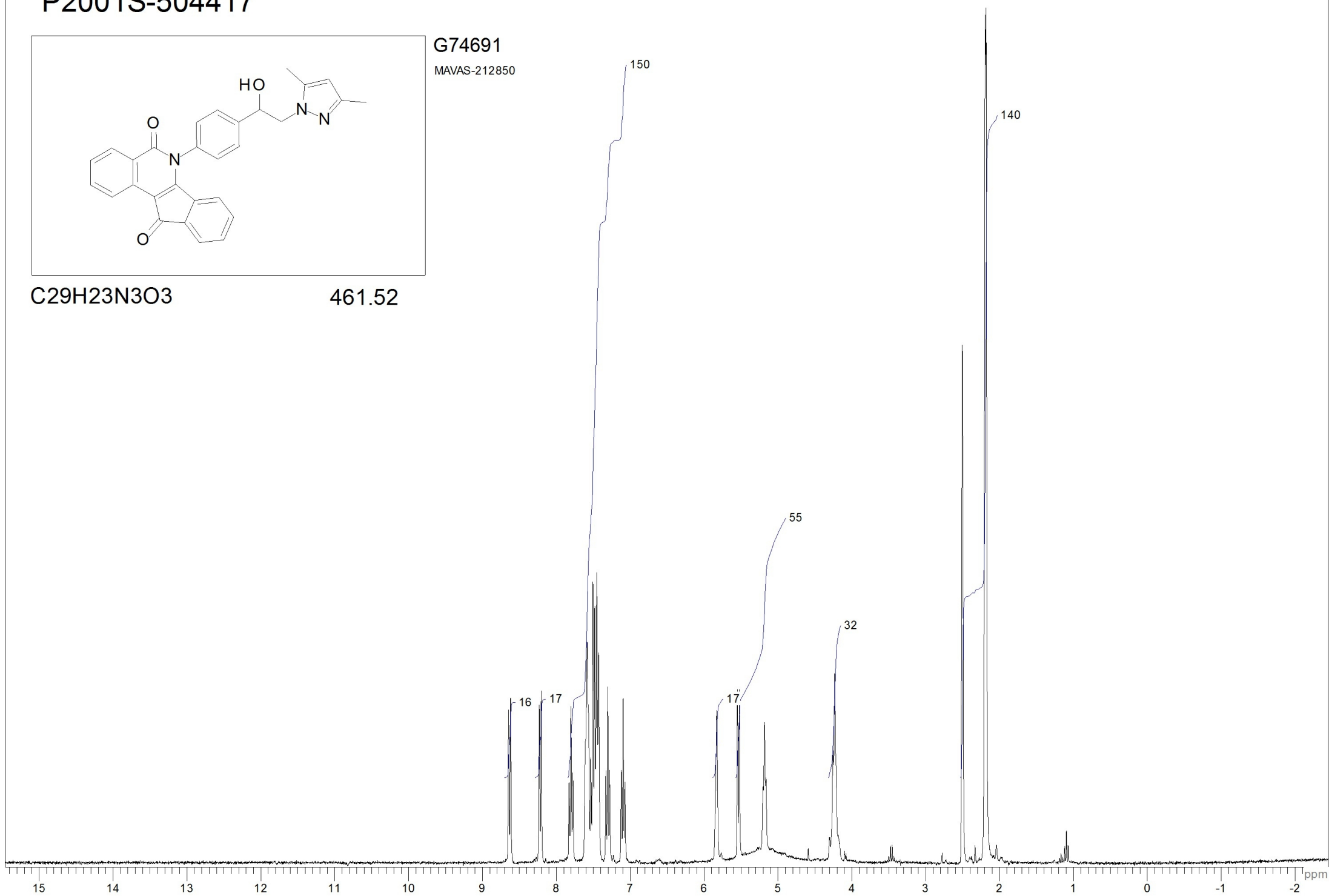

Supplement: Supplementary file 2 — ci2c01503_si_002.zip [file ci2c01503_si_002.zip › STL569224.pdf]
